# Rates and time trends in the consumption of breastmilk, formula, and animal milk by children younger than 2 years from 2000 to 2019: analysis of 113 countries

**DOI:** 10.1016/S2352-4642(21)00163-2

**Published:** 2021-09

**Authors:** Paulo A R Neves, Juliana S Vaz, Fatima S Maia, Philip Baker, Giovanna Gatica-Domínguez, Ellen Piwoz, Nigel Rollins, Cesar G Victora

**Affiliations:** a International Center for Equity in Health, Universidade Federal de Pelotas, Pelotas, Brazil; b Faculty of Nutrition, Universidade Federal de Pelotas, Pelotas, Brazil; c Universidade Federal do Rio Grande (FURG), Rio Grande, Brazil; d Institute for Physical Education and Nutrition, Deakin University, Melbourne, VIC, Australia; e Global Development Program, Bill & Melinda Gates Foundation, Seattle, WA, USA; f Department of Maternal, Newborn, Child, and Adolescent Health, WHO, Geneva, Switzerland

## Abstract

**Background:**

Previous analyses of trends in feeding indicators of children younger than 2 years have been limited to low-income and middle-income countries. We aimed to assess time trends in the consumption of different types of milk (breastmilk, formula, and animal milk) by children younger than 2 years from 2000 to 2019 at a global level.

**Methods:**

In this time-series analysis, we combined cross-sectional data from 487 nationally representative surveys from low-income and middle-income countries and information from high-income countries to estimate seven infant and young child feeding indicators in up to 113 countries. Multilevel linear models were used in pooled analyses to estimate annual changes in feeding practices from 2000 to 2019 for country income groups and world regions.

**Findings:**

For the absolute average annual changes, we found significant gains in any breastfeeding at age 6 months in high-income countries (1·29 percentage points [PPs] per year [95% CI 1·12 to 1·45]; p<0·0001) and at age 1 year in high-income countries (1·14 PPs per year [0·99 to 1·28]; p<0·0001) and upper-middle-income countries (0·53 PPs per year [0·23 to 0·82]; p<0·0001). We also found a small reduction in low-income countries for any breastfeeding at age 6 months (−0·07 PPs per year [–0·11 to −0·03]; p<0·0001) and age 1 year (−0·13 PPs per year [–0·18 to −0·09]; p<0·0001). Data on exclusive breastfeeding and consumption of formula and animal milk were only available for low-income and middle-income countries, where exclusive breastfeeding in the first 6 months of life increased by 0·70 PPs per year (0·51–0·88; p<0·0001) to reach 48·6% (41·9–55·2) in 2019. Exclusive breastfeeding increased in all world regions except for the Middle East and north Africa. Formula consumption in the first 6 months of life increased in upper-middle-income countries and in east Asia and the Pacific, Latin America and the Caribbean, the Middle East and north Africa, and eastern Europe and central Asia, whereas the rates remained below 8% in sub-Saharan Africa and south Asia. Animal milk consumption by children younger than 6 months decreased significantly (−0·41 PPs per year [–0·51 to −0·31]; p<0·0001) in low-income and middle-income countries.

**Interpretation:**

We found some increases in exclusive and any breastfeeding at age 6 months in various regions and income groups, while formula consumption increased in upper-middle-income countries. To achieve the global target of 70% exclusive breastfeeding by 2030, however, rates of improvement will need to be accelerated.

**Funding:**

Bill & Melinda Gates Foundation, through WHO.

## Introduction

WHO recognises that breastfeeding is essential for achieving optimal child growth, development, and health. The organisation recommends that children initiate breastfeeding within the first hour of life, breastfeed exclusively for the first 6 months, and thereafter receive adequate and safe complementary foods while breastfeeding continues up to age 2 years or beyond.^1^, ^2^ For children, breastfeeding protects against infections and malocclusion; is associated with increases in intelligence quotient points in childhood, adolescence, and adulthood; and is likely to reduce the risk of overweight and diabetes.^3^ Women who breastfeed their children have a reduced risk of ovarian and breast cancer, and breastfeeding also promotes spacing out births.^3^ Although some parents are unwilling or unable to breastfeed their children, breastfeeding is regarded as an essential component of optimal nurturing care for child health and development globally.^3^, ^4^ Suboptimal breastfeeding is estimated to cause almost 600 000 annual child deaths from pneumonia and diarrhoea alone and the death of nearly 100 000 women from breast and ovarian cancer and type 2 diabetes.^5^

Breastfeeding is more prevalent in low-income and middle-income countries than in high-income countries,^3^ being inversely associated with national gross domestic product.^6^ Within low-income and middle-income countries, continued breastfeeding at age 1 and 2 years is inversely associated with household socioeconomic status.^6^ By contrast, the prevailing pattern within high-income countries is the opposite, with women's education being positively associated with duration of breastfeeding.^7^

**Research in context**

**Evidence before this study**

We searched PubMed and Google Scholar with the following combination of keywords: trends AND (“breastfeeding” OR “breast feeding”) OR (“breast milk substitutes”) AND (“nutrition surveys” OR “health surveys”), without any language restriction from inception until Jan 31, 2021. We also searched the UNICEF and WHO websites with similar terms, for the same time frame. We identified a few multi-country studies describing trends in feeding indicators, all restricted to low-income and middle-income countries, except for an analysis that used nationally representative surveys from countries classified as high income only. No pooled analyses including low-income, middle-income, and high-income countries were identified. Overall, increases in breastfeeding indicators were reported in most studies, particularly for exclusive breastfeeding among children younger than 6 months. In 2019, UNICEF reported that exclusive breastfeeding increased from 35% in 2005 to 42% in 2018 in 80 low-income and middle-income countries. Regarding formula consumption, a single 2021 article was identified, which reported increasing trends in four of six Latin American countries for children younger than 1 year between 1990 and 2017. We did not locate any multi-country analysis on trends in the consumption of animal milk by young children.

**Added value of this study**

Using 487 data points from 113 countries, we analysed global trends from 2000 to 2019 for seven feeding indicators among children younger than 2 years, using available data from low-income, middle-income, and high-income countries. Pooled global analysis showed that the proportions of children breastfed at age 6 months and 1 year were stable over time, with no significant changes at a national level. However, increases in these breastfeeding indicators were observed in high-income countries and in upper-middle-income countries, while low-income countries presented small but significant declines. Because few high-income countries had nationally representative information on exclusive breastfeeding or on consumption of formula and animal milk, these analyses were restricted to low-income and middle-income countries with standardised survey data. In these countries, exclusive breastfeeding prevalence increased at a national level and in all world regions, except in the Middle East and north Africa. The pooled estimate for 83 low-income and middle-income countries with data was 48·6% in 2019. Formula milk consumption under age 6 months and at 6–23 months remained unchanged for low-income and middle-income countries as a whole, but we found important increases in upper-middle-income countries and in some regions—the Middle East and north Africa, Latin America and the Caribbean, eastern Europe and central Asia, and especially east Asia and the Pacific. For low-income and middle-income countries, small but significant reductions were observed for the consumption of animal milk by children younger than 6 months, but not at older ages.

**Implications of all the available evidence**

Despite positive trends in exclusive breastfeeding, the rate of improvement is insufficient for reaching the global target of at least 70% global prevalence by 2030. Findings for any breastfeeding in high-income countries and upper-middle-income countries are encouraging, but renewed efforts are needed to counteract declining trends in low-income societies where undernutrition and child mortality rates are still high, and where children can most benefit from continued breastfeeding. The rapid increase in formula milk consumption in all age ranges in upper-middle-income countries is alarming. This is the most comprehensive analysis on this topic to date, revealing the urgent need to place breastfeeding support, promotion, and protection as national priorities, and to halt the marketing and sale of infant formula by enforcing the recommendations of the International Code of Marketing of Breastmilk Substitutes.

In the past two decades, global data^8^ have shown improvements in some but not all breastfeeding indicators. For instance, the rate of exclusive breastfeeding in the first 6 months of age increased from 35% in 2005 to 42% in 2018 in low-income and middle-income countries.^9^ Nevertheless, in most low-income and middle-income countries, exclusive breastfeeding remains below the global targets of 50% by 2025 and 70% by 2030.^10^ Additionally, several countries have reported important increases in the total duration of breastfeeding since the 1990s, including Bolivia, Brazil, Colombia, Peru, the USA, and most sub-Saharan African countries.^11^, ^12^, ^13^, ^14^

Despite these improvements in breastfeeding indicators, sales of commercial breastmilk substitutes (eg, milk-based formula) have increased globally, especially in upper-middle-income countries.^6^, ^9^, ^15^ Such products include any milk in either liquid or powder form that are marketed specifically to feed children aged up to 3 years, including infant or standard (age 0–6 months), follow-up (7–12 months), and toddler (13–36 months) formula.^16^ An analysis showed that between 2005 and 2019, world total formula sales grew by 121·5%, with further increases of 10·8% projected by 2024.^15^ By contrast, exclusive breastfeeding increased by only 20% in the same period.^9^ Increased formula milk sales was led mainly by upper-middle-income countries and countries in east Asia and the Pacific, eastern Europe, central Asia, the Middle East, north Africa, Latin America, and the Caribbean.^15^

Monitoring breastfeeding progress helps to understand the need for and effect of policies and programmes and to design new interventions targeted at improving the health of women and children.^13^ Yet, most data on breastfeeding practices are restricted to low-income and middle-income countries because most high-income countries do not report on internationally standardised indicators.^1^, ^17^ Additionally, although data on formula sales are available from sources such as Euromonitor, similar data on consumption of these products by children are not available for comparison.^15^ Lastly, no multi-country studies are available on the consumption of non-human milk other than formula in children younger than 2 years.

Aiming to address these gaps, we examined time trends and worldwide patterns in the consumption of different types of milk (breastmilk, formula, and animal milk) by children aged under 2 years, in 113 countries from 2000 to 2019.

## Methods

### Data sources

For countries with standardised surveys, most of which are low-income and middle-income countries, we analysed nationally representative surveys, namely Demographic Health Surveys (DHS), Multiple Indicator Cluster Surveys (MICS), and Reproductive Health Surveys (RHS). These surveys are highly comparable in terms of questionnaires, field procedures, and sampling design, which results in harmonised estimates through close collaboration with the UN's interagency groups. DHS and MICS collect data on key indicators of reproductive, maternal, newborn, and child health and nutrition, and are regularly updated.^18^ We also included the nationally representative surveys done in Peru (Encuesta Demográfica y de Salud Familiar) and Ecuador (Encuesta Nacional de Salud y Nutrición), after harmonising their datasets and variables according to the DHS and MICS standards. In all surveys, information regarding reproductive, maternal, newborn, and child health and nutrition indicators were collected at a household level through face-to-face interviews with women of childbearing age (15–49 years) using standardised questionnaires administered by trained field workers. Information on feeding practices was collected for the youngest child aged under 2 years in each household using a qualitative 24-h food recall.^1^ For DHS, data were available starting in 1993; for MICS, in 2005; and for RHS, in 1991.

For countries without standardised surveys, a comprehensive literature search was done until Nov 30, 2019, to locate health services data on breastfeeding indicators that were nationally representative. These countries included high-income countries and a few upper-middle-income countries, according to the World Bank's income classification in the fiscal year 2018–19.^19^ In addition to searching databases such as PubMed and Google Scholar, we did a systematic search of the grey literature up to Nov 30, 2019. Additionally, we consulted the reference lists in all documents to identify past publications, activated email alerts from scientific journals in the nutrition and public health field, and emailed breastfeeding associations, research institutions, government institutions of health, and experts in the area from several countries. We sought data from nationally representative, population-based surveys or health reports from electronic surveys or medical records (eg, maternity hospitals and primary health care) with sufficiently detailed methodological descriptions (assessed by JSV and FSM). We only incorporated breastfeeding rates published in country card reports and websites from international agencies and civil society organisations after accessing the original complete references.^17^ Data from the literature review were available starting in 1995. The search terms used and the list of references included are described in the appendix (pp 1–3).

### Data analysis

We investigated seven feeding indicators using the data sources described. These indicators are defined in table 1 both according to WHO^1^ and by non-standard definitions, to allow comparability across countries, mostly because high-income countries do not report breastfeeding indicators according to international standardised definitions.^1^, ^3^, ^6^, ^17^

**Table 1:** Definitions of feeding indicators

	**Definition**	**Number of countries with information**
Any breastfeeding at age 6 months	For countries without standardised surveys*: proportion obtained directly from the reports.^16^ For countries with standardised surveys† : proportion of breastfed children among those aged 4–7 months (midpoint 6 months)^3^	110
Any breastfeeding at age 1 year	For countries without standardised surveys*: proportion obtained directly from the reports.^16^ For countries with standardised surveys† : proportion of breastfed children among those aged 10–13 months (midpoint 12 months)^3^	105
Exclusive breastfeeding at age <6 months	For countries with standardised surveys† : proportion of children aged 0–5 months who were exclusively breastfed^1^	83
Formula consumption at age <6 months	For countries with standardised surveys† : proportion of children aged 0–5 months who were fed formula^6^	83
Formula consumption at age 6–23 months	For countries with standardised surveys† : proportion of children aged 6–23 months who were fed formula^6^	83
Animal milk consumption at age <6 months	For countries with standardised surveys† : proportion of children aged 0–5 months who were fed animal milk; interviewers asked about consumption of animal milk (fresh, tinned, or powdered) and specifically excluded products that were marketed as formula^6^	83
Animal milk consumption at age 6–23 months	For countries with standardised surveys† : proportion of children aged 6–23 months who were fed animal milk; interviewers asked about consumption of animal milk (fresh, tinned, or powdered) and specifically excluded products that were marketed as formula^6^	83

To standardise the available information, using data from low-income and middle-income countries we calculated the feeding indicators for children aged 4–7 months, which represents the midpoint 6 months.

* Included high-income and some upper-middle-income countries without standardised surveys.

† Included low-income and middle-income countries with standardised surveys.

On the basis of available data, we investigated any breastfeeding at age 6 months and at 1 year. 113 countries had information on any breastfeeding, including 110 countries with information for any breastfeeding at age 6 months and 105 countries with information for age 1 year. Breastfeeding was recorded at 1 year rather than 6–23 months because of the availability of data. Using data from nationally representative surveys done in low-income and middle-income countries, we calculated exclusive breastfeeding in infants younger than 6 months,^1^ formula milk consumption in children younger than 6 months and those aged 6–23 months, and animal milk consumption in children younger than 6 months and aged 6–23 months. Children were classified as receiving formula or animal milk when any consumption of these foodstuffs was mentioned, regardless of daily frequency. We checked the consistency of our recalculated estimates of exclusive breastfeeding with published figures in survey reports. Small discrepancies (<1 percentage point [PP]) were observed for a few surveys, occurring mostly when some food groups were not taken into account to generate the estimate provided in the report, mostly because of country-specific foods not being considered (eg, insects). Our estimates avoided such bias by considering only children who did not receive any type of food, however uncommon, as exclusively breastfed. Following international recommendations, missing values and “don't know” options were considered “not consumed”.^20^ 83 low-income and middle-income countries with available data on all five indicators were included in these analyses. Because of a lack of standardised data, these indicators were not calculated for high-income countries.

We included countries with at least two data points with an interval of 5 or more years. Initial analyses were done at a national level, followed by grouping countries by World Bank income level^19^ and by world regions, as per UNICEF classification.^21^ 487 data points, including data from the literature search, were available. Availability of data points was as follows: 481 data points from 110 countries for any breastfeeding at age 6 months, 445 data points from 105 countries for any breastfeeding at age 1 year, 344 data points from 83 low-income and middle-income countries for exclusive breastfeeding in children younger than 6 months, 328 data points from 83 low-income and middle-income countries for formula consumption in children younger than 6 months and those aged 6–23 months, and 342 data points from 83 low-income and middle-income countries for animal milk consumption in children younger than 6 months and those aged 6–23 months. The appendix shows a world map showing all countries analysed (p 4) and flowcharts for the literature search of high-income country reports and selecting countries included in the analyses (pp 5–7).

### Statistical analysis

Descriptive analyses were done at national, world region, and country income group levels. The proportions of countries that provided data for each region and country income group were estimated. We used multilevel linear regression to estimate average absolute annual changes in PPs for each feeding indicator, with country as the highest hierarchical level and year as the second level. Fractional polynomials were used to investigate departures from linearity. However, the polynomials did not show any improvement compared with linear regression, which was therefore used for simplicity. Available data points since the early 1990s were included in the analyses but trends are presented from 2000 to 2019, the period with the most data points. Line charts were used to illustrate changes over the 20-year period for each feeding indicator by country income groups and world regions.

All analyses were done by considering the cluster sample design of the surveys using the svy command in Stata (version 16.0). Furthermore, we weighted all analyses by the national population size of children within the specific age range for each indicator^22^ in the year that the survey was done. All analyses were done using Microsoft Excel worksheets and Stata (version 16.0).

Ethics approval was the responsibility of the institutions that administered the surveys and all analyses relied on anonymised databases.

### Role of the funding source

The funder of the study had no role in study design, data collection, data analysis, data interpretation, or writing of the report.

## Results

For data points with available information on sample sizes, the median number of children per survey increased from 371 (IQR 197–517) in 1991–99 to 455 (IQR 220–827) in 2000–09 and 678 (IQR 366–1540) in 2010–19 for children aged 6 months (or 4–7 months). For children aged 1 year (or 10–13 months), the median number of children per survey increased from 379 (IQR 210–509) in the 1990s, to 402 (IQR 180–628) in the 2000s, and 582 (IQR 261–1074) in the 2010s. For the formula and animal milk consumption indicators, which were based on data restricted to children aged 6–23 months in low-income and middle-income countries, the median number of children per survey rose from 1491 (1000–1878) in the 1990s, to 1760 (1100–2774) in the 2000s, and 2356 (1402–3600) in the 2010s. The data points included and the rates of the indicators analysed are given in the appendix (pp 9–52).

Country representativeness by UNICEF world regions was 22 (88%) of 25 countries in west and central Africa, 16 (67%) of 24 in eastern and southern Africa, seven (37%) of 19 in the Middle East and north Africa, 16 (76%) of 21 in eastern Europe and central Asia, six (75%) of eight in south Asia, ten (30%) of 33 in east Asia and the Pacific, 21 (57%) of 37 in Latin America and the Caribbean, one (50%) of two in North America, and 14 (42%) of 33 in western Europe. Using the World Bank's income classification for the median year of analysis (2010), we included 20 (28%) of 71 high-income, 27 (51%) of 53 upper-middle-income, 35 (63%) of 56 lower-middle-income, and 31 (89%) of 35 low-income countries. We analysed data from 93 (65%) of 144 low-income and middle-income countries, covering more than 70% of the population of children younger than 2 years in these countries.

**Figure 1:**
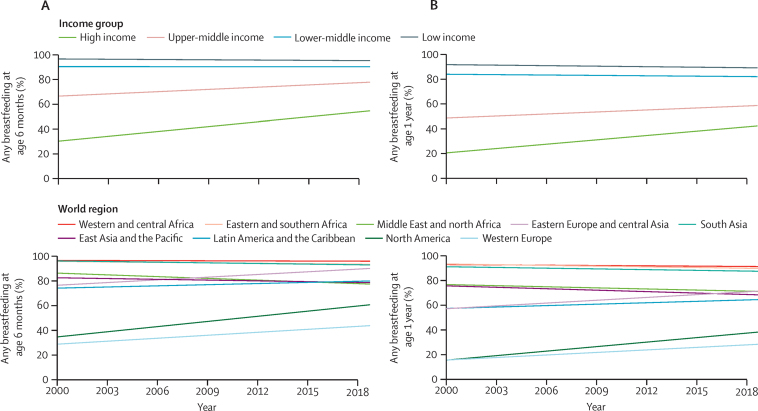
Evolution of the rates of any breastfeeding by age, income group, and world region, 2000–19 (A) Any breastfeeding at age 6 months (110 countries, based on 481 data points) and (B) any breastfeeding at 1 year (105 countries, based on 445 data points). Data on North America were restricted to the USA.

Table 2 presents the rates of the indicators in 2000 and 2019. Whereas at the global level both indicators of any breastfeeding did not change from 2000 to 2019, any breastfeeding at age 6 months increased in high-income countries (30·7% [95% CI 25·2–36·2] to 55·1% [46·9–63·4]) and upper-middle-income countries (67·0% [58·7–75·3] to 78·3% [68·2–88·4]). Similar increases were also observed for any breastfeeding at 1 year in these countries (table 2; figure 1). In both groups of countries, breastfeeding rates were lower than in poorer countries (table 2; figure 1). By contrast, rates remained virtually stable from 2000 to 2019 for lower-middle-income and low-income countries, with a slight reduction in low-income countries. When analysing the world regions, despite the increases in North America and western Europe, the rates in these two regions were well below that observed in the other regions (table 2; figure 1).

**Table 2:** Rates of infant and young child feeding indicators in 2000 and 2019, by country income groups and world regions

		**Any breastfeeding at age 6 months (95% CI)**		**Any breastfeeding at age 1 year (95% CI)**		**Exclusive breastfeeding at age <6 months (95% CI)**		**Formula consumption at age <6 months (95% CI)**		**Formula consumption at age 6–23 months (95% CI)**		**Animal milk consumption at age <6 months (95% CI)**		**Animal milk consumption at age 6–23 months (95% CI)**	
		2000	2019	2000	2019	2000	2019	2000	2019	2000	2019	2000	2019	2000	2019
All countries (n=83–110)		87·7% (79·8–95·7)	88·7% (84·2–93·1)	80·9% (71·4–90·8)	81·1% (74·3–87·4)	35·4% (27·4–43·3)	48·6% (41·9–55·2)	10·5% (5·7–15·4)	11·6% (5·7–17·5)	12·9% (9·3–16·5)	13·3% (8·3–18·4)	17·5 (13·6–21·5)	9·8% (6·1–13·5)	36·3% (26·2–46·4)	33·0 (25·1–40·9)
Country income group															
	High income (n=14–21)	30·7% (25·2–36·2)	55·1% (46·9–63·4)	21·3% (8·5–34·2)	42·9% (30·9–54·9)	NA	NA	NA	NA	NA	NA	NA	NA	NA	NA
	Upper-middle income (n=24–27)	67·0% (58·7–75·3)	78·3% (68·2–88·4)	49·1% (43·1–55·1)	59·0% (51·4–66·7)	16·8% (0·9–34·4)	37·0% (30·3–43·7)	25·3% (18·9–31·7)	36·0% (30·5–41·5)	20·9% (9·9–31·9)	39·1% (31·5–46·6)	24·8% (18·0–31·6)	7·6% (3·1–12·1)	63·7% (54·3–73·1)	37·2% (28·9–45·6)
	Lower-middle income (n=30–32)	90·9% (84·6–97·2)	90·8% (86·0–95·6)	84·0% (75·2–92·9)	82·1% (74·8–89·5)	35·4% (24·3–46·5)	46·7% (36·9–56·5)	13·8% (5·6–22·0)	12·8% (4·2–21·5)	14·4% (9·0–19·9)	15·0% (8·0–22·0)	19·9% (15·4–24·4)	9·9% (4·8–14·9)	40·7% (32·4–48·9)	34·0% (23·2–44·8)
	Low income (n=29–30)	97·1% (96·2–98·0)	95·8% (94·7–96·8)	91·7% (89·9–93·5)	89·2% (87·0–91·4)	36·8% (28·1–45·5)	51·2% (44·3–58·1)	7·7% (5·0–10·4)	5·2% (2·5–7·8)	11·2% (8·3–14·1)	5·1% (2·3–7·8)	16·8% (11·3–22·2)	12·6% (7·9–17·4)	33·6% (19·1–48·1)	35·1% (24·3–45·9)
World region															
	West and central Africa (n=21–22)	97·2% (95·8–98·6)	96·6% (96·1–97·1)	92·7% (89·2–96·2)	91·2% (89·0–93·2)	16·6% (10·6–22·6)	35·0% (22·3–47·7)	9·1% (6·3–11·9)	6·4% (4·9–7·9)	9·5% (7·9–11·1)	6·8% (5·2–8·4)	8·8% (6·3–11·3)	5·5% (3·7–7·4)	14·7% (11·6–17·9)	18·8% (13·6–24·1)
	Eastern and southern Africa (n=16)	96·4% (92·2–99·8)	94·1% (88·5–99·8)	93·2% (88·2–98·1)	89·6% (82·2–97·1)	39·2% (28·0–50·4)	60·8% (54·5–67·1)	7·6% (0·3–15·9)	3·8% (1·0–8·7)	8·1% (2·0–14·2)	4·2% (0·1–10·4)	14·4% (7·4–21·3)	5·7% (2·7–8·7)	24·8% (15·6–34·0)	18·2% (13·9–22·6)
	Middle East and north Africa (n=4–6)	87·0% (79·3–94·7)	78·1% (61·1–95·1)	76·6% (64·4–88·8)	71·1% (49·7–92·5)	42·9% (25·2–60·6)	30·2% (20·4–39·9)	14·4% (1·3–27·5)	26·7% (3·8–49·6)	16·0% (5·1–26·8)	18·3% (0·0–46·9)	13·6% (12·5–14·8)	7·1% (0·3–14·5)	48·4% (44·1–52·7)	30·3% (18·0–42·6)
	Eastern Europe and central Asia (n=16)	77·1% (68·9–85·3)	90·7% (86·2–95·3)	57·2% (46·0–68·3)	71·3% (61·5–81·1)	14·7% (11·5–18·0)	37·6% (32·8–42·4)	20·9% (16·3–25·4)	27·9% (20·1–35·8)	18·7% (10·9–26·5)	29·5% (21·0–37·9)	23·4% (17·6–29·3)	7·0% (2·1–12·0)	55·9% (40·9–70·9)	58·1% (44·9–71·3)
	South Asia (n=6)	96·6% (95·1–98·5)	93·6% (90·4–96·8)	90·8% (87·3–94·7)	87·3% (81·8–92·9)	44·9% (41·1–48·7)	54·7% (50·1–59·3)	5·9% (2·4–9·5)	5·7% (1·9–9·5)	12·3% (9·6–14·9)	7·7% (4·5–11·0)	23·5% (17·7–29·2)	15·5% (11·0–19·9)	48·9% (42·3–55·6)	42·8% (39·8–45·8)
	East Asia and Pacific (n=8–10)	83·2% (69·3–97·1)	79·3% (71·1–87·5)	75·6% (59·1–92·1)	68·7% (59·4–77·3)	31·8% (21·8–41·8)	40·0% (31·1–49·0)	22·9% (14·1–31·6)	38·3% (28·2–48·5)	25·9% (17·6–34·3)	45·4% (34·8–56·0)	12·7% (5·5–19·8)	2·0% (0·4–8·6)	26·0% (14·5–37·4)	26·7% (0·9–54·3)
	Latin America and the Caribbean (n=12–19)	74·8% (65·7–83·9)	80·6% (72·2–89·0)	57·4% (44·0–70·8)	64·5% (51·8–77·2)	37·3% (23·9–50·8)	51·7% (41·5–62·0)	24·7% (12·8–36·7)	29·2% (24·4–34·1)	14·1% (3·2–25·1)	30·8% (16·1–45·5)	17·4% (9·5–25·2)	9·7% (0·4–23·2)	42·6% (34·2–51·0)	39·8% (23·2–56·5)
	North America* (n=1)	35·3% (34·4–36·3)	61·4% (59·9–62·9)	15·8% (14·5–17·0)	38·3% (36·2–40·4)	NA	NA	NA	NA	NA	NA	NA	NA	NA	NA
	Western Europe (n=7–14)	29·5% (19·3–39·6)	44·5% (38·9–50·0)	15·8% (2·0–29·7)	28·5% (13·0–44·0)	NA	NA	NA	NA	NA	NA	NA	NA	NA	NA

n denotes range of countries. Countries grouped by UNICEF world regions and by World Bank income classification for 2010 as the median year of analysis. NA=not available.

* Data available only for the USA.

Table 3 shows the average absolute annual changes. For all countries combined, we found no significant changes over the period in the rate of any consumption of breastmilk at age 6 months or 1 year. This finding resulted from a combination of the following trends. We found significant gains in any breastfeeding at age 6 months in high-income countries (1·29 PPs per year [95% CI 1·12 to 1·45]; p<0·0001) and at age 1 year in high-income countries (1·14 PPs per year [0·99 to 1·28]; p<0·0001) and upper-middle-income countries (0·53 PPs per year [0·23 to 0·82]; p<0·0001; table 3). We also found a small reduction in low-income countries for any breastfeeding at age 6 months (−0·07 PPs per year [–0·11 to −0·03]; p<0·0001) and age 1 year (−0·13 PPs per year [–0·18 to −0·09]; p<0·0001; table 3). The rate of any breastfeeding at age 6 months significantly rose in eastern Europe and central Asia, North America, and western Europe, while decreasing in south Asia and eastern and southern Africa (appendix p 53). We found similar results for any breastfeeding at age 1 year, except for in Latin America and the Caribbean, where levels increased over the period of analysis.

**Table 3:** Annual changes in the rates of any breastfeeding at age 6 months and 1 year, according to country income group

	**AAAC (95% CI)**	**p value**	**Number of countries**
**Any breastfeeding at age 6 months**			
All countries	0·05 (−0·22 to 0·32)	0·72	110
High income	1·29 (1·12 to 1·45)	<0·0001	21
Upper-middle income	0·59 (−0·12 to 1·31)	0·10	27
Lower-middle income	−0·01 (−0·44 to 0·43)	0·98	32
Low income	−0·07 (−0·11 to −0·03)	<0·0001	30
**Any breastfeeding at age 1 year**			
All countries	−0·01 (−0·27 to 0·25)	0·93	105
High income	1·14 (0·99 to 1·28)	<0·0001	14
Upper-middle income	0·53 (0·23 to 0·82)	<0·0001	29
Lower-middle income	−0·10 (−0·65 to 0·45)	0·72	32
Low income	−0·13 (−0·18 to −0·09)	<0·0001	30

AAAC=absolute average annual change in percentage points.

**Figure 2:**
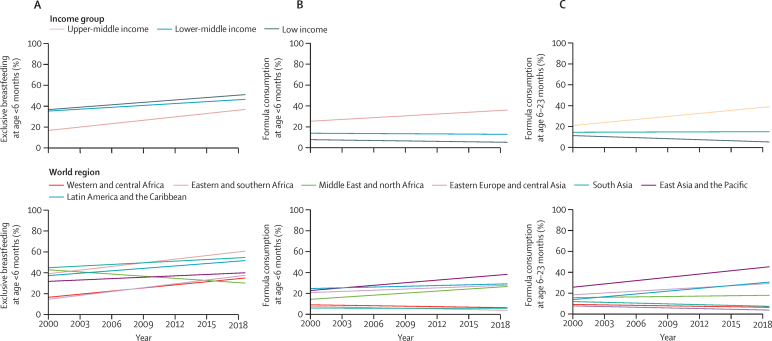
Evolution of the rates of exclusive breastfeeding and consumption of formula in low-income and middle-income countries, by age, country income group, and world region, 2000–19 (A) Exclusive breastfeeding in children younger than 6 months (83 countries, based on 344 data points), (B) consumption of formula in children younger than 6 months (83 countries, based on 328 data points), and (C) consumption of formula at age 6–23 months (83 countries, based on 328 data points).

In the pooled analysis of low-income and middle-income countries, exclusive breastfeeding in the first 6 months increased from 35·4% (95% CI 27·4–43·3) in 2000 to 48·6% (41·9–55·2) in 2019. The increase was particularly marked in upper-middle-income countries, where the rate more than doubled from less than 17% in 2000 to nearly 37% in 2019 (figure 2; table 2). In low-income and lower-middle-income countries, rates increased by just over 10 PPs in the period. In eastern and southern Africa, south Asia, and Latin America and the Caribbean, exclusive breastfeeding in the first 6 months of life started at roughly 40% in 2000, reaching 50% or more in 2019 (figure 2; table 2). Increases were also seen in all regions except in the Middle East and north Africa, where a decline of about 13 PPs was observed (table 2; figure 2A).

Across all low-income and middle-income countries, exclusive breastfeeding in the first 6 months significantly increased by 0·70 PPs per year (95% CI 0·51–0·88; p<0·0001) during the analysis period (table 4). This was the only indicator showing increases in all country income groups, particularly in upper-middle-income countries, and in all world regions except for the Middle East and north Africa, which was the only region with significant declines (table 4; appendix p 54).

**Table 4:** Annual changes in the rates of infant and young child feeding indicators in LMICs, according to country income group

	**AAAC (95% CI)**	**p value**	**Number of countries**
**Exclusive breastfeeding at age <6 months**			
All LMICs	0·70 (0·51 to 0·88)	<0·0001	83
Upper-middle income	1·06 (0·18 to 1·95)	0·018	24
Lower-middle income	0·59 (0·14 to 1·05)	0·011	30
Low income	0·76 (0·41 to 1·10)	<0·0001	29
**Formula consumption in children at age <6 months**			
All LMICs	0·06 (−0·11 to 0·22)	0·51	83
Upper-middle income	0·56 (0·23 to 0·90)	<0·0001	24
Lower-middle income	−0·05 (−0·40 to 0·29)	0·76	30
Low income	−0·13 (−0·26 to 0·00)	0·054	29
**Formula consumption at age 6–23 months**			
All LMICs	0·02 (−0·18 to 0·23)	0·82	83
Upper-middle income	0·96 (0·54 to 1·37)	<0·0001	24
Lower-middle income	0·03 (−0·30 to 0·36)	0·87	30
Low income	−0·32 (−0·57 to −0·08)	0·010	29
**Animal milk consumption at age <6 months**			
All LMICs	−0·41 (−0·51 to −0·31)	<0·0001	83
Upper-middle income	−0·91 (−1·24 to −0·57)	<0·0001	24
Lower-middle income	−0·53 (−0·77 to −0·28)	<0·0001	30
Low income	−0·22 (−0·38 to −0·05)	0·010	29
**Animal milk consumption at age 6–23 months**			
All LMICs	−0·17 (−0·39 to 0·04)	0·12	83
Upper-middle income	−1·39 (−2·06 to −0·72)	<0·0001	24
Lower-middle income	−0·35 (−0·86 to 0·16)	0·18	30
Low income	0·08 (−0·26 to 0·42)	0·65	29

AAAC=Absolute average annual change in percentage points. LMIC=low-income and middle-income country.

The rate of formula milk consumption indicators did not increase significantly for low-income and middle-income countries as a whole (table 2; figure 2B, C). However, we found a significant increase of 0·56 PPs per year (95% CI 0·23–0·90; p=0·0010) in upper-middle-income countries for formula milk consumption up to 6 months of age, and an increase of 0·96 PPs per year (0·54–1·37; p<0·0001) for consumption by children aged 6–23 months (table 4). Opposite trends were observed in both indicators for low-income countries, particularly for formula milk consumption at age 6–23 months. When analysing the world regions, east Asia and the Pacific presented the highest rates of formula milk consumption in 2019, with Latin America and the Caribbean and eastern Europe and central Asia also showing large increases (figure 2; table 2). The increase in formula milk consumption in the Middle East and north Africa was greatest among children younger than 6 months (figure 2; table 2). Consumption of formula milk persistently remained low through all the years investigated in the remaining regions, at 15% or less (table 2; figure 2B, C).

Annual changes in formula milk consumption for all age ranges were not significant for low-income and middle-income countries as a whole (table 4), but upper-middle-income countries showed a substantial increase, particularly among children aged 6–23 months (table 4). By contrast, formula consumption declined in low-income countries (table 4). Depending on the age range, world regions performed differently. For formula milk consumption in children younger than 6 months, only two regions presented increases—eastern Europe and Central Asia and the Middle East and north Africa (appendix p 54). Among children aged 6–23 months, Latin America and the Caribbean and east Asia and the Pacific showed annual increases, whereas eastern and southern Africa showed small declines (table 4; appendix p 54).

The consumption of animal milk by children younger than 6 months decreased by more than half in the period of analysis in low-income and middle-income countries, from 17·5% (95% CI 13·6–21·5) to 9·8% (6·1–13·5), and less markedly for children aged 6–23 months, from 36·3% (26·2–46·4) to 33% (25·1–40·9). Reductions were greatest in upper-middle-income countries (table 2; appendix p 54). All world regions showed reductions in the consumption of animal milk in children younger than 6 months, particularly eastern Europe and central Asia. For children aged 6–23 months, declines occurred in eastern and southern Africa, south Asia, and Latin America and the Caribbean, and more intensely in the Middle East and north Africa, where they decreased from 48·4% (44·1–52·7) in 2000 to 30·3% (18·0–42·6) in 2019 (table 2).

Annual reductions in animal milk consumption for children younger than 6 months were significant in all country income groups but only in upper-middle-income countries for children aged 6–23 months (table 4). Reductions were observed in most world regions for infants younger than 6 months, but only in the Middle East and north Africa and south Asia for children aged 6–23 months. In children aged 6–23 months, animal milk consumption increased in west and central Africa (appendix p 54).

## Discussion

As far as we are aware, this is the first report on trends in various child feeding indicators including high-income countries and low-income and middle-income countries. Given the available data, comparisons among the groups of countries were only possible for the rates of any breastfeeding at age 6 months and 1 year. When all countries with data were pooled together, the rate of any breastfeeding was constant from 2000 to 2019, at just under 90% for children younger than 6 months and around 80% at age 1 year. However, we found increases in high-income and upper-middle-income countries, almost no change in lower-middle-income countries, and reductions in low-income countries. Nevertheless, in low-income countries, approximately nine in ten children aged 1 year were breastfed in 2019, compared with only approximately four in ten in high-income countries.

Because the available indicators and societal contexts are different in high-income countries and low-income and middle-income countries, we discuss the findings from these two groups of countries separately. A publication on breastfeeding trends in 51 high-income countries showed that breastfeeding practices improved in most such countries.^17^ Progress towards improving breastfeeding practices is dependent upon broad measures at the societal level,^23^ and the positive trends in high-income countries were probably due to national efforts to improve, support, promote, and increase breastfeeding by addressing social and cultural barriers.^13^, ^24^ For example, in the USA, policies were introduced in the past decade to allow women to breastfeed in public and to be allowed to express breastmilk during working hours.^13^ In the UK, breastfeeding peer-support services were associated with increased breastfeeding initiation and duration among mothers younger than 25 years.^24^ Paid post-partum leave to both parents has been positively associated with increased breastfeeding duration in Sweden.^25^ Yet, the amount of maternity leave that mothers take varies markedly across high-income countries, from 12–14 weeks in the USA, Germany, and Switzerland to more than 30 weeks in Norway, Ireland, and the UK.^26^ By contrast, only a few countries provide paid paternity leave, such as Australia and the UK.^26^ Additional investments and policy changes are needed to promote further increases in breastfeeding in high-income societies.^27^

Our analysis of trends in exclusive breastfeeding for infants younger than 6 months were limited to low-income and middle-income countries with standardised surveys. An improvement was observed from rates of approximately 35% in 2000 to approximately 49% in 2019, thus meaning that the World Health Assembly's initial target of 50% by 2025 will probably be reached, as long as this trend does not reverse.^10^ However, the annual increase of 0·70 PPs per year will fall short of achieving the revised goal of 70% by 2030^10^ because at the present rate only approximately 61% of children will be exclusively breastfed by 2030. Our results are consistent with an earlier analysis showing increases in exclusive breastfeeding rates in low-income and middle-income countries up to 2018.^9^ Progress, however, has not been uniform. Although low-income and lower-middle-income countries are already close to the target, upper-middle-income countries lag behind with a rate of 37% in 2019.

The observed increase in exclusive breastfeeding over the past decades is consistent with efforts globally to protect, promote, and support this practice,^9^ including implementation of the Code of Marketing of Breastmilk Substitutes (the Code) and interventions within health services and communities, such as the Baby-Friendly Hospital Initiative, counselling to mothers and families on breastfeeding exclusivity and continuation, and increases in the paid maternity leave period.^9^, ^28^ The experience of several Latin American and Caribbean and African countries shows that political will, community-level interventions (eg, the *Amamenta e Alimenta* strategy in Brazil), legislation and policies (eg, in Ghana and Madagascar), breastfeeding promotion, advocacy, and research, as well as changes in demographic characteristics have effectively improved breastfeeding rates in recent decades.^11^, ^12^, ^14^ Yet, much more needs to be achieved regarding maternity protection, given that of 163 countries with information, only 34% met the International Labour Organization standards on length of paid maternity leave (at least 14 weeks at two-thirds of earnings, with at least two-thirds paid by government support).^26^ In the context of low-income and middle-income countries, another important limitation is that maternity leave legislation does not cover most women who work in the informal labour market.^23^ According to the World Breastfeeding Trends Initiative,^29^ among 84 countries assessed, most scored under seven points on a scale of ten indicators on policy and programmes to promote and protect breastfeeding practices at a national level.

In low-income and middle-income countries, breastfeeding promotion strategies have been focused more intensively on young infants, for whom exclusive breastfeeding confers important protection against mortality due to infectious diseases.^3^ By contrast, efforts to promote continued breastfeeding up to age 2 years are less visible. Evidence concerning the positive effects of longer breastfeeding duration on improving cognitive development, reducing the risk of overweight and diabetes, and preventing breast and ovarian cancer among women who are nursing supports the need for further investment in continued breastfeeding up to the second year of life and beyond.^3^, ^30^ Additionally, research has shown the economic benefits of exclusive and continued breastfeeding through national health savings and improved intelligence and income in adulthood, both for low-income and middle-income countries and high-income countries.^3^, ^31^ Breastfeeding support throughout the first and second year requires workplace protection through regular breaks for women who are nursing, with appropriate rooms to express their milk, or day-care centres close to the workplace where women can breastfeed their children.^23^ Additionally, health-care workers, communities, and families should be made aware of the benefits of prolonged breastfeeding in order to support mothers.^23^

Only low-income countries are close to 100% breastfeeding at age 12 months, which might be attributed to long-standing cultural practices rather than to breastfeeding promotion activities.^23^ In these countries, we observed small but significant reductions over the years of analysis, potentially due to increasing urbanisation and adoption of Western lifestyles, given that family wealth is inversely associated with breastfeeding duration in low-income and middle-income countries.^6^ According to the 2016 *Lancet* Breastfeeding Series, the overall drop in continued breastfeeding at age 12–15 months in low-income and middle-income countries was partially due to declines among the poorest 20% of people in each country.^3^

Standardised survey data also allowed the study of formula and other types of milk consumption in low-income and middle-income countries. Formula milk consumption showed an alarming increase in upper-middle-income countries, particularly among children older than 6 months. Since the mid-20th century, commercial breastmilk substitutes have been available in most high-income countries. In the 1980s, the Code was developed to control the marketing of formula milk targeted at infants up to age 6 months.^32^ As of 2018, 76 countries extended the restriction to also cover the marketing of breastmilk substitutes for children older than 1 year, but only 31 included restrictions to products up to age 36 months,^33^ which our analysis suggests is the group in which formula milk consumption increased more rapidly. Strategies are constantly being adapted and innovated by the formula industry, including the development of products aimed at older infants and toddlers, as well as the expansion of its markets across the globe.^15^ Strategies include marketing through health facilities, digital and social media, and labelling and point-of-sale advertising, as well as among paediatrician associations.^33^, ^34^ Total formula milk sales increased from 3·5 kg/child to 7·4 kg/child in 2005–19, led mainly by highly populated upper-middle-income countries,^15^ which is consistent with our current findings. The marketing and promotion of breastmilk substitutes undermines optimal breastfeeding practices^16^ and reinforces the need to fully enact the Code into national law and strengthen its enforcement via rigorous monitoring and financial sanctions, including tracking marketing via digital and social media.

The paradoxical combination of increases in exclusive breastfeeding and in formula milk consumption from birth to age 23 months in upper-middle-income countries is explained by sharp declines in children who consume animal milk, a finding that was also observed to a lesser extent in lower-middle-income countries. As far as we are aware, this is the first multi-country study on trends in the consumption of animal milk. Growing affluence in middle-income countries is likely to be behind the replacement of animal milk by formula.

Our analysis by world regions showed some important patterns. All regions showed increasing trends in exclusive breastfeeding, with one exception: the Middle East and north Africa. Downturns or stable trends have been described for some highly populated countries in the region, such as Egypt, Yemen, and Jordan, corroborating our findings.^35^ Research from the region suggests that the drivers of these trends include the poor implementation of the Baby-Friendly Hospital Initiative and of the Code, allowing formula companies to expand their markets,^15^ as well as the high rate of prelacteal feeds (mostly water-based), which negatively affect breastfeeding practices.^8^, ^35^, ^36^ Breastfeeding at age 1 year showed rates above 80% in three regions and remained constant in west and central Africa but declined slightly in eastern and southern Africa and sharply in South Asia. Two regions (the Middle East and north Africa and east Asia and the Pacific) with baseline rates close to 80% showed declines over time, and another two (Latin America and the Caribbean and eastern Europe and central Asia) started at approximately 60% and increased over time. North America and western Europe started below 20% and showed considerable increases. Consumption of formula milk in the first 6 months increased in all regions, except for in the two sub-Saharan African regions. Our results by world region and by country (appendix pp 53–54) are relevant for monitoring progress and designing policies and programmes.^23^

In 2019, UNICEF published an analysis of global trends in exclusive breastfeeding from 2005 to 2018 covering 80 low-income and middle-income countries.^9^ Their results are consistent with our own findings, except for the decline in the Middle East and north Africa region, which was not observed in their analysis, probably because of the number of countries included and the longer period of our analysis. Our study advances the analyses of global trends by also including high-income countries, by analysing data from 113 countries and 487 surveys carried out since the 1990s, and by analysing continued breastfeeding, formula, and animal milk consumption.

The limitations of our work include the small number of surveys for some regions of the world, such as east Asia and the Pacific and the Middle East and north Africa. The same limitation affected the 2019 UNICEF trend estimates. This gap highlights the need for more countries to routinely do standardised national surveys. Another limitation is the lack of trend data for highly populated countries, such as Mexico, Russia, and China, with China accounting for a third of world formula milk sales in 2018.^15^ Data for North America were restricted to the USA because no trend data were available for Canada. Although more recent DHS data for the Philippines were available (2013 and 2017), the data required for estimating exclusive breastfeeding and formula feeding are not presented or collected in the databases. Additionally, because data on some feeding indicators were not available, we could not calculate pooled trends for exclusive breastfeeding, formula, and animal milk consumption for high-income countries, showing the urgent need for standardised data for these countries. Estimates for high-income countries were obtained from national surveys or from nationally representative health services data based on 24-h hour food recall. For European countries, we checked whether the data were part of the European Health Information Gateway from WHO Europe. More detailed analyses of factors that might affect exclusive breastfeeding, such as area of residence (urban or rural) or maternal reproductive or nutritional factors, were out of the scope of the present descriptive analyses.

Over a 20-year period, optimal breastfeeding practices have been promoted worldwide, resulting in increases in breastfeeding, particularly in exclusive breastfeeding and in breastfeeding at age 6 months and 12 months in high-income countries and upper-middle-income countries. However, declines in breastfeeding indicators in low-income countries and in the Middle East and north Africa region deserve attention. The previous global target^10^ of 50% of children younger than 6 months exclusively breastfed by 2025 is likely to be achievable sooner than previously planned, yet the target of 70% by 2030 remains a great challenge. Despite the positive results, the increase in formula milk consumption by children in emerging economies—especially formula for toddlers—reflects the evolution, expansion, and promotion of milk formula supply chains. Countries that have been successful in improving feeding patterns relied upon multiple interventions that need to work in synchrony,^37^ including implementation of the Code, breastfeeding counselling at the individual-level and community-level, maternity leave legislation, and workplace protection to working women. These policy changes were accompanied by policies targeting hospitals and clinical facilities, health worker training, mass media campaigns, and community-based interventions.^23^, ^37^ Continued monitoring of feeding indicators is essential for assessing progress and learning lessons from successful country experiences. DHS and MICS data provide the main source for UNICEF and WHO estimates of infant and young child feeding practices across low-income and middle-income countries. In order to ensure high-quality data for these estimates, such surveys need be carried out regularly in low-income and middle-income countries and similar standardised surveys should be encouraged in high-income countries.

## Data sharing

Individual-level data used in these analyses are publicly available at the DHS and MICS websites. Information retrieved through literature review is freely available by accessing the links provided in the appendix (p 3).

## Declaration of interests

We declare no competing interests.
