# Supplementary material for: Rates and time trends in the consumption of breastmilk, formula, and animal milk by children younger than 2 years from 2000 to 2019: analysis of 113 countries
Source: Lancet Child Adolesc Health. 2021 Sep;5(9):619–30. doi: 10.1016/S2352-4642(21)00163-2 (PMC8376656; doi:10.1016/S2352-4642(21)00163-2)
Supplement: Supplementary appendix [file mmc1.pdf]

THE LANCET
Child & Adolescent Health

Supplementary appendix

This appendix formed part of the original submission and has been peer reviewed. We post it as supplied by the authors.

Supplement to: Neves PAR, Vaz JS, Maia FS, et al. Rates and time trends in the consumption of breastmilk, formula, and animal milk by children younger than 2 years from 2000 to 2019: analysis of 113 countries. *Lancet Child Adolesc Health* 2021; published online July 7. http://dx.doi.org/10.1016/S2352-4642(21)00163-2.

Appendix 1. Literature search strategy

Major scientific databases (published peer-reviewed literature)

The following search strategy was used in PubMed[1]:

(Andorra OR Antigua and Barbuda OR Argentina OR Aruba OR Australia OR Austria OR Bahamas OR Bahrain OR Barbados OR Belgium OR Bermuda OR "British Virgin Islands" OR "Brunei Darussalam" OR Canada OR "Cayman Islands" OR " Channel Islands" OR Chile OR Curacao OR Cyprus OR "Czech Republic" OR Denmark OR England OR Estonia OR "Faroe Islands" OR Finland OR France OR "French Polynesia" OR Germany OR Gibraltar OR Greece OR Greenland OR Guam OR "Hong Kong" OR Hungary OR Iceland OR Ireland OR "Isle of Man" OR Israel OR Italy OR Japan OR Korea OR Kuwait OR Latvia OR Liechtenstein OR Lithuania OR Luxembourg OR "Macao" OR Malta OR Monaco OR Netherlands OR "New Caledonia" OR "New Zealand" OR "Northern Mariana Islands" OR Norway OR Oman OR Palao OR Poland OR Portugal OR "Puerto Rico" OR Qatar OR "San Marino" OR "Saudi Arabia" OR Scotland OR Seychelles OR Singapore OR "Sint Maarten" OR "Slovak Republic" OR Slovenia OR Spain OR "Saint Kitts and Nevis" OR "St Martin" OR Sweden OR Switzerland OR "Trinidad and Tobago" "Turks and Caicos Islands" OR "United Arab Emirates" OR "United Kingdom" OR "United States" OR Uruguay OR "Virgin Islands")

AND

(breastfeeding) OR (breast feeding) OR (breastfeeding practices)OR (breastfed) OR (breastfeed) OR (infant feeding) OR (infant feeding practices)

AND

(National Survey) OR (National Health) OR (Nutrition Survey) OR(Nutritional Survey) OR (Nutritional Surveys) OR (Health Survey) OR(Surveys Health)A total of 2,806 references were identified as a result of this search.

Grey literature

The following search strategy was used in Google:

Country name + ((breastfeeding) OR (breast feeding) OR(breastfeeding practices) OR (breastfed) OR (breastfeed) OR (infant feeding) OR (infant feeding practices)) + site:.gov

Country name + ((breastfeeding) OR (breast feeding) OR(breastfeeding practices) OR (breastfed) OR (breastfeed) OR (infant feeding) OR (infant feeding practices)) + file:.pdfCountry name + ((breastfeeding) OR (breast feeding) OR(breastfeeding practices) OR (breastfed) OR (breastfeed) OR (infant feeding) OR (infant feeding practices)) + file:.xls

Country name + allintitle:((breastfeeding) OR (breast feeding) OR(breastfeeding practices) OR (breastfed) OR (breastfeed) OR (infant feeding) OR (infant feeding practices))

Country name + allintext:((breastfeeding) OR (breast feeding) OR(breastfeeding practices) OR (breastfed) OR (breastfeed) OR (infant feeding) OR (infant feeding practices))

Other grey literature searches are also being reviewed:

- Ministries of health of all countries
- WORLDCAT: www.worldcat.org ✔STATISTICS OFFICES: https://globaledge.msu.edu/globalresources/resourcesbytag/statistics-office
- PACIFIC ISLAND COUNTRIES AND TERRITORIES: http://prism.spc.int/component/finder/search?q=health&Itemid=262
- COUNTRY PLANNING CYCLE: http://www.nationalplanningcycles.org/file-repository/CAF

[1]Available from: www.pubmed.com.

Appendix 2. Country and data source of breastfeeding indicator data for high-income and some upper-middle income countries.

Country	Data source
Argentina	Ministerio de Salud de la Nación. Dirección Nacional de Maternidad, Infancia y Adolescencia. Situación de la lactancia materna en Argentina. Informe 2018. Access: https://bancos.salud.gob.ar/sites/default/files/2018-10/0000001281cnt-situacion-lactancia-materna-2018.pdf
Bahrain	Bahrain Nutrition Profile – Nutrition and Consumer Protection Division, FAO, 2007. Access: http://www.fao.org/publications/card/en/c/22dea75a-0156-4341-9f5d-f2a3797c3317/
Brazil	Boccolini CS,Boccolini PMM, Monteiro FR, Venâncio SI, Giugliani ERJ. Tendência de indicadores do aleitamento materno no Brasil em três décadas. Rev Saude Publica. 2017;51:108. Access: https://doi.org/10.11606/S1518-8787.2017051000029
Czech Republic	Vydáno za podpory Ministerstva zdravotnictví ČR. Výživa novorozenců a kojenců do jednoho roku v České republice v letech 2004 – 2017. Access: http://www.kojeni.cz/kojeni-v-cr/vyziva-novorozencu-a-kojencu-do-jednoho-roku-v-cr-v-letech-2004-2017/
Denmark	Den Nationale Børnedatabase. Amning og udsættelse for tobaksrøg i første leveår. Access: https://www.esundhed.dk/Registre/Den-nationale-boernedatabase/Foerste-leveaar
Estonia	ANDMEBAAS. Health Statistics and Health Research Database. SR91. https://statistika.tai.ee/pxweb/en/Andmebaas/Andmebaas__01Rahvastik__02Synnid/SR91.px/
Finland	Uusitalo L, Nyberg H, Pelkonen M, Sarlio-Lähteenkorva S, Hakulinen-Viitanen T, Virtanen S (2012). Imeväisikäisten ruokinta Suomessa vuonna 2010. Access: http://urn.fi/URN:ISBN:978-952-245-598-7
Greece	Iliodromiti Z, Zografaki I, Papamichail D, Stavrou T, Gaki E, Ekizoglou C, et al. Increase of breast-feeding in the past decade in Greece, but still low uptake: cross-sectional studies in 2007 and 2017. Pub Health Nutr. 2020;23(6):961–70. Access: https://doi.org/10.1179/2046905514Y.0000000134
Korea	Republic: Analysis on Breastfeeding and Nutritional Health Among Children and Adolescents and Counter Policies in Korea (2018). Access: https://www.kihasa.re.kr/common/filedown.do?seq=14977
Kuwait	Al-Taiar A, Alqaoud N, Hammoud MS, Alanezi F, Aldalmani N, Subhakaran M. WHO infant and young child feeding indicators in relation to anthropometric measurements. Pub Health Nutr. 2020;23(10):1665–76. Access: https://doi.org/10.1017/S1368980019004634
Latvia	Veselības statistikas datubāze. Access: https://statistika.spkc.gov.lv/pxweb/en/Health/Health__Mates_berna_veseliba/MCH100_kruts_barosana.px/
Luxembourg	DesrochesS, Brochmann C, Wagener Y, Lehners S (2017). Enquête nationale sur l'alimentation des enfants de 4, 6 et 12 mois au Grand-Duché de Luxembourg en 2015. Access: https://sante.public.lu/fr/publications/e/etude-alba-2015-alimentation-bebes/index.html
Netherlands	Peeters D, Lanting CI, JP van Wouwe. Peiling melkvoeding van zuigelingen 2015. Access: https://www.tno.nl/media/5249/infoblad-peiling-melkvoeding-van-zuigelingen-2015.pdf
Norway	Myhre JB, Andersen LF, Kristiansen AL. "Spedkost 3. Landsomfattende undersøkelse av kostholdet blant spedbarn i Norge, 6 måneder" [Spedkost 3. Nationwide dietary survey among infants in Norway, age 6 months]. Rapport 2020. Oslo: Folkehelseinstituttet og Universitetet i Oslo, 2020. Access: https://www.fhi.no/publ/2020/spedkost-3/
Oman	Oman National Nutrition Survey (2017). Access: https://groundworkhealth.org/wp-content/uploads/2020/04/ONNS_Report_2017.pdf
Portugal	Kislaya I, Braz P, Dias CM, Loureiro I (2017). A evolução do aleitamento materno em Portugal nas últimas duas décadas: dados dos Inquéritos Nacionais de Saúde. http://www.insa.min-saude.pt/a-evolucao-do-aleitamento-materno-em-portugal-nas-ultimas-duas-decadas-dados-dos-inqueritos-nacionais-de-saude/
Singapore	National Breastfeeding Survey (2011). Access: https://data.gov.sg/dataset/national-breastfeeding-survey?view_id=2e442470-4bd0-4f12-be31-19419aed86ef&resource_id=af4e8981-0340-471e-a503-e8f7013b0424
Spain	Encuesta Nacional de Salud España (ENSE) 2017. Ministerio de Sanidad, Consumo y Bienestar Social. Access: https://www.mscbs.gob.es/estadEstudios/estadisticas/encuestaNacional/encuestaNac2017/ENSE17_pres_web.pdf
Sweden	Statistik om amming 2016. Statistikdatabas. Socialstyrelsen, publiceringsar 2018 [Statistics on Breastfeeding 2016.]. Access: http://www.socialstyrelsen.se/statistik/statistikdatabas/amning
Switzerland	Dratva J, Gross K, Späth A, Stutz EZ. SWIFS – Swiss Infant Feeding Study. A national study on infant feeding and health in the child's first year. Swiss Tropical and Public Health Institute. Basel, December 2014. Access: https://www.swisstph.ch/fileadmin/user_upload/SwissTPH/Projects/SWIFS/ExecutiveSummary_SWIFS_EN.pdf
United Kingdom	Infant Feeding Survey – UK, 2010. Official statistics, National statistics, Survey. Access: https://digital.nhs.uk/data-and-information/publications/statistical/infant-feeding-survey/infant-feeding-survey-uk-2010
United States	Ruowei Li Cria G. Perrine, Jian Chen. Estimation of Breastfeeding Rates in the United States from the National Immunization Survey: Effect of a Sampling Change from Landline and Cellular Telephones to Cellular Telephones Only. Full report, 2019. Division of Nutrition, Physical Activity, and Obesity, National Center for Chronic Disease Prevention and Health Promotion, Centers for Disease Control and Prevention, Atlanta Georgia. Access: https://www.cdc.gov/breastfeeding/pdf/Estimation-Breastfeeding-Rates-US-NIS.pdf
Uruguay	Bove, María Isabel. Encuesta nacional sobre estado nutricional, prácticas de alimentación y anemia en niños menores de dos años, usuarios de servicios de salud de los subsectores público y privado del Uruguay / María Isabel Bove, Florencia Cerruti. – Montevideo: UNICEF: MSP: MIDES: RUANDI, 2011. Access: http://repositorio.mides.gub.uy:8080/xmlui/handle/123456789/236

Supplementary figure 1. World map showing the 113 countries included in the trend analyses of infant and young child feeding indicators.

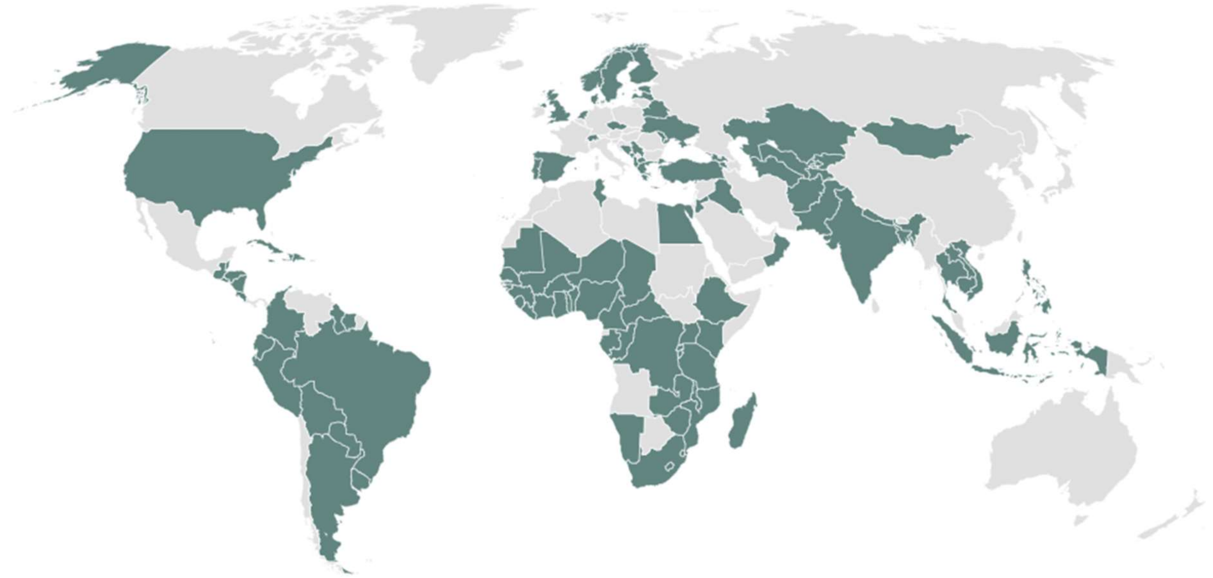

Supplementary figure 2. Flow-diagram of selection of the high income countries for the trend analysis.

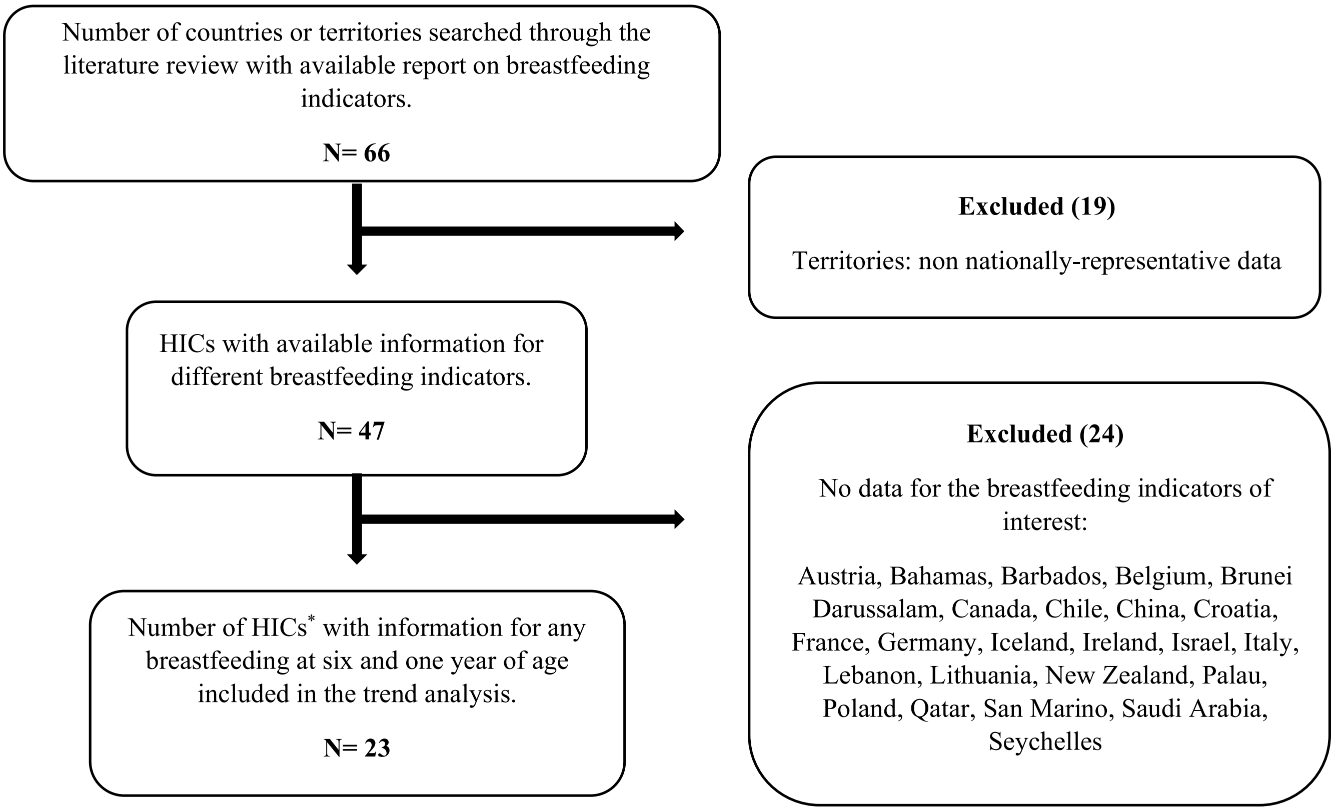

*Although Argentina and Brazil were classified as upper-middle income countries in 2010, data for these countries were retrieved from the literature search.

Supplementary figure 3. Flow-diagram of selection of countries for the trend analysis of any of breastfeeding at 6 months and 1 year globally.

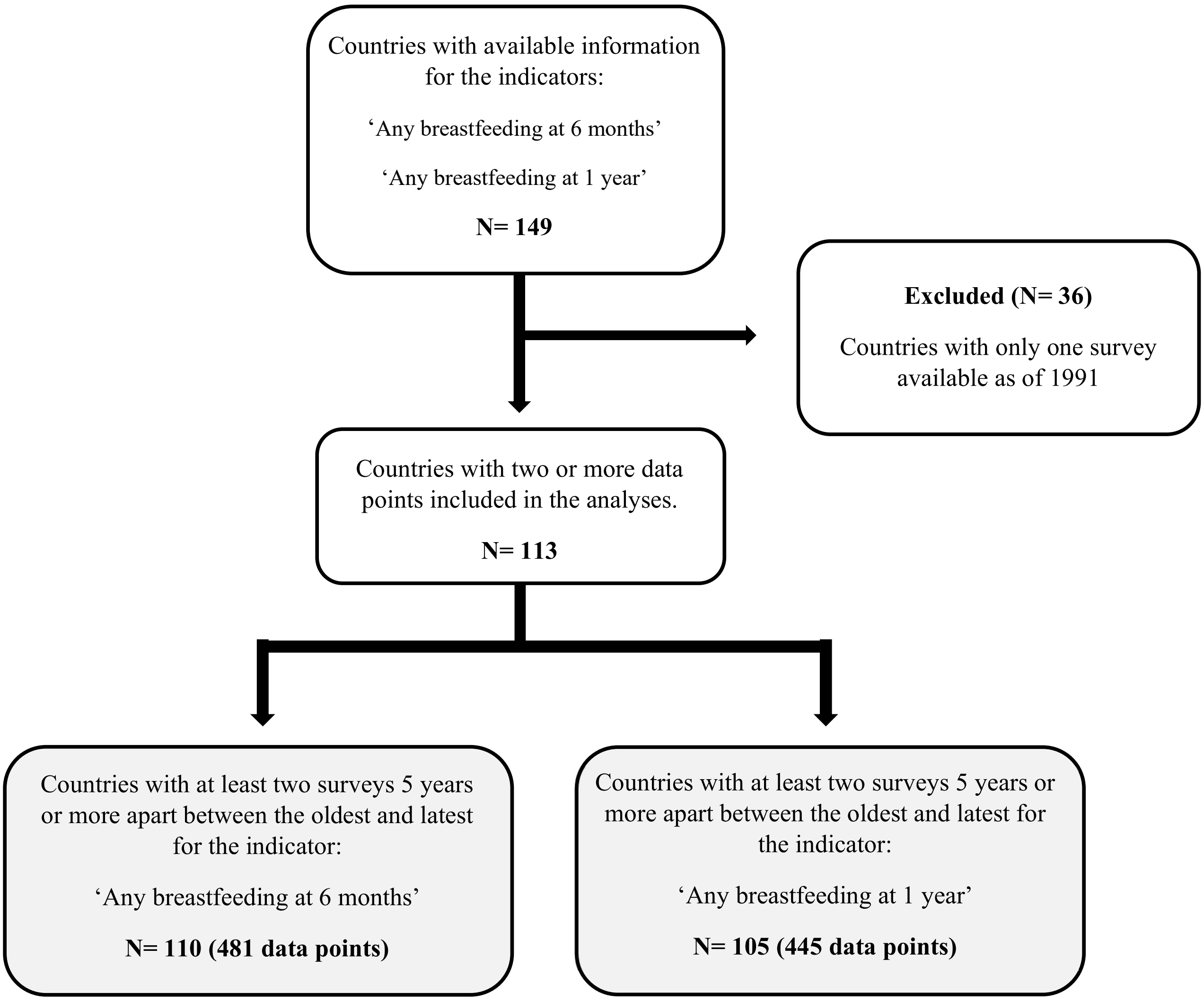

Supplementary figure 4. Flow-diagram of selection of countries for the trend analysis of infant and young child feeding indicators restricted to low- and middle-income countries.

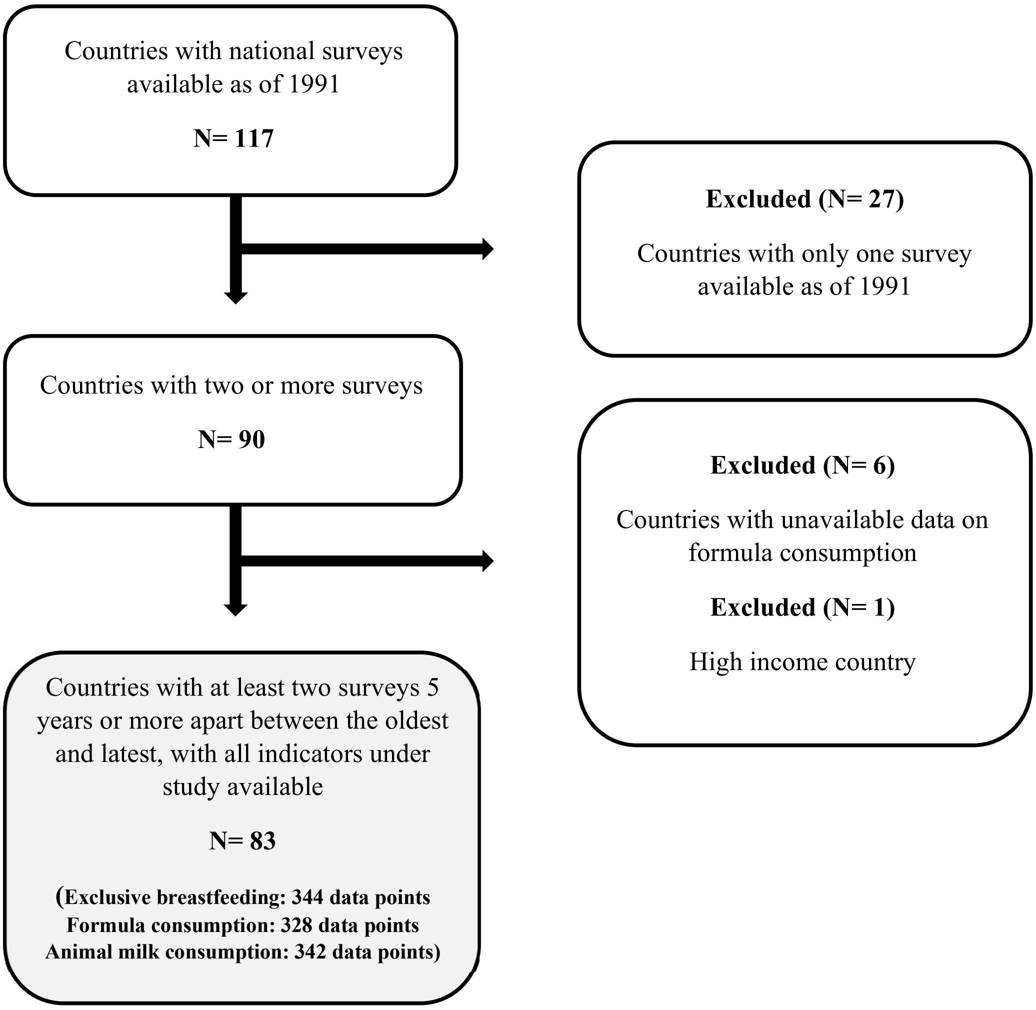

Supplementary figure 5. Evolution of the prevalence of animal milk consumption under 6 months (A) and between 6-23 months (B) by country income groups and regions of the world throughout the period of analysis.

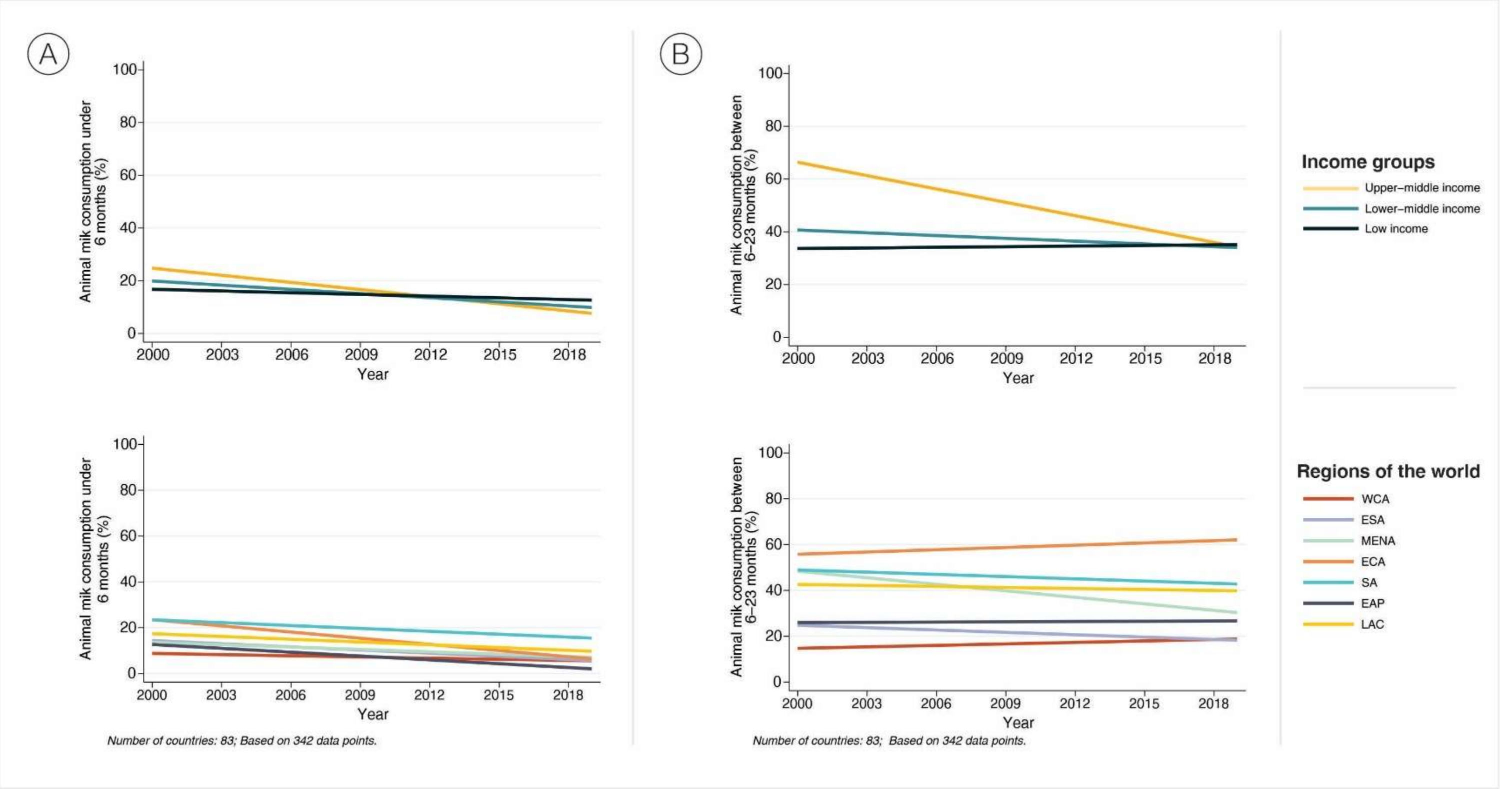

WCA: Western and Central Africa, ESA: Eastern and Southern Africa, MENA: Middle East and North Africa, ECA: Eastern Europe and Central Asia, SA: South Asia, EAP: East Asia and Pacific, LAC: Latin America and the Caribbean.

Supplementary table 1. Countries, surveys, prevalence, and number of children included in the trend analysis of any breastfeeding indicators globally. Source: Demographic Health Survey (DHS), Multiple Indicator Cluster Survey (MICS), Reproductive and Health Survey (RHS), Literature review (LIT).

ISO	Country	Survey and year	Income group*	Region of the world**	Any breastfeeding at six months			Number of children	Any breastfeeding at one year			Number of children
					Prevalence	95% CI			Prevalence	95% CI		
AFG	Afghanistan	MICS 2010	LI	SA	93.5	91.3	95.1	993	88.2	85.1	90.8	715
AFG	Afghanistan	DHS 2015	LI	SA	94.6	92.6	96.1	2241	80.3	76.4	83.6	1773
ALB	Albania	RHS 2002	LMI	ECA	77.6	66.8	85.6	127	64.8	52.4	75.5	144
ALB	Albania	MICS 2005	LMI	ECA	89.1	77.8	95.0	64	65.8	52.6	77.0	67
ALB	Albania	DHS 2008	LMI	ECA	86.2	75.2	92.8	92	72.3	56.8	83.8	86
ALB	Albania	DHS 2017	UMI	ECA	85.3	76.9	91.0	173	70.1	59.7	78.7	198
ARG	Argentina	MICS 2011	UMI	LAC	76.1	69.8	81.5	585	62.0	55.5	68.0	620
ARG	Argentina	LIT 2015	UMI	LAC	39.0	NA	NA	9808	71.0	NA	NA	2731
ARG	Argentina	LIT 2017	HIC	LAC	36.0	34.3	37.9	2773	78.0	76.0	79.0	3243
ARM	Armenia	DHS 2000	LI	ECA	73.5	63.0	81.8	98	35.3	24.9	47.3	105
ARM	Armenia	DHS 2005	LMI	ECA	72.7	57.1	84.2	96	48.6	34.8	62.7	91
ARM	Armenia	DHS 2010	LMI	ECA	75.5	60.6	86.0	101	52.3	43.3	61.3	119
ARM	Armenia	DHS 2015	LMI	ECA	74.9	63.8	83.5	136	54.2	42.9	65.0	115
BHR	Bahrain	LIT 1995	UMI	MENA	***	***	***	***	64.0	NA	NA	1947
BHR	Bahrain	LIT 2002	HIC	MENA	***	***	***	***	65.3	NA	NA	408
BGD	Bangladesh	DHS 1993	LI	SA	99.5	97.9	99.9	379	96.4	93.8	97.9	422
BGD	Bangladesh	DHS 1996	LI	SA	98.6	96.6	99.4	353	97.5	95.4	98.6	408
BGD	Bangladesh	DHS 1999	LI	SA	98.5	96.2	99.4	371	96.6	93.8	98.2	456
BGD	Bangladesh	DHS 2004	LI	SA	98.4	96.3	99.4	475	96.9	94.5	98.3	420
BGD	Bangladesh	MICS 2006	LI	SA	97.0	95.8	97.8	2048	96.3	95.1	97.2	2039
BGD	Bangladesh	DHS 2007	LI	SA	99.8	99.0	99.9	442	95.0	91.2	97.2	308
BGD	Bangladesh	DHS 2011	LI	SA	97.3	95.2	98.5	519	95.4	92.8	97.1	600
BGD	Bangladesh	MICS 2012	LI	SA	97.0	95.6	98.0	1361	94.5	92.8	95.8	1427
BGD	Bangladesh	DHS 2014	LMI	SA	98.2	96.1	99.2	491	95.7	92.9	97.4	575
BGD	Bangladesh	MICS 2019	LMI	SA	97.1	96.0	97.9	1699	94.7	93.2	95.9	1418

ISO	Country	Survey and year	Income group*	Region of the world**	Any breastfeeding at six months			Number of children	Any breastfeeding at one year			Number of children
					Prevalence	95% CI			Prevalence	95% CI		
BLR	Belarus	MICS 2005	LMI	ECA	49.1	40.1	58.1	194	20.1	15.3	26.0	222
BLR	Belarus	MICS 2012	UMI	ECA	50.4	42.3	58.4	245	32.0	24.7	40.2	235
BLZ	Belize	RHS 1991	LMI	LAC	63.1	51.4	73.4	163	56.4	46.6	65.8	143
BLZ	Belize	MICS 2006	UMI	LAC	66.4	51.2	78.9	64	52.0	40.2	63.6	44
BLZ	Belize	MICS 2011	LMI	LAC	67.1	57.6	75.3	104	63.7	54.3	72.3	129
BLZ	Belize	MICS 2015	UMI	LAC	82.2	70.2	90.1	109	59.8	50.5	68.4	195
BEN	Benin	DHS 1996	LI	WCA	99.2	97.4	99.8	344	98.7	96.3	99.5	320
BEN	Benin	DHS 2001	LI	WCA	99.4	97.7	99.9	385	99.0	96.8	99.7	322
BEN	Benin	DHS 2006	LI	WCA	98.9	98.1	99.4	1165	97.8	96.7	98.6	1112
BEN	Benin	DHS 2011	LI	WCA	91.2	89.1	92.9	914	85.6	82.8	88.0	800
BEN	Benin	MICS 2014	LI	WCA	98.7	97.6	99.3	916	97.9	96.2	98.8	772
BEN	Benin	DHS 2017	LI	WCA	97.2	95.8	98.1	939	94.2	92.1	95.7	851
BOL	Bolivia	DHS 1994	LMI	LAC	90.1	86.0	93.1	417	80.0	74.8	84.3	381
BOL	Bolivia	DHS 1998	LMI	LAC	95.6	92.3	97.5	476	83.2	78.3	87.1	467
BOL	Bolivia	DHS 2003	LMI	LAC	93.9	90.5	96.1	685	90.1	86.9	92.5	610
BOL	Bolivia	DHS 2008	LMI	LAC	95.6	93.3	97.2	559	88.1	84.2	91.2	548
BIH	Bosnia and Herzegovina	MICS 2006	LMI	ECA	53.0	42.2	63.6	194	34.4	26.2	43.7	228
BIH	Bosnia and Herzegovina	MICS 2011	UMI	ECA	63.6	45.2	78.7	78	23.9	14.4	36.9	124
BRA	Brazil	DHS 1996	UMI	LAC	60.5	53.9	66.8	313	39.7	34.2	45.5	362
BRA	Brazil	DHS 2006	UMI	LAC	77.3	67.3	84.9	154	50.9	36.4	65.2	130
BRA	Brazil	LIT 2013	UMI	LAC	***	***	***	***	45.4	NA	NA	NA
BFA	Burkina Faso	DHS 1998	LI	WCA	99.9	99.0	100.0	392	99.5	97.9	99.9	363
BFA	Burkina Faso	DHS 2003	LI	WCA	100.0	-	-	736	98.9	96.9	99.6	671
BFA	Burkina Faso	MICS 2006	LI	WCA	96.4	93.1	98.2	394	98.0	95.9	99.0	435
BFA	Burkina Faso	DHS 2010	LI	WCA	98.6	97.5	99.3	1029	97.7	96.4	98.6	971
BDI	Burundi	DHS 2010	LI	ESA	98.6	97.2	99.3	517	96.4	94.4	97.8	517
BDI	Burundi	DHS 2016	LI	ESA	98.2	96.9	99.0	853	97.8	96.6	98.6	904
KHM	Cambodia	DHS 2000	LI	EAP	95.9	93.1	97.6	592	92.7	90.0	94.7	527

ISO	Country	Survey and year	Income group[*]	Region of the world[**]	Any breastfeeding at six months			Number of children	Any breastfeeding at one year			Number of children
					Prevalence	95% CI			Prevalence	95% CI		
KHM	Cambodia	DHS 2005	LI	EAP	95.2	91.3	97.4	525	93.4	90.6	95.4	566
KHM	Cambodia	DHS 2010	LI	EAP	94.1	91.0	96.2	513	89.7	86.2	92.4	569
KHM	Cambodia	DHS 2014	LI	EAP	93.3	90.6	95.3	497	86.9	82.9	90.1	460
CMR	Cameroon	DHS 1998	LI	WCA	98.3	95.4	99.4	255	90.0	84.1	93.9	220
CMR	Cameroon	DHS 2004	LI	WCA	97.6	95.7	98.6	537	89.4	86.6	91.7	520
CMR	Cameroon	MICS 2006	LMI	WCA	96.8	94.4	98.2	500	84.8	80.4	88.4	521
CMR	Cameroon	DHS 2011	LMI	WCA	96.7	95.4	97.7	799	85.5	82.2	88.3	800
CMR	Cameroon	MICS 2014	LMI	WCA	96.3	93.9	97.7	504	79.6	75.0	83.4	496
CMR	Cameroon	DHS 2018	LMI	WCA	91.5	88.6	93.7	651	73.0	68.0	77.4	591
CAF	Central Afr. Republic	DHS 1994	LI	WCA	100.0	-	-	298	97.8	96.0	98.9	313
CAF	Central Afr. Republic	MICS 2006	LI	WCA	96.9	94.9	98.2	802	90.0	86.5	92.7	666
CAF	Central Afr. Republic	MICS 2010	LI	WCA	96.6	94.5	98.0	807	90.9	87.2	93.6	741
TCD	Chad	DHS 1996	LI	WCA	99.7	98.5	99.9	545	97.1	94.7	98.4	433
TCD	Chad	DHS 2004	LI	WCA	99.2	97.3	99.7	368	95.7	92.2	97.7	327
TCD	Chad	MICS 2010	LI	WCA	95.9	94.1	97.1	1289	90.4	87.5	92.7	1028
TCD	Chad	DHS 2014	LI	WCA	96.6	95.4	97.5	1269	91.3	88.9	93.2	953
COL	Colombia	DHS 1995	LMI	LAC	76.8	71.8	81.2	355	52.7	46.7	58.5	344
COL	Colombia	DHS 2000	LMI	LAC	78.6	73.6	82.8	334	58.9	52.4	65.2	307
COL	Colombia	DHS 2005	LMI	LAC	82.0	78.5	85.1	1005	64.0	59.5	68.4	928
COL	Colombia	DHS 2010	UMI	LAC	88.5	85.8	90.6	1153	62.0	58.2	65.6	1169
COM	Comoros	DHS 1996	LI	ESA	95.6	89.3	98.3	114	90.5	84.6	94.3	147
COM	Comoros	DHS 2012	LI	ESA	95.4	90.7	97.8	258	79.4	72.3	85.1	202
COG	Congo Brazzaville	DHS 2005	LMI	WCA	96.1	92.9	97.9	379	85.8	80.4	89.9	279
COG	Congo Brazzaville	DHS 2011	LMI	WCA	93.3	89.7	95.7	721	86.2	81.2	90.1	626
COG	Congo Brazzaville	MICS 2014	LMI	WCA	95.1	92.1	97.1	705	79.0	72.6	84.2	559
COD	Congo Dem. Republic	DHS 2007	LI	WCA	97.6	95.5	98.7	625	94.7	91.8	96.6	606
COD	Congo Dem. Republic	MICS 2010	LI	WCA	98.1	96.6	98.9	842	93.2	90.6	95.1	823
COD	Congo Dem. Republic	DHS 2013	LI	WCA	97.3	95.2	98.6	1334	95.6	94.1	96.8	1212

ISO	Country	Survey and year	Income group[*]	Region of the world[**]	Any breastfeeding at six months			Number of children	Any breastfeeding at one year			Number of children
					Prevalence	95% CI			Prevalence	95% CI		
COD	Congo Dem. Republic	MICS 2017	LI	WCA	97.4	95.7	98.4	1569	92.9	90.4	94.8	1525
CRI	Costa Rica	RHS 1992	LMI	LAC	59.7	50.5	68.2	119	28.6	20.8	37.9	105
CRI	Costa Rica	MICS 2011	UMI	LAC	82.2	65.5	91.9	163	50.6	36.2	65.0	146
CIV	Cote d'Ivoire	DHS 1994	LI	WCA	99.2	97.4	99.7	460	97.3	94.7	98.6	357
CIV	Cote d'Ivoire	DHS 1998	LI	WCA	96.6	85.3	99.3	136	97.3	94.6	98.7	141
CIV	Cote d'Ivoire	MICS 2006	LI	WCA	97.2	94.5	98.6	703	89.2	85.5	92.0	623
CIV	Cote d'Ivoire	DHS 2011	LMI	WCA	95.9	92.8	97.7	532	92.1	88.3	94.7	492
CIV	Cote d'Ivoire	MICS 2016	LMI	WCA	98.0	95.8	99.0	655	93.1	89.4	95.6	630
CUB	Cuba	MICS 2006	LMI	LAC	63.6	58.3	68.6	699	31.5	24.5	39.4	472
CUB	Cuba	MICS 2010	UMI	LAC	67.2	51.8	79.6	673	30.9	19.0	46.0	989
CUB	Cuba	MICS 2014	UMI	LAC	76.9	66.3	85.0	423	43.6	30.7	57.3	364
CZE	Czech Republic	LIT 2000	UMI	WER	23.8	NA	NA	89278	***	***	***	***
CZE	Czech Republic	LIT 2001	UMI	WER	28.4	NA	NA	89808	***	***	***	***
CZE	Czech Republic	LIT 2002	UMI	WER	31.7	NA	NA	89552	***	***	***	***
CZE	Czech Republic	LIT 2003	UMI	WER	35.1	NA	NA	91299	***	***	***	***
CZE	Czech Republic	LIT 2004	UMI	WER	35.8	NA	NA	92588	***	***	***	***
CZE	Czech Republic	LIT 2005	UMI	WER	38.3	NA	NA	95478	***	***	***	***
CZE	Czech Republic	LIT 2006	HIC	WER	40.9	NA	NA	100624	***	***	***	***
CZE	Czech Republic	LIT 2007	HIC	WER	38.4	NA	NA	104474	***	***	***	***
CZE	Czech Republic	LIT 2008	HIC	WER	36.8	NA	NA	115598	***	***	***	***
CZE	Czech Republic	LIT 2009	HIC	WER	37.4	NA	NA	117268	***	***	***	***
CZE	Czech Republic	LIT 2010	HIC	WER	38.6	NA	NA	114342	***	***	***	***
CZE	Czech Republic	LIT 2011	HIC	WER	40.1	NA	NA	114060	***	***	***	***
CZE	Czech Republic	LIT 2012	HIC	WER	39.6	NA	NA	107707	***	***	***	***
CZE	Czech Republic	LIT 2013	HIC	WER	38.6	NA	NA	109646	***	***	***	***
CZE	Czech Republic	LIT 2014	HIC	WER	36.3	NA	NA	NA	***	***	***	***
CZE	Czech Republic	LIT 2015	HIC	WER	36.9	NA	NA	117478	***	***	***	***
CZE	Czech Republic	LIT 2016	HIC	WER	37.2	NA	NA	118848	***	***	***	***

ISO	Country	Survey and year	Income group[*]	Region of the world[**]	Any breastfeeding at six months			Number of children	Any breastfeeding at one year			Number of children
					Prevalence	95% CI			Prevalence	95% CI		
CZE	Czech Republic	LIT 2017	HIC	WER	38.0	NA	NA	116697	***	***	***	***
DNK	Denmark	LIT 2012	HIC	WER	9.5	NA	NA	29404	***	***	***	***
DNK	Denmark	LIT 2013	HIC	WER	10.1	NA	NA	32882	***	***	***	***
DNK	Denmark	LIT 2014	HIC	WER	11.5	NA	NA	36553	***	***	***	***
DNK	Denmark	LIT 2015	HIC	WER	12.4	NA	NA	37707	***	***	***	***
DNK	Denmark	LIT 2016	HIC	WER	12.0	NA	NA	31372	***	***	***	***
DNK	Denmark	LIT 2017	HIC	WER	11.8	NA	NA	21481	***	***	***	***
DOM	Dominican Republic	DHS 1996	LMI	LAC	60.9	53.6	67.7	275	41.6	34.8	48.8	310
DOM	Dominican Republic	DHS 1999	LMI	LAC	50.4	45.5	55.3	38	40.6	35.4	45.5	37
DOM	Dominican Republic	DHS 2002	LMI	LAC	53.3	47.7	58.8	669	36.0	31.1	41.3	794
DOM	Dominican Republic	DHS 2007	LMI	LAC	61.9	56.3	67.2	768	38.6	31.8	45.8	568
DOM	Dominican Republic	DHS 2013	UMI	LAC	52.4	43.7	61.0	220	40.0	31.9	48.5	284
DOM	Dominican Republic	MICS 2014	UMI	LAC	56.7	52.6	60.7	1426	36.3	31.9	41.0	1250
ECU	Ecuador	RHS 1994	LMI	LAC	86.4	44.4	98.1	537	73.5	61.5	82.8	629
ECU	Ecuador	RHS 1999	LMI	LAC	90.3	86.1	94.5	578	73.8	69.0	78.6	558
ECU	Ecuador	RHS 2004	LMI	LAC	91.5	86.4	96.6	272	77.3	73.4	81.2	284
ECU	Ecuador	DHS 2012	UMI	LAC	87.7	83.4	91.0	774	70.9	65.2	76.0	740
EGY	Egypt	DHS 1995	LMI	MENA	95.3	93.0	96.9	714	85.3	81.8	88.3	755
EGY	Egypt	DHS 2000	LMI	MENA	92.1	89.9	93.9	802	86.4	83.3	88.9	703
EGY	Egypt	DHS 2005	LMI	MENA	93.4	62.2	99.2	923	88.8	81.4	93.4	871
EGY	Egypt	DHS 2008	LMI	MENA	93.0	90.7	94.7	884	89.5	86.4	92.0	715
EGY	Egypt	DHS 2014	LMI	MENA	90.5	88.4	92.3	1170	83.2	80.2	85.8	1003
SLV	El Salvador	RHS 1993	LMI	LAC	77.9	70.1	84.1	187	63.2	55.9	69.9	183
SLV	El Salvador	RHS 1998	LMI	LAC	86.3	81.2	90.2	536	77.9	72.7	82.5	538
SLV	El Salvador	RHS 2002	LMI	LAC	90.9	85.1	94.6	217	73.4	65.7	79.9	236
SLV	El Salvador	RHS 2008	LMI	LAC	85.3	79.8	89.4	324	78.6	71.9	84.0	300
SLV	El Salvador	MICS 2014	LMI	LAC	89.4	84.6	92.8	426	82.1	77.2	86.1	519
EST	Estonia	LIT 2013	HIC	WER	20.7	NA	NA	2820	24.6	NA	NA	3345

ISO	Country	Survey and year	Income group*	Region of the world**	Any breastfeeding at six months			Number of children	Any breastfeeding at one year			Number of children
					Prevalence	95% CI			Prevalence	95% CI		
EST	Estonia	LIT 2014	HIC	WER	28.6	NA	NA	3797	19.0	NA	NA	2524
EST	Estonia	LIT 2015	HIC	WER	36.0	NA	NA	4826	25.5	NA	NA	3410
EST	Estonia	LIT 2016	HIC	WER	44.3	NA	NA	6076	31.9	NA	NA	4378
EST	Estonia	LIT 2017	HIC	WER	46.6	NA	NA	6368	31.6	NA	NA	4317
EST	Estonia	LIT 2018	HIC	WER	48.2	NA	NA	6456	33.7	NA	NA	4518
SWZ	Eswatini	DHS 2006	LMI	ESA	86.3	80.4	90.6	217	84.8	76.8	90.4	184
SWZ	Eswatini	MICS 2010	LMI	ESA	84.9	78.5	89.6	173	66.5	58.4	73.7	180
SWZ	Eswatini	MICS 2014	LMI	ESA	79.5	71.7	85.7	179	57.6	49.9	65.0	187
ETH	Ethiopia	DHS 2000	LI	ESA	99.0	97.4	99.6	713	97.4	94.7	98.7	610
ETH	Ethiopia	DHS 2005	LI	ESA	98.3	96.8	99.1	667	94.6	92.2	96.3	607
ETH	Ethiopia	DHS 2011	LI	ESA	97.6	95.8	98.6	800	97.3	95.5	98.4	639
ETH	Ethiopia	DHS 2016	LI	ESA	95.1	91.7	97.1	752	92.7	89.6	95.0	725
FIN	Finland	LIT 1995	HIC	WER	40.0	NA	NA	NA	25.0	NA	NA	NA
FIN	Finland	LIT 2000	HIC	WER	49.0	NA	NA	1618	37.0	NA	NA	1863
FIN	Finland	LIT 2005	HIC	WER	55.0	NA	NA	1323	38.0	NA	NA	811
FIN	Finland	LIT 2010	HIC	WER	55.0	NA	NA	572	41.0	NA	NA	541
GAB	Gabon	DHS 2000	UMI	WCA	84.9	78.5	89.7	293	64.1	56.6	71.0	259
GAB	Gabon	DHS 2012	UMI	WCA	81.1	72.8	87.3	451	60.5	53.6	67.0	413
GMB	Gambia	MICS 2005	LI	WCA	96.6	94.8	97.8	553	95.6	93.4	97.1	601
GMB	Gambia	MICS 2010	LI	WCA	99.1	98.1	99.6	974	96.0	93.6	97.5	727
GMB	Gambia	DHS 2013	LI	WCA	98.6	96.9	99.3	674	98.0	95.7	99.1	591
GMB	Gambia	MICS 2018	LI	WCA	97.1	93.9	98.6	655	98.6	96.6	99.5	638
GEO	Georgia	MICS 2005	LMI	ECA	60.6	51.0	69.5	144	40.6	31.2	50.8	139
GEO	Georgia	MICS 2018	UMI	ECA	61.8	50.3	72.2	154	45.4	32.6	59.0	152
GHA	Ghana	DHS 1993	LI	WCA	98.6	96.3	99.5	296	96.6	92.6	98.4	203
GHA	Ghana	DHS 1998	LI	WCA	98.9	95.7	99.7	222	97.7	93.8	99.2	201
GHA	Ghana	DHS 2003	LI	WCA	99.6	97.1	99.9	280	98.8	95.9	99.6	268
GHA	Ghana	MICS 2006	LI	WCA	99.2	96.7	99.8	290	97.2	93.5	98.8	212

ISO	Country	Survey and year	Income group[*]	Region of the world[**]	Any breastfeeding at six months			Number of children	Any breastfeeding at one year			Number of children
					Prevalence	95% CI			Prevalence	95% CI		
GHA	Ghana	DHS 2008	LI	WCA	98.5	93.9	99.6	211	95.7	91.3	98.0	216
GHA	Ghana	MICS 2011	LMI	WCA	98.2	95.1	99.4	575	95.2	91.0	97.5	468
GHA	Ghana	DHS 2014	LMI	WCA	98.9	95.5	99.7	463	97.7	94.6	99.0	353
GHA	Ghana	MICS 2017	LMI	WCA	98.7	97.3	99.4	690	96.5	94.2	98.0	535
GRC	Greece	LIT 2007	HIC	WER	22.0	18.9	25.5	586	***	***	***	***
GRC	Greece	LIT 2017	HIC	WER	45.0	40.8	50.0	542	***	***	***	***
GTM	Guatemala	DHS 1995	LMI	LAC	92.4	88.3	95.2	653	81.3	75.9	85.7	690
GTM	Guatemala	DHS 1998	LMI	LAC	93.0	86.2	96.6	313	87.7	79.3	93.0	285
GTM	Guatemala	RHS 2002	LMI	LAC	92.0	87.2	95.1	508	82.7	75.0	88.3	484
GTM	Guatemala	RHS 2008	LMI	LAC	91.5	87.7	94.3	694	85.7	81.7	89.0	712
GTM	Guatemala	DHS 2014	LMI	LAC	94.0	91.5	95.8	849	87.7	84.9	90.0	815
GIN	Guinea	DHS 1999	LI	WCA	98.5	96.4	99.4	422	96.1	93.6	97.7	304
GIN	Guinea	DHS 2005	LI	WCA	98.3	96.4	99.2	470	96.9	94.0	98.4	464
GIN	Guinea	DHS 2012	LI	WCA	97.7	95.7	98.7	597	94.9	92.2	96.8	405
GIN	Guinea	MICS 2016	LI	WCA	98.0	96.6	98.8	536	95.1	92.4	96.9	421
GIN	Guinea	DHS 2018	LI	WCA	91.3	87.9	93.8	480	90.8	87.9	93.1	545
GNB	Guinea Bissau	MICS 2006	LI	WCA	94.9	92.1	96.7	428	94.3	91.5	96.2	403
GNB	Guinea Bissau	MICS 2014	LI	WCA	99.0	97.7	99.6	513	96.9	94.1	98.4	482
GUY	Guyana	MICS 2006	LMI	LAC	75.5	67.0	82.3	173	65.1	55.2	73.7	172
GUY	Guyana	DHS 2009	LMI	LAC	83.9	75.8	89.7	175	65.7	53.8	75.9	141
GUY	Guyana	MICS 2014	LMI	LAC	79.5	72.2	85.2	279	65.5	56.1	73.9	214
HTI	Haiti	DHS 1994	LI	LAC	95.8	91.2	98.0	221	86.3	79.8	90.9	209
HTI	Haiti	DHS 2000	LI	LAC	97.1	94.2	98.6	437	87.0	82.0	90.8	402
HTI	Haiti	DHS 2005	LI	LAC	97.5	95.2	98.7	407	87.9	81.6	92.2	408
HTI	Haiti	DHS 2012	LI	LAC	95.3	92.6	97.1	573	90.7	86.8	93.5	407
HTI	Haiti	DHS 2016	LI	LAC	95.7	92.4	97.6	377	87.0	82.2	90.7	447
HND	Honduras	RHS 1991	LI	LAC	89.9	86.8	92.4	426	76.2	71.9	80.1	425
HND	Honduras	RHS 1996	LI	LAC	88.1	82.6	92.0	258	71.1	63.2	77.8	262

ISO	Country	Survey and year	Income group*	Region of the world**	Any breastfeeding at six months			Number of children	Any breastfeeding at one year			Number of children
					Prevalence	95% CI			Prevalence	95% CI		
HND	Honduras	RHS 2001	LMI	LAC	83.1	78.1	87.1	359	73.6	68.3	78.3	374
HND	Honduras	DHS 2005	LMI	LAC	86.1	82.5	89.0	762	77.6	73.7	81.1	739
HND	Honduras	DHS 2011	LMI	LAC	85.8	82.5	88.5	771	75.7	71.3	79.6	736
IND	India	DHS 1998	LI	SA	97.4	96.8	97.9	3910	92.6	91.3	93.6	3184
IND	India	DHS 2005	LI	SA	97.6	96.9	98.1	3546	91.8	90.4	92.9	3001
IND	India	DHS 2015	LMI	SA	94.7	94.2	95.2	17487	89.6	88.8	90.3	16112
IDN	Indonesia	DHS 1994	LMI	EAP	96.8	95.1	98.0	1152	89.5	86.3	92.0	1161
IDN	Indonesia	DHS 1997	LMI	EAP	94.9	92.9	96.4	1177	91.7	89.6	93.5	1145
IDN	Indonesia	DHS 2002	LI	EAP	90.0	86.4	92.7	1101	85.7	81.0	89.4	1024
IDN	Indonesia	DHS 2007	LMI	EAP	88.3	85.4	90.7	1280	82.2	79.0	85.0	1233
IDN	Indonesia	DHS 2012	LMI	EAP	88.5	85.9	90.7	1227	77.6	74.1	80.8	1246
IDN	Indonesia	DHS 2017	LMI	EAP	84.5	81.8	86.8	1138	75.3	72.1	78.3	1121
IRQ	Iraq	MICS 2006	LMI	MENA	80.5	77.4	83.3	1139	71.4	68.3	74.4	1296
IRQ	Iraq	MICS 2011	LMI	MENA	73.5	70.7	76.1	2526	57.9	54.9	60.9	2495
IRQ	Iraq	MICS 2018	UMI	MENA	64.1	59.0	68.8	1154	54.2	47.9	60.4	981
JAM	Jamaica	MICS 2005	LMI	LAC	81.7	70.3	89.4	77	51.5	41.0	61.8	112
JAM	Jamaica	MICS 2011	UMI	LAC	81.2	71.3	88.3	102	57.9	47.2	67.9	109
JOR	Jordan	DHS 1997	LMI	MENA	82.7	78.3	86.3	411	56.2	50.9	61.3	444
JOR	Jordan	DHS 2002	LMI	MENA	83.3	78.4	87.3	386	58.0	51.9	63.8	409
JOR	Jordan	DHS 2007	LMI	MENA	82.3	77.8	86.1	755	59.7	52.4	66.5	630
JOR	Jordan	DHS 2012	UMI	MENA	75.2	68.6	80.8	626	54.8	48.2	61.3	658
JOR	Jordan	DHS 2017	UMI	MENA	69.1	63.8	73.8	731	42.3	36.1	48.8	596
KAZ	Kazakhstan	DHS 1995	LMI	ECA	69.7	56.1	80.5	91	68.1	53.7	79.6	101
KAZ	Kazakhstan	DHS 1999	LMI	ECA	84.3	72.6	91.6	77	70.6	60.1	79.3	81
KAZ	Kazakhstan	MICS 2006	UMI	ECA	84.0	78.7	88.2	315	69.4	63.0	75.1	325
KAZ	Kazakhstan	MICS 2010	UMI	ECA	86.4	82.1	89.8	393	63.0	56.6	69.0	318
KAZ	Kazakhstan	MICS 2015	UMI	ECA	90.9	87.4	93.6	371	73.2	66.5	79.0	354
KEN	Kenya	DHS 1993	LI	ESA	99.6	98.3	99.9	400	96.4	93.6	98.0	389

ISO	Country	Survey and year	Income group*	Region of the world**	Any breastfeeding at six months			Number of children	Any breastfeeding at one year			Number of children
					Prevalence	95% CI			Prevalence	95% CI		
KEN	Kenya	DHS 1998	LI	ESA	97.4	94.7	98.8	384	94.0	90.5	96.3	374
KEN	Kenya	DHS 2003	LI	ESA	98.2	95.4	99.3	405	95.1	92.6	96.8	402
KEN	Kenya	DHS 2008	LI	ESA	99.3	98.3	99.7	423	89.4	84.0	93.1	376
KEN	Kenya	DHS 2014	LMI	ESA	99.3	97.8	99.8	651	94.9	91.9	96.9	675
KWT	Kuwait	LIT 2011	HIC	MENA	45.3	NA	NA	NA	***	***	***	***
KWT	Kuwait	LIT 2017	HIC	MENA	10.0	NA	NA	2507	11.0	NA	NA	847
KGZ	Kyrgyzstan	DHS 1997	LI	ECA	89.5	79.5	94.9	115	78.0	69.4	84.7	129
KGZ	Kyrgyzstan	MICS 2005	LI	ECA	90.4	82.0	95.1	222	76.5	65.4	84.8	210
KGZ	Kyrgyzstan	DHS 2012	LI	ECA	96.5	93.5	98.1	329	79.6	74.6	83.9	329
KGZ	Kyrgyzstan	MICS 2014	LMI	ECA	91.9	87.6	94.8	317	75.8	70.0	80.8	328
KGZ	Kyrgyzstan	MICS 2018	LMI	ECA	96.2	92.6	98.1	245	81.2	73.4	87.1	222
LAO	Lao	MICS 2006	LI	EAP	93.2	89.2	95.8	290	86.7	81.5	90.6	289
LAO	Lao	MICS 2011	LMI	EAP	89.4	86.3	91.8	733	79.4	75.6	82.8	733
LAO	Lao	MICS 2017	LMI	EAP	85.5	82.4	88.2	798	73.6	69.7	77.3	769
LVA	Latvia	LIT 2008	UMI	WER	49.0	NA	NA	NA	19.0	NA	NA	NA
LVA	Latvia	LIT 2009	HIC	WER	51.0	NA	NA	NA	21.0	NA	NA	NA
LVA	Latvia	LIT 2010	UMI	WER	52.0	NA	NA	11302	22.0	NA	NA	4675
LVA	Latvia	LIT 2011	UMI	WER	53.0	NA	NA	9606	22.0	NA	NA	4062
LVA	Latvia	LIT 2012	HIC	WER	54.0	NA	NA	10410	23.0	NA	NA	4466
LVA	Latvia	LIT 2013	HIC	WER	55.0	NA	NA	11095	24.0	NA	NA	4893
LVA	Latvia	LIT 2014	HIC	WER	57.0	NA	NA	12175	26.0	NA	NA	5588
LVA	Latvia	LIT 2015	HIC	WER	57.0	NA	NA	12671	26.0	NA	NA	5870
LVA	Latvia	LIT 2016	HIC	WER	57.0	NA	NA	12633	26.0	NA	NA	5897
LVA	Latvia	LIT 2017	HIC	WER	58.0	NA	NA	12847	26.0	NA	NA	5853
LVA	Latvia	LIT 2018	HIC	WER	55.5	NA	NA	11301	26.2	NA	NA	5340
LSO	Lesotho	DHS 2004	LI	ESA	95.3	89.9	97.8	240	91.2	86.0	94.6	247
LSO	Lesotho	DHS 2009	LMI	ESA	85.4	79.2	90.0	277	79.8	73.8	84.7	262
LSO	Lesotho	DHS 2014	LMI	ESA	91.0	84.2	95.0	220	79.5	72.9	84.8	249

ISO	Country	Survey and year	Income group*	Region of the world**	Any breastfeeding at six months			Number of children	Any breastfeeding at one year			Number of children
					Prevalence	95% CI			Prevalence	95% CI		
LSO	Lesotho	MICS 2018	LMI	ESA	78.1	70.0	84.6	179	65.0	55.9	73.2	226
LBR	Liberia	DHS 2007	LI	WCA	98.3	96.3	99.2	383	94.3	91.1	96.4	354
LBR	Liberia	DHS 2013	LI	WCA	97.5	93.2	99.1	526	95.2	90.3	97.7	548
LUX	Luxembourg	LIT 2008	HIC	WER	36.0	NA	NA	609	12.0	NA	NA	558
LUX	Luxembourg	LIT 2015	HIC	WER	45.0	NA	NA	685	24.0	NA	NA	1074
MDG	Madagascar	DHS 1997	LI	ESA	99.7	97.9	100.0	395	94.3	91.1	96.4	405
MDG	Madagascar	DHS 2003	LI	ESA	99.3	97.2	99.8	382	94.0	88.7	96.9	328
MDG	Madagascar	DHS 2008	LI	ESA	99.1	98.0	99.6	873	96.1	94.1	97.4	777
MDG	Madagascar	MICS 2018	LI	ESA	98.0	95.7	99.1	915	95.1	93.3	96.5	855
MWI	Malawi	DHS 2000	LI	ESA	99.9	99.6	100.0	858	98.2	96.8	99.0	805
MWI	Malawi	DHS 2004	LI	ESA	99.1	97.5	99.7	788	98.4	96.4	99.3	778
MWI	Malawi	MICS 2006	LI	ESA	98.9	98.3	99.3	1557	97.5	96.2	98.4	1867
MWI	Malawi	DHS 2010	LI	ESA	98.5	97.3	99.1	1291	97.1	95.7	98.0	1242
MWI	Malawi	MICS 2013	LI	ESA	98.0	94.9	99.3	1231	98.9	98.0	99.4	1251
MWI	Malawi	DHS 2015	LI	ESA	94.4	92.6	95.7	1086	92.7	90.7	94.3	1157
MDV	Maldives	DHS 2009	LMI	SA	92.9	88.3	95.8	316	84.5	78.5	89.2	282
MDV	Maldives	DHS 2016	UMI	SA	91.5	85.4	95.2	183	82.1	73.6	88.3	206
MLI	Mali	DHS 1995	LI	WCA	99.5	98.4	99.8	730	97.5	96.0	98.5	659
MLI	Mali	DHS 2001	LI	WCA	98.8	97.8	99.4	969	98.3	97.1	99.0	834
MLI	Mali	DHS 2006	LI	WCA	99.1	98.1	99.6	983	96.2	94.2	97.6	926
MLI	Mali	MICS 2009	LI	WCA	97.5	96.3	98.2	1931	94.7	93.4	95.7	1819
MLI	Mali	DHS 2012	LI	WCA	95.4	92.9	97.0	753	94.1	91.5	95.9	531
MLI	Mali	MICS 2015	LI	WCA	98.5	97.6	99.0	1184	93.8	91.7	95.4	1074
MLI	Mali	DHS 2018	LI	WCA	96.5	94.7	97.7	671	94.1	91.7	95.8	585
MRT	Mauritania	MICS 2007	LI	WCA	90.1	87.2	92.4	628	89.0	85.8	91.5	699
MRT	Mauritania	MICS 2011	LI	WCA	96.4	94.1	97.8	695	89.9	87.0	92.2	678
MRT	Mauritania	MICS 2015	LMI	WCA	95.6	93.2	97.2	700	87.7	84.3	90.4	764
MDA	Moldova	DHS 2005	LMI	ECA	80.4	69.4	88.1	100	54.7	44.9	64.2	124

ISO	Country	Survey and year	Income group[*]	Region of the world[**]	Any breastfeeding at six months			Number of children	Any breastfeeding at one year			Number of children
					Prevalence	95% CI			Prevalence	95% CI		
MDA	Moldova	MICS 2012	LMI	ECA	73.4	64.9	80.4	145	60.5	49.9	70.2	114
MNG	Mongolia	MICS 2005	LI	EAP	92.1	88.0	94.8	304	89.2	84.2	92.8	214
MNG	Mongolia	MICS 2010	LMI	EAP	94.0	89.7	96.5	304	88.8	83.6	92.4	293
MNG	Mongolia	MICS 2013	LMI	EAP	93.6	90.8	95.6	462	86.5	82.2	89.8	384
MNG	Mongolia	MICS 2018	LMI	EAP	93.8	89.6	96.3	440	86.7	80.7	91.1	366
MNE	Montenegro	MICS 2005	UMI	ECA	53.1	39.1	66.7	60	29.7	18.4	44.2	57
MNE	Montenegro	MICS 2013	UMI	ECA	56.0	40.6	70.3	89	38.7	28.7	49.8	81
MNE	Montenegro	MICS 2018	UMI	ECA	61.7	40.5	79.2	77	42.6	26.2	60.8	66
MOZ	Mozambique	DHS 1997	LI	ESA	98.2	94.1	99.5	525	96.1	92.8	97.9	427
MOZ	Mozambique	DHS 2003	LI	ESA	99.1	98.2	99.5	696	96.9	94.9	98.1	623
MOZ	Mozambique	MICS 2008	LI	ESA	98.9	97.9	99.4	851	96.2	94.5	97.4	832
MOZ	Mozambique	DHS 2011	LI	ESA	97.7	96.3	98.6	749	93.0	90.9	94.6	802
MOZ	Mozambique	DHS 2015	LI	ESA	96.9	94.2	98.3	354	94.6	91.8	96.4	344
NAM	Namibia	DHS 2000	LMI	ESA	93.4	89.1	96.1	280	77.6	69.0	84.4	274
NAM	Namibia	DHS 2006	LMI	ESA	86.6	82.0	90.1	392	71.6	64.5	77.8	363
NAM	Namibia	DHS 2013	UMI	ESA	82.6	77.8	86.6	370	74.3	68.5	79.4	301
NPL	Nepal	DHS 1996	LI	SA	100.0	-	-	487	97.4	95.5	98.5	506
NPL	Nepal	DHS 2001	LI	SA	99.7	98.1	100.0	460	98.0	96.3	99.0	398
NPL	Nepal	DHS 2006	LI	SA	100.0	-	-	364	99.0	96.5	99.7	334
NPL	Nepal	DHS 2011	LI	SA	99.5	97.8	99.9	349	95.4	91.6	97.5	314
NPL	Nepal	MICS 2014	LI	SA	96.8	93.7	98.4	362	99.6	98.2	99.9	302
NPL	Nepal	DHS 2016	LI	SA	100.0	-	-	290	97.7	95.2	98.9	332
NLD	Netherlands	LIT 2001	HIC	WER	19.0	NA	NA	NA	***	***	***	***
NLD	Netherlands	LIT 2002	HIC	WER	17.0	NA	NA	NA	***	***	***	***
NLD	Netherlands	LIT 2003	HIC	WER	18.0	NA	NA	NA	***	***	***	***
NLD	Netherlands	LIT 2005	HIC	WER	20.0	NA	NA	NA	***	***	***	***
NLD	Netherlands	LIT 2010	HIC	WER	18.0	NA	NA	NA	***	***	***	***
NLD	Netherlands	LIT 2015	HIC	WER	39.0	33.0	45.0	102	***	***	***	***

ISO	Country	Survey and year	Income group[*]	Region of the world[**]	Any breastfeeding at six months			Number of children	Any breastfeeding at one year			Number of children
					Prevalence	95% CI			Prevalence	95% CI		
NIC	Nicaragua	RHS 1992	LI	LAC	68.6	62.3	74.3	399	47.9	41.5	54.4	379
NIC	Nicaragua	DHS 1998	LI	LAC	75.6	71.3	79.5	539	55.7	50.8	60.6	509
NIC	Nicaragua	DHS 2001	LI	LAC	83.8	78.5	88.0	346	66.6	61.0	71.7	472
NIC	Nicaragua	RHS 2006	LMI	LAC	85.2	80.9	88.6	450	68.8	63.4	73.8	485
NER	Niger	DHS 1998	LI	WCA	99.7	98.7	99.9	520	97.6	95.7	98.7	525
NER	Niger	DHS 2006	LI	WCA	99.5	98.1	99.9	655	97.6	96.0	98.6	617
NER	Niger	DHS 2012	LI	WCA	98.6	97.5	99.3	879	95.8	93.9	97.1	831
NGA	Nigeria	DHS 1999	LI	WCA	95.6	93.5	97.0	381	88.4	85.0	91.2	453
NGA	Nigeria	DHS 2003	LI	WCA	99.0	97.9	99.6	461	92.7	88.2	95.5	386
NGA	Nigeria	MICS 2007	LI	WCA	92.5	90.6	94.0	1146	84.3	81.4	86.8	1417
NGA	Nigeria	DHS 2008	LMI	WCA	96.9	96.0	97.6	2123	91.1	89.6	92.3	1983
NGA	Nigeria	MICS 2011	LMI	WCA	95.4	92.9	97.0	1837	87.9	85.4	90.0	1910
NGA	Nigeria	DHS 2013	LMI	WCA	95.5	94.3	96.4	2190	88.7	87.0	90.2	2198
NGA	Nigeria	MICS 2016	LMI	WCA	97.1	96.0	98.0	1995	91.3	89.4	92.9	1791
NGA	Nigeria	DHS 2018	LMI	WCA	97.9	97.2	98.4	2287	90.6	89.0	92.0	2101
MKD	North Macedonia	MICS 2005	LMI	ECA	87.4	78.0	93.1	165	54.3	40.1	67.8	289
MKD	North Macedonia	MICS 2011	UMI	ECA	67.3	56.3	76.7	94	43.3	28.7	59.1	95
NOR	Norway	LIT 2006	HIC	WER	80.0	NA	NA	1986	46.0	NA	NA	1603
NOR	Norway	LIT 2013	HIC	WER	71.0	NA	NA	2502	35.0	NA	NA	1635
NOR	Norway	LIT 2019	HIC	WER	78.0	NA	NA	2182	48.0	NA	NA	933
OMN	Oman	LIT 2012	HIC	MENA	80.0	NA	NA	NA	***	***	***	***
OMN	Oman	LIT 2017	HIC	MENA	34.3	NA	NA	3129	80.0	NA	NA	3129
PAK	Pakistan	DHS 2006	LI	SA	93.8	91.3	95.6	622	81.0	76.9	84.5	575
PAK	Pakistan	DHS 2012	LMI	SA	87.5	83.6	90.6	711	80.5	76.8	83.7	745
PAK	Pakistan	DHS 2017	LMI	SA	86.0	81.9	89.2	730	71.2	65.9	76.1	623
PRY	Paraguay	RHS 1995	LMI	LAC	72.4	62.9	80.2	153	43.2	34.8	52.0	162
PRY	Paraguay	RHS 1998	LMI	LAC	47.1	26.8	68.5	40	24.0	9.7	48.2	39
PRY	Paraguay	RHS 2004	LMI	LAC	77.0	69.4	83.1	224	48.0	41.1	55.1	298

ISO	Country	Survey and year	Income group[*]	Region of the world[**]	Any breastfeeding at six months			Number of children	Any breastfeeding at one year			Number of children
					Prevalence	95% CI			Prevalence	95% CI		
PRY	Paraguay	RHS 2008	LMI	LAC	75.2	67.2	81.8	208	50.9	42.0	59.7	182
PRY	Paraguay	MICS 2016	UMI	LAC	85.9	80.7	89.9	328	62.5	54.9	69.5	292
PER	Peru	DHS 1996	LMI	LAC	95.6	93.5	97.1	1065	84.0	80.8	86.8	1175
PER	Peru	DHS 2000	LMI	LAC	94.9	92.0	96.8	784	85.4	81.9	88.4	877
PER	Peru	DHS 2004	LMI	LAC	98.7	93.1	99.7	155	83.5	74.4	89.8	159
PER	Peru	DHS 2005	LMI	LAC	93.2	86.3	96.8	180	85.4	76.6	91.2	197
PER	Peru	DHS 2006	LMI	LAC	98.5	96.2	99.4	176	87.1	77.2	93.1	177
PER	Peru	DHS 2007	LMI	LAC	98.4	94.5	99.5	158	87.1	77.2	93.1	177
PER	Peru	DHS 2008	UMI	LAC	97.3	92.5	99.1	394	92.4	88.2	95.2	419
PER	Peru	DHS 2009	UMI	LAC	98.2	96.4	99.1	716	86.2	82.0	89.6	726
PER	Peru	DHS 2010	UMI	LAC	98.3	96.7	99.1	579	91.7	87.9	94.3	620
PER	Peru	DHS 2011	UMI	LAC	98.5	97.0	99.2	576	86.8	82.2	90.4	575
PER	Peru	DHS 2012	UMI	LAC	94.2	90.4	96.6	604	87.2	82.5	90.7	616
PER	Peru	DHS 2013	UMI	LAC	96.8	93.0	98.6	590	88.9	83.9	92.5	578
PER	Peru	DHS 2014	UMI	LAC	94.4	91.0	96.5	593	87.8	84.2	90.7	661
PER	Peru	DHS 2015	UMI	LAC	95.0	93.1	96.4	1527	87.2	84.6	89.4	1534
PER	Peru	DHS 2016	UMI	LAC	93.7	91.1	95.5	1337	86.7	83.8	89.2	1390
PER	Peru	DHS 2017	UMI	LAC	96.1	94.4	97.3	1415	88.1	85.3	90.3	1501
PER	Peru	DHS 2018	UMI	LAC	95.0	93.1	96.3	1425	89.5	87.3	91.4	1439
PHL	Philippines	DHS 1993	LMI	EAP	72.9	69.3	76.3	647	60.3	56.0	64.3	561
PHL	Philippines	DHS 1998	LMI	EAP	68.2	63.2	72.8	575	58.7	53.8	63.3	475
PHL	Philippines	DHS 2003	LMI	EAP	69.5	64.9	73.7	462	58.3	53.6	63.0	450
PHL	Philippines	DHS 2008	LMI	EAP	69.4	63.6	74.6	415	60.2	55.0	65.1	425
PHL	Philippines	DHS 2013	LMI	EAP	77.8	73.1	81.8	462	61.5	56.6	66.2	480
PHL	Philippines	DHS 2017	LMI	EAP	79.4	73.5	84.3	622	72.3	65.9	77.8	658
PRT	Portugal	LIT 2006	HIC	WER	38.6	NA	NA	NA	***	***	***	***
PRT	Portugal	LIT 2014	HIC	WER	30.3	NA	NA	NA	***	***	***	***
KOR	Republic of Korea	LIT 2006	HIC	EAP	25.2	NA	NA	747	23.7	NA	NA	579

ISO	Country	Survey and year	Income group*	Region of the world**	Any breastfeeding at six months			Number of children	Any breastfeeding at one year			Number of children
					Prevalence	95% CI			Prevalence	95% CI		
KOR	Republic of Korea	LIT 2009	HIC	EAP	34.7	NA	NA	799	24.5	NA	NA	626
KOR	Republic of Korea	LIT 2012	HIC	EAP	35.7	NA	NA	768	29.5	NA	NA	605
KOR	Republic of Korea	LIT 2015	HIC	EAP	33.5	NA	NA	1545	29.5	NA	NA	1229
KOR	Republic of Korea	LIT 2018	HIC	EAP	26.0	NA	NA	1533	7.3	NA	NA	1212
RWA	Rwanda	DHS 2000	LI	ESA	99.8	98.9	100.0	563	97.3	95.6	98.3	554
RWA	Rwanda	DHS 2005	LI	ESA	99.2	98.0	99.7	565	96.2	94.3	97.5	595
RWA	Rwanda	DHS 2010	LI	ESA	99.1	97.8	99.6	559	95.7	93.6	97.2	530
RWA	Rwanda	DHS 2014	LI	ESA	99.4	98.4	99.8	598	97.7	96.0	98.7	493
STP	Sao Tome and Principe	DHS 2008	LMI	WCA	99.6	96.8	99.9	156	95.8	91.0	98.1	107
STP	Sao Tome and Principe	MICS 2014	LMI	WCA	96.9	90.7	99.0	111	93.4	86.0	97.0	142
SEN	Senegal	DHS 1997	LI	WCA	99.2	97.8	99.7	521	95.8	91.1	98.1	478
SEN	Senegal	DHS 2005	LI	WCA	99.1	97.0	99.7	959	95.0	92.1	96.9	549
SEN	Senegal	DHS 2010	LMI	WCA	99.4	98.2	99.8	819	98.4	96.5	99.3	851
SEN	Senegal	DHS 2012	LMI	WCA	99.5	98.4	99.9	469	99.4	98.5	99.8	428
SEN	Senegal	DHS 2014	LMI	WCA	98.7	96.9	99.4	466	94.5	88.3	97.5	401
SEN	Senegal	DHS 2015	LI	WCA	97.2	93.0	98.9	491	98.0	95.5	99.2	440
SEN	Senegal	DHS 2016	LI	WCA	99.5	98.0	99.9	430	97.2	94.5	98.6	441
SEN	Senegal	DHS 2017	LI	WCA	97.5	95.9	98.5	783	95.4	92.9	97.0	781
SRB	Serbia	MICS 2005	UMI	ECA	66.6	58.4	73.9	256	30.5	24.2	37.5	246
SRB	Serbia	MICS 2010	UMI	ECA	57.4	48.5	65.7	214	29.5	21.0	39.9	223
SRB	Serbia	MICS 2014	UMI	ECA	70.2	59.4	79.1	197	43.6	32.9	54.8	139
SLE	Sierra Leone	MICS 2005	LI	WCA	94.7	92.1	96.5	424	86.9	82.5	90.4	323
SLE	Sierra Leone	DHS 2008	LI	WCA	94.5	91.1	96.6	435	86.6	82.0	90.1	405
SLE	Sierra Leone	MICS 2010	LI	WCA	96.6	94.7	97.9	620	87.1	83.4	90.1	553
SLE	Sierra Leone	DHS 2013	LI	WCA	94.7	92.6	96.2	829	87.8	84.8	90.3	751
SLE	Sierra Leone	MICS 2017	LI	WCA	98.2	97.0	98.9	738	89.2	86.0	91.7	780
SGP	Singapore	LIT 2001	HIC	EAP	21.1	NA	NA	NA	***	***	***	***
SGP	Singapore	LIT 2011	HIC	EAP	41.6	NA	NA	NA	***	***	***	***

ISO	Country	Survey and year	Income group[*]	Region of the world[**]	Any breastfeeding at six months			Number of children	Any breastfeeding at one year			Number of children
					Prevalence	95% CI			Prevalence	95% CI		
ZAF	South Africa	DHS 1998	LMI	ESA	77.8	72.1	82.6	365	73.1	66.5	78.9	301
ZAF	South Africa	DHS 2016	UMI	ESA	66.0	57.1	74.0	225	52.9	44.0	61.6	186
ESP	Spain	LIT 2001	HIC	WER	45.0	NA	NA	NA	***	***	***	***
ESP	Spain	LIT 2006	HIC	WER	39.0	NA	NA	NA	***	***	***	***
ESP	Spain	LIT 2011	HIC	WER	47.0	NA	NA	NA	***	***	***	***
SUR	Suriname	MICS 2006	LMI	LAC	60.7	51.8	68.9	161	46.9	37.4	56.7	143
SUR	Suriname	MICS 2010	UMI	LAC	60.9	52.6	68.7	216	37.0	29.8	44.9	201
SUR	Suriname	MICS 2018	UMI	LAC	61.1	52.8	68.7	260	41.0	33.6	49.0	269
SWE	Sweden	LIT 2010	HIC	WER	62.5	NA	NA	70329	16.2	NA	NA	17735
SWE	Sweden	LIT 2011	HIC	WER	63.1	NA	NA	68956	17.2	NA	NA	18516
SWE	Sweden	LIT 2012	HIC	WER	62.7	NA	NA	69061	18.0	NA	NA	19533
SWE	Sweden	LIT 2013	HIC	WER	63.2	NA	NA	68954	19.1	NA	NA	20834
SWE	Sweden	LIT 2014	HIC	WER	63.0	NA	NA	71761	***	***	***	***
SWE	Sweden	LIT 2015	HIC	WER	63.2	NA	NA	70615	24.1	NA	NA	26475
SWE	Sweden	LIT 2016	HIC	WER	64.0	NA	NA	72662	26.2	NA	NA	29230
CHE	Switzerland	LIT 2003	HIC	WER	27.0	NA	NA	2889	0.0	-	-	1547
CHE	Switzerland	LIT 2014	HIC	WER	27.0	NA	NA	1535	0.0	-	-	1421
TJK	Tajikistan	MICS 2005	LI	ECA	90.7	86.1	93.9	282	81.0	76.1	85.1	326
TJK	Tajikistan	DHS 2012	LI	ECA	92.2	88.0	95.0	334	82.3	77.1	86.5	369
TJK	Tajikistan	DHS 2017	LI	ECA	92.9	89.7	95.1	383	81.0	76.5	84.8	439
TZA	Tanzania	DHS 1996	LI	ESA	98.3	96.3	99.2	462	95.9	93.3	97.5	467
TZA	Tanzania	DHS 1999	LI	ESA	97.5	92.9	99.1	197	96.1	90.6	98.4	167
TZA	Tanzania	DHS 2004	LI	ESA	97.2	94.7	98.5	578	95.1	92.0	97.0	565
TZA	Tanzania	DHS 2010	LI	ESA	97.6	95.0	98.9	539	95.8	93.1	97.5	519
TZA	Tanzania	DHS 2015	LI	ESA	98.0	96.2	98.9	683	95.6	93.3	97.1	666
THA	Thailand	MICS 2005	LMI	EAP	56.7	51.4	61.7	682	43.4	38.0	49.0	613
THA	Thailand	MICS 2012	UMI	EAP	52.4	44.5	60.1	334	35.8	30.4	41.6	665
THA	Thailand	MICS 2015	UMI	EAP	55.6	45.4	65.4	460	39.1	30.2	48.9	610

ISO	Country	Survey and year	Income group*	Region of the world**	Any breastfeeding at six months			Number of children	Any breastfeeding at one year			Number of children
					Prevalence	95% CI			Prevalence	95% CI		
TLS	Timor-Leste	DHS 2009	LMI	EAP	97.5	95.7	98.5	676	81.9	78.4	85.0	605
TLS	Timor-Leste	DHS 2016	LMI	EAP	89.4	85.7	92.2	503	71.7	66.4	76.5	443
TGO	Togo	DHS 1998	LI	WCA	99.2	98.0	99.7	517	99.6	98.4	99.9	423
TGO	Togo	MICS 2006	LI	WCA	64.5	57.7	70.7	295	72.9	66.3	78.7	319
TGO	Togo	MICS 2010	LI	WCA	98.7	96.7	99.5	342	94.9	90.7	97.3	339
TGO	Togo	DHS 2013	LI	WCA	98.2	95.9	99.2	477	96.7	94.0	98.2	458
TGO	Togo	MICS 2017	LI	WCA	98.9	96.7	99.7	320	95.5	91.7	97.6	348
TTO	Trinidad and Tobago	MICS 2006	HIC	LAC	55.2	43.9	66.0	82	42.1	29.5	55.8	62
TTO	Trinidad and Tobago	MICS 2011	HIC	LAC	67.4	53.7	78.7	69	41.9	30.2	54.5	69
TUN	Tunisia	MICS 2011	UMI	MENA	76.5	67.9	83.4	212	53.7	44.9	62.2	177
TUN	Tunisia	MICS 2018	LMI	MENA	70.0	62.5	76.5	199	54.4	46.4	62.2	185
TUR	Turkey	DHS 1993	LMI	ECA	74.7	69.1	79.6	268	51.8	45.3	58.3	256
TUR	Turkey	DHS 1998	UMI	ECA	78.6	72.1	83.9	236	51.7	43.7	59.5	245
TUR	Turkey	DHS 2003	LMI	ECA	83.9	79.0	87.9	314	60.5	53.1	67.4	281
TUR	Turkey	DHS 2013	UMI	ECA	87.0	80.5	91.6	266	69.0	61.2	76.0	223
TKM	Turkmenistan	MICS 2006	LMI	ECA	94.2	88.7	97.1	139	81.0	73.6	86.7	152
TKM	Turkmenistan	MICS 2015	UMI	ECA	94.8	91.3	97.0	246	81.7	76.2	86.1	264
UGA	Uganda	DHS 1995	LI	ESA	98.2	96.4	99.1	468	92.8	90.2	94.8	541
UGA	Uganda	DHS 2000	LI	ESA	98.9	97.0	99.6	475	93.0	89.9	95.1	493
UGA	Uganda	DHS 2006	LI	ESA	99.4	98.4	99.8	535	94.8	92.4	96.4	536
UGA	Uganda	DHS 2011	LI	ESA	97.0	94.5	98.3	546	91.8	88.6	94.2	489
UGA	Uganda	DHS 2016	LI	ESA	98.1	96.9	98.9	1001	91.6	89.0	93.7	955
UKR	Ukraine	MICS 2005	LMI	ECA	69.1	60.2	76.9	203	35.7	28.0	44.3	196
UKR	Ukraine	DHS 2007	LMI	ECA	66.0	54.6	75.8	84	37.7	26.2	50.8	85
UKR	Ukraine	MICS 2012	LMI	ECA	78.9	68.7	86.4	281	44.1	35.6	53.0	259
GBR	United Kingdom	LIT 2000	HIC	WER	21.0	NA	NA	7267	***	***	***	***
GBR	United Kingdom	LIT 2005	HIC	WER	25.0	NA	NA	9416	***	***	***	***
GBR	United Kingdom	LIT 2010	HIC	WER	34.0	NA	NA	10769	***	***	***	***

ISO	Country	Survey and year	Income group*	Region of the world**	Any breastfeeding at six months			Number of children	Any breastfeeding at one year			Number of children
					Prevalence	95% CI			Prevalence	95% CI		
USA	United States	LIT 2000	HIC	NAM	35.0	32.5	36.5	12017	16.0	14.5	17.6	12017
USA	United States	LIT 2001	HIC	NAM	37.0	36.2	38.5	28762	19.0	17.6	19.4	28762
USA	United States	LIT 2002	HIC	NAM	38.0	36.9	38.9	27427	19.0	18.4	20.0	27427
USA	United States	LIT 2003	HIC	NAM	39.0	38.2	40.0	29249	20.0	18.9	20.4	29249
USA	United States	LIT 2004	HIC	NAM	43.0	41.1	43.0	30975	21.0	20.6	22.2	30975
USA	United States	LIT 2005	HIC	NAM	43.0	41.8	44.0	23713	21.0	20.6	22.4	23713
USA	United States	LIT 2006	HIC	NAM	44.0	42.4	44.5	24866	23.0	21.8	23.6	24866
USA	United States	LIT 2007	HIC	NAM	44.0	42.7	44.9	24762	23.0	21.7	23.6	24762
USA	United States	LIT 2008	HIC	NAM	44.0	43.3	45.4	24622	23.0	22.5	24.4	24622
USA	United States	LIT 2009	HIC	NAM	47.0	45.4	47.8	23542	25.0	23.6	25.6	23542
USA	United States	LIT 2010	HIC	NAM	47.5	46.1	48.9	15912	25.0	24.0	26.5	15912
USA	United States	LIT 2011	HIC	NAM	49.4	47.9	50.9	14456	27.0	25.4	28.1	14456
USA	United States	LIT 2012	HIC	NAM	51.0	49.8	52.9	15141	29.0	27.7	30.6	15141
USA	United States	LIT 2013	HIC	NAM	51.8	50.4	53.2	16441	31.0	29.5	32.0	16441
USA	United States	LIT 2014	HIC	NAM	55.3	53.9	56.7	17109	34.0	32.3	35.0	17109
USA	United States	LIT 2015	HIC	NAM	58.0	56.2	58.9	17673	36.0	34.6	37.2	17673
USA	United States	LIT 2016	HIC	NAM	57.0	55.7	58.8	17604	36.2	34.7	37.7	17604
URY	Uruguay	LIT 1996	UMI	LAC	***	***	***	***	24.6	NA	NA	1649
URY	Uruguay	LIT 1999	UMI	LAC	***	***	***	***	40.2	NA	NA	1382
URY	Uruguay	LIT 2003	UMI	LAC	***	***	***	***	47.0	NA	NA	1688
URY	Uruguay	LIT 2007	UMI	LAC	***	***	***	***	43.5	NA	NA	1622
URY	Uruguay	LIT 2011	UMI	LAC	***	***	***	***	44.8	NA	NA	1678
UZB	Uzbekistan	DHS 1996	LMI	ECA	89.7	82.1	94.3	133	75.4	68.2	81.5	162
UZB	Uzbekistan	MICS 2006	LI	ECA	93.3	88.8	96.1	339	83.7	78.8	87.7	355
VNM	Vietnam	DHS 1997	LI	EAP	97.5	93.6	99.1	190	90.4	85.3	93.8	210
VNM	Vietnam	DHS 2002	LI	EAP	95.8	89.5	98.4	125	89.8	76.9	95.9	151
VNM	Vietnam	MICS 2006	LI	EAP	96.2	89.7	98.6	152	85.2	78.1	90.3	194
VNM	Vietnam	MICS 2010	LMI	EAP	94.8	90.1	97.3	207	83.6	78.3	87.8	252

ISO	Country	Survey and year	Income group*	Region of the world**	Any breastfeeding at six months			Number of children	Any breastfeeding at one year			Number of children
					Prevalence	95% CI			Prevalence	95% CI		
VNM	Vietnam	MICS 2013	LMI	EAP	95.0	91.8	97.1	255	81.0	74.8	86.0	244
ZMB	Zambia	DHS 1996	LI	ESA	99.1	97.5	99.7	422	96.2	94.1	97.6	511
ZMB	Zambia	DHS 2001	LI	ESA	99.1	97.0	99.7	444	98.2	96.0	99.2	426
ZMB	Zambia	DHS 2007	LI	ESA	99.2	97.5	99.7	416	96.0	93.7	97.5	421
ZMB	Zambia	DHS 2013	LMI	ESA	98.2	97.0	98.9	885	94.8	92.6	96.3	833
ZMB	Zambia	DHS 2018	LMI	ESA	98.1	96.8	98.9	648	93.6	90.8	95.6	636
ZWE	Zimbabwe	DHS 1994	LI	ESA	99.3	97.2	99.8	267	92.6	86.7	96.0	247
ZWE	Zimbabwe	DHS 1999	LI	ESA	99.5	96.7	99.9	215	96.5	92.4	98.5	239
ZWE	Zimbabwe	DHS 2005	LI	ESA	98.0	95.8	99.0	368	95.9	92.3	97.8	341
ZWE	Zimbabwe	MICS 2009	LI	ESA	96.6	93.8	98.1	479	88.3	84.5	91.3	465
ZWE	Zimbabwe	DHS 2010	LI	ESA	97.4	95.7	98.4	487	91.4	87.9	93.9	430
ZWE	Zimbabwe	MICS 2014	LI	ESA	97.2	95.4	98.3	692	93.1	90.6	94.9	619
ZWE	Zimbabwe	DHS 2015	LI	ESA	99.5	98.4	99.8	371	93.0	89.4	95.5	351
ZWE	Zimbabwe	MICS 2019	LMI	ESA	98.6	96.8	99.4	426	91.2	87.4	94.0	376

*LI: low income; LMI: lower-middle income; UMI: Upper-middle income; HIC: High income.

**EAP: East Asia and Pacific; ESA: Eastern and Southern Africa; ECA: Eastern Europe and Central Asia; LAC: Latin America and Caribbean; MENA: Middle East and North Africa; NAM: North America; SA: South Asia; WCA: West and Central Africa; WER: Western Europe.

***Data not available in the survey.

NA: figures not provided in the official report.

Supplementary table 2. Countries, surveys, prevalence, and number of children included in the trend analysis of exclusive breastfeeding and formula consumption indicators in low- and-middle income countries. Source: Demographic Health Survey (DHS), Multiple Indicator Cluster Survey (MICS), Reproductive and Health Survey (RHS).

ISO	Country	Survey and year	Income group*	Region of the world**	Exclusive breastfeeding under six months			Formula consumption under six months			Number of children	Formula consumption between 6-23 months			Number of children
					Prevalence	95% CI		Prevalence	95% CI			Prevalence	95% CI		
AFG	Afghanistan	MICS 2010	LI	SA	54.3	50.7	57.9	15.7	13.1	18.7	1270	19.0	17.1	21.1	3635
AFG	Afghanistan	DHS 2015	LI	SA	43.3	40.3	46.4	8.5	6.9	10.5	3203	12.4	10.9	13.9	8078
ALB	Albania	RHS 2002	LMI	ECA	45.0	33.6	56.9	***	***	***	170	***	***	***	***
ALB	Albania	MICS 2005	LMI	ECA	3.4	1.1	10.1	1.9	0.4	7.7	99	2.5	1.3	4.6	273
ALB	Albania	DHS 2008	LMI	ECA	38.6	28.3	50.0	16.3	10.2	25.0	138	13.9	10.1	18.9	386
ALB	Albania	DHS 2017	UMI	ECA	36.7	30.0	44.1	24.0	17.2	32.4	285	12.2	9.1	16.0	766
ARM	Armenia	DHS 2000	LI	ECA	29.9	22.6	38.4	13.2	7.9	21.3	155	8.4	5.7	12.3	435
ARM	Armenia	DHS 2005	LMI	ECA	32.5	23.0	43.6	14.3	8.3	23.6	157	19.5	14.6	25.6	391
ARM	Armenia	DHS 2010	LMI	ECA	34.6	26.2	44.0	15.6	9.9	23.8	156	10.0	7.1	13.9	464
ARM	Armenia	DHS 2015	LMI	ECA	44.5	36.6	52.7	3.4	1.6	6.9	177	5.0	3.1	7.9	499
BGD	Bangladesh	DHS 1993	LI	SA	46.1	42.0	50.2	14.7	12.0	18.0	616	22.5	20.4	24.9	1688
BGD	Bangladesh	DHS 1996	LI	SA	45.0	40.8	49.3	12.9	10.2	16.2	612	18.4	16.2	21.0	1593
BGD	Bangladesh	DHS 1999	LI	SA	46.5	42.9	50.1	5.8	4.3	7.8	746	7.4	6.2	8.8	1786
BGD	Bangladesh	DHS 2004	LI	SA	41.6	37.8	45.4	10.8	8.6	13.5	679	8.2	7.0	9.6	1791
BGD	Bangladesh	MICS 2006	LI	SA	37.4	35.0	39.9	7.1	5.9	8.7	2300	8.2	7.5	9.1	9453
BGD	Bangladesh	DHS 2007	LI	SA	42.9	38.0	48.0	9.4	6.9	12.7	510	11.0	9.2	13.2	1718
BGD	Bangladesh	DHS 2011	LI	SA	64.1	59.9	68.1	11.4	8.9	14.4	789	6.3	5.2	7.6	2356
BGD	Bangladesh	MICS 2012	LI	SA	56.4	53.6	59.1	8.6	7.1	10.4	1959	11.5	10.3	12.8	5968
BGD	Bangladesh	DHS 2014	LMI	SA	55.3	48.8	61.6	10.1	7.2	13.9	632	6.9	5.6	8.5	2336
BGD	Bangladesh	MICS 2019	LMI	SA	63.3	61.0	65.4	13.3	11.8	15.0	2370	10.3	9.4	11.2	6691
BLR	Belarus	MICS 2005	LMI	ECA	10.3	5.5	18.4	65.2	54.9	74.1	183	44.4	41.0	47.8	986
BLR	Belarus	MICS 2012	UMI	ECA	19.0	12.9	27.2	50.3	41.9	58.6	247	41.3	37.4	45.4	1115
BLZ	Belize	RHS 1991	LMI	LAC	18.1	10.5	29.5	21.1	12.1	34.3	232	14.2	10.7	18.6	612
BLZ	Belize	MICS 2006	UMI	LAC	10.1	5.0	19.3	54.0	42.9	64.6	88	59.9	53.4	66.1	239
BLZ	Belize	MICS 2011	LMI	LAC	14.7	10.1	20.9	57.4	49.3	65.0	145	37.0	32.6	41.5	610

ISO	Country	Survey and year	Income group[*]	Region of the world[**]	Exclusive breastfeeding under six months			Formula consumption under six months			Number of children	Formula consumption between 6-23 months			Number of children
					Prevalence	95% CI		Prevalence	95% CI			Prevalence	95% CI		
BLZ	Belize	MICS 2015	UMI	LAC	33.2	24.7	42.9	42.0	32.9	51.8	165	45.5	41.2	49.9	730
BEN	Benin	DHS 1996	LI	WCA	10.1	7.3	13.9	3.5	2.0	6.1	518	5.6	4.2	7.5	1370
BEN	Benin	DHS 2001	LI	WCA	37.9	33.6	42.5	21.5	18.1	25.4	527	17.8	15.2	20.7	1436
BEN	Benin	DHS 2006	LI	WCA	43.1	40.2	46.1	13.7	11.9	15.6	1525	20.8	19.2	22.6	4593
BEN	Benin	DHS 2011	LI	WCA	33.1	30.3	36.1	7.6	6.0	9.6	1195	6.8	5.9	7.9	3739
BEN	Benin	MICS 2014	LI	WCA	41.4	38.0	44.8	1.2	0.7	1.9	1284	4.3	3.4	5.4	3639
BEN	Benin	DHS 2017	LI	WCA	41.5	38.8	44.3	3.5	2.6	4.8	1381	5.0	4.1	6.1	3884
BOL	Bolivia	DHS 1994	LMI	LAC	43.3	38.4	48.5	8.0	5.6	11.3	556	3.1	2.2	4.5	1622
BOL	Bolivia	DHS 1998	LMI	LAC	50.6	46.1	55.1	12.0	9.4	15.2	629	7.7	6.3	9.5	1984
BOL	Bolivia	DHS 2003	LMI	LAC	53.6	49.2	57.9	18.8	15.6	22.4	867	13.3	11.8	15.0	2703
BOL	Bolivia	DHS 2008	LMI	LAC	60.4	56.5	64.2	20.8	17.8	24.1	791	31.2	28.7	33.8	2461
BIH	Bosnia and Herzegovina	MICS 2006	LMI	ECA	17.6	10.4	28.3	29.9	20.7	40.9	186	49.7	44.4	55.1	1008
BIH	Bosnia and Herzegovina	MICS 2011	UMI	ECA	18.5	10.9	29.7	32.1	20.9	45.9	117	15.0	11.2	19.7	635
BFA	Burkina Faso	DHS 1998	LI	WCA	5.5	3.7	8.1	1.1	0.6	2.3	618	0.6	0.3	1.1	1491
BFA	Burkina Faso	DHS 2003	LI	WCA	18.8	15.8	22.3	5.0	3.6	6.9	1071	4.3	3.5	5.3	2782
BFA	Burkina Faso	MICS 2006	LI	WCA	6.8	4.5	10.3	0.4	0.1	1.7	543	0.8	0.4	1.6	1708
BFA	Burkina Faso	DHS 2010	LI	WCA	24.8	22.1	27.8	0.8	0.4	1.5	1454	2.2	1.7	2.9	4148
BDI	Burundi	DHS 2010	LI	ESA	69.3	64.5	73.7	0.5	0.2	1.2	685	0.8	0.5	1.3	2196
BDI	Burundi	DHS 2016	LI	ESA	83.1	80.7	85.2	0.5	0.2	1.1	1236	0.2	0.1	0.5	3858
CAF	Central Afr. Republic	DHS 1994	LI	WCA	3.0	1.6	5.4	1.6	0.9	3.0	458	1.5	1.0	2.2	1215
CAF	Central Afr. Republic	MICS 2006	LI	WCA	23.3	19.4	27.7	7.2	5.6	9.3	1266	11.0	9.1	13.2	2759
CAF	Central Afr. Republic	MICS 2010	LI	WCA	33.7	30.0	37.6	5.6	4.1	7.6	1283	7.5	6.2	9.1	3266
KHM	Cambodia	DHS 2000	LI	EAP	11.4	8.9	14.6	3.3	2.1	5.1	873	4.9	3.8	6.3	2099
KHM	Cambodia	DHS 2005	LI	EAP	60.0	55.7	64.2	7.1	5.0	9.9	788	6.5	5.1	8.3	2329
KHM	Cambodia	DHS 2010	LI	EAP	73.5	69.2	77.3	6.8	4.8	9.5	717	9.3	7.8	11.1	2368
KHM	Cambodia	DHS 2014	LI	EAP	65.2	60.5	69.6	11.5	9.0	14.4	688	13.4	11.8	15.3	2127
CMR	Cameroon	DHS 1998	LI	WCA	12.2	8.8	16.5	8.9	6.1	12.8	389	8.9	7.1	11.1	1009
CMR	Cameroon	DHS 2004	LI	WCA	23.5	20.1	27.2	7.7	5.9	9.9	794	8.5	7.2	10.0	2150

ISO	Country	Survey and year	Income group*	Region of the world**	Exclusive breastfeeding under six months			Formula consumption under six months			Number of children	Formula consumption between 6-23 months			Number of children
					Prevalence	95% CI		Prevalence	95% CI			Prevalence	95% CI		
CMR	Cameroon	MICS 2006	LMI	WCA	21.2	17.3	25.8	5.0	3.1	7.8	658	2.9	2.0	4.0	2053
CMR	Cameroon	DHS 2011	LMI	WCA	20.2	17.6	23.1	6.6	5.2	8.3	1123	6.8	5.7	8.0	3287
CMR	Cameroon	MICS 2014	LMI	WCA	28.2	24.5	32.3	8.7	6.5	11.4	703	6.3	4.8	8.1	2128
CMR	Cameroon	DHS 2018	LMI	WCA	39.7	36.0	43.5	8.1	6.3	10.4	986	11.3	9.7	13.1	2576
TCD	Chad	DHS 1996	LI	WCA	1.9	1.2	3.1	5.7	4.1	7.8	807	11.3	9.6	13.2	1813
TCD	Chad	DHS 2004	LI	WCA	2.0	1.0	4.2	0.9	0.4	2.0	583	4.9	3.7	6.4	1371
TCD	Chad	MICS 2010	LI	WCA	3.4	2.5	4.6	3.7	2.8	5.0	1867	5.4	4.5	6.3	4579
TCD	Chad	DHS 2014	LI	WCA	0.3	0.1	0.7	4.7	3.7	5.9	1823	4.1	3.4	4.9	4403
COL	Colombia	DHS 1995	LMI	LAC	11.5	8.6	15.1	41.2	36.2	46.2	440	14.5	12.7	16.5	1454
COL	Colombia	DHS 2000	LMI	LAC	25.8	21.6	30.6	39.9	35.0	45.0	430	38.1	35.3	41.0	1335
COL	Colombia	DHS 2005	LMI	LAC	47.0	43.3	50.8	30.3	27.0	33.8	1397	38.7	36.6	40.8	4110
COL	Colombia	DHS 2010	UMI	LAC	42.9	39.7	46.2	35.2	32.0	38.6	1515	42.5	40.6	44.3	5023
COM	Comoros	DHS 1996	LI	ESA	3.2	1.4	7.0	25.8	19.6	33.2	186	21.8	18.0	26.2	522
COM	Comoros	DHS 2012	LI	ESA	12.1	8.1	17.8	26.5	21.2	32.6	327	16.6	14.0	19.6	869
COD	Congo Dem. Republic	DHS 2007	LI	WCA	36.1	31.1	41.4	16.4	12.9	20.7	907	23.3	19.6	27.3	2355
COD	Congo Dem. Republic	MICS 2010	LI	WCA	37.0	33.6	40.5	3.8	2.4	5.8	1271	2.9	2.4	3.7	3617
COD	Congo Dem. Republic	DHS 2013	LI	WCA	47.6	44.2	51.1	3.2	2.2	4.5	1934	2.7	2.0	3.6	4991
COD	Congo Dem. Republic	MICS 2017	LI	WCA	54.6	50.5	58.7	5.2	3.7	7.3	2106	5.2	3.9	6.7	6499
CIV	Cote d'Ivoire	DHS 1994	LI	WCA	3.0	1.9	4.7	4.6	3.1	6.7	658	2.7	1.9	3.7	1710
CIV	Cote d'Ivoire	DHS 1998	LI	WCA	3.5	1.2	9.7	14.8	9.7	22.1	166	19.3	15.3	24.1	583
CIV	Cote d'Ivoire	MICS 2006	LI	WCA	4.3	2.6	7.0	4.8	3.2	7.0	950	9.3	7.3	11.9	2765
CIV	Cote d'Ivoire	DHS 2011	LMI	WCA	12.2	9.7	15.4	5.8	4.1	8.1	775	5.4	4.2	7.0	2145
CIV	Cote d'Ivoire	MICS 2016	LMI	WCA	23.5	20.2	27.1	5.6	3.5	9.0	981	4.8	3.8	6.1	2668
CUB	Cuba	MICS 2006	LMI	LAC	26.4	22.3	30.8	11.6	8.2	16.1	774	12.7	10.4	15.4	2712
CUB	Cuba	MICS 2010	UMI	LAC	48.6	38.8	58.6	10.0	5.6	17.3	1002	15.1	10.2	21.8	3600
CUB	Cuba	MICS 2014	UMI	LAC	33.2	21.0	48.1	5.3	1.7	15.1	381	11.2	7.3	16.9	1775
DOM	Dominican Republic	DHS 1996	LMI	LAC	18.0	14.0	22.9	47.8	42.8	52.9	415	6.2	4.8	7.9	1205
DOM	Dominican Republic	DHS 1999	LMI	LAC	15.1	0.0	0.0	61.8	0.0	0.0	52	6.2	0.0	0.0	140

ISO	Country	Survey and year	Income group*	Region of the world**	Exclusive breastfeeding under six months			Formula consumption under six months			Number of children	Formula consumption between 6-23 months			Number of children
					Prevalence	95% CI		Prevalence	95% CI			Prevalence	95% CI		
DOM	Dominican Republic	DHS 2002	LMI	LAC	10.4	7.9	13.6	44.6	39.3	50.0	1003	4.8	3.8	6.1	3098
DOM	Dominican Republic	DHS 2007	LMI	LAC	7.8	5.8	10.5	74.5	69.8	78.8	986	82.3	80.1	84.3	2915
DOM	Dominican Republic	DHS 2013	UMI	LAC	6.7	3.9	11.4	35.6	28.8	43.2	307	9.6	6.5	13.8	1048
DOM	Dominican Republic	MICS 2014	UMI	LAC	4.7	3.4	6.5	30.0	26.1	34.2	1656	10.7	9.4	12.2	6235
EGY	Egypt	DHS 1995	LMI	MENA	56.3	52.8	59.8	7.0	5.2	9.3	1138	9.2	8.1	10.5	3182
EGY	Egypt	DHS 2000	LMI	MENA	56.2	53.1	59.2	6.0	4.7	7.8	1201	13.1	11.8	14.5	3127
EGY	Egypt	DHS 2005	LMI	MENA	39.0	33.5	44.7	5.2	3.3	8.2	1250	5.3	4.1	6.9	3891
EGY	Egypt	DHS 2008	LMI	MENA	53.2	49.9	56.4	4.8	3.5	6.4	1130	4.4	3.7	5.3	3324
EGY	Egypt	DHS 2014	LMI	MENA	39.7	36.7	42.7	15.6	13.4	18.0	1487	4.7	4.0	5.4	4834
SWZ	Eswatini	DHS 2006	LMI	ESA	32.3	26.2	39.1	26.3	21.0	32.4	258	18.6	16.0	21.6	767
SWZ	Eswatini	MICS 2010	LMI	ESA	44.1	38.3	50.1	25.2	20.3	30.8	269	19.8	17.0	22.8	773
SWZ	Eswatini	MICS 2014	LMI	ESA	63.8	55.2	71.6	15.1	10.0	22.1	235	20.3	17.2	23.9	789
ETH	Ethiopia	DHS 2000	LI	ESA	54.5	49.9	59.1	***	***	***	966	***	***	***	***
ETH	Ethiopia	DHS 2005	LI	ESA	49.0	45.3	52.7	***	***	***	1008	***	***	***	***
ETH	Ethiopia	DHS 2011	LI	ESA	52.0	47.3	56.7	2.6	1.5	4.5	1187	1.5	1.0	2.2	2850
ETH	Ethiopia	DHS 2016	LI	ESA	57.5	52.7	62.1	0.9	0.4	2.0	1092	1.6	1.0	2.5	2822
GAB	Gabon	DHS 2000	UMI	WCA	5.4	3.3	8.6	26.3	22.1	30.9	451	28.4	25.5	31.5	1183
GAB	Gabon	DHS 2012	UMI	WCA	6.0	3.7	9.7	65.3	59.5	70.6	631	34.5	30.4	38.8	1719
GMB	Gambia	MICS 2005	LI	WCA	40.8	37.0	44.6	9.8	7.9	12.2	855	16.0	14.0	18.1	2177
GMB	Gambia	MICS 2010	LI	WCA	33.5	30.3	37.0	13.2	10.7	16.1	1412	12.7	11.2	14.5	3790
GMB	Gambia	DHS 2013	LI	WCA	46.8	41.8	51.8	2.2	1.3	3.8	951	4.6	3.2	6.4	2424
GMB	Gambia	MICS 2018	LI	WCA	55.2	50.7	59.6	5.4	3.6	7.9	910	3.6	2.6	4.9	2724
GEO	Georgia	MICS 2005	LMI	ECA	11.8	7.0	19.3	46.7	38.6	55.0	171	32.9	28.3	37.8	577
GEO	Georgia	MICS 2018	UMI	ECA	21.2	15.7	28.0	45.5	36.1	55.2	234	26.1	21.5	31.3	701
GHA	Ghana	DHS 1993	LI	WCA	5.8	3.7	8.9	7.1	4.9	10.3	379	5.3	4.0	6.8	968
GHA	Ghana	DHS 1998	LI	WCA	31.5	26.2	37.4	7.4	4.7	11.6	302	11.0	9.0	13.3	950
GHA	Ghana	DHS 2003	LI	WCA	53.4	47.0	59.7	3.5	1.8	6.7	338	7.8	6.0	9.9	1102
GHA	Ghana	MICS 2006	LI	WCA	55.3	48.9	61.6	8.1	5.4	12.0	384	10.7	8.0	14.2	1043

ISO	Country	Survey and year	Income group[*]	Region of the world[**]	Exclusive breastfeeding under six months			Formula consumption under six months			Number of children	Formula consumption between 6-23 months			Number of children
					Prevalence	95% CI		Prevalence	95% CI			Prevalence	95% CI		
GHA	Ghana	DHS 2008	LI	WCA	62.8	57.0	68.3	5.7	3.5	9.3	318	9.4	7.3	12.2	857
GHA	Ghana	MICS 2011	LMI	WCA	45.7	40.6	50.8	11.7	8.7	15.6	792	5.2	3.6	7.3	2171
GHA	Ghana	DHS 2014	LMI	WCA	52.3	47.2	57.3	9.7	6.9	13.5	606	4.7	3.6	6.2	1656
GHA	Ghana	MICS 2017	LMI	WCA	42.9	38.4	47.5	17.8	14.1	22.3	891	5.8	4.5	7.5	2585
GTM	Guatemala	DHS 1995	LMI	LAC	46.4	41.8	51.0	10.0	8.0	12.6	969	6.6	5.2	8.3	2820
GTM	Guatemala	DHS 1998	LMI	LAC	39.2	30.9	48.3	14.0	9.8	19.7	476	3.7	2.5	5.4	1324
GTM	Guatemala	RHS 2002	LMI	LAC	50.8	45.4	56.2	12.9	9.3	17.6	683	5.0	3.8	6.5	2157
GTM	Guatemala	RHS 2008	LMI	LAC	49.9	46.1	53.7	18.9	15.9	22.3	1084	11.5	10.0	13.1	3003
GTM	Guatemala	DHS 2014	LMI	LAC	53.2	49.7	56.7	20.9	18.4	23.5	1175	7.8	6.8	9.0	3509
GIN	Guinea	DHS 1999	LI	WCA	11.3	8.5	14.9	3.8	2.5	5.9	662	3.5	2.6	4.7	1321
GIN	Guinea	DHS 2005	LI	WCA	27.0	23.0	31.4	5.2	3.7	7.1	733	16.1	14.0	18.4	1650
GIN	Guinea	DHS 2012	LI	WCA	20.5	16.4	25.3	6.7	4.7	9.4	718	5.2	4.1	6.6	1953
GIN	Guinea	MICS 2016	LI	WCA	35.2	30.6	40.1	7.2	5.2	9.9	686	8.8	7.3	10.6	2122
GIN	Guinea	DHS 2018	LI	WCA	33.4	29.8	37.3	7.9	6.0	10.3	916	10.1	8.5	12.1	1909
GNB	Guinea Bissau	MICS 2006	LI	WCA	16.3	12.9	20.2	5.2	3.7	7.3	658	15.1	13.0	17.6	1837
GNB	Guinea Bissau	MICS 2014	LI	WCA	52.5	48.5	56.6	1.8	0.9	3.5	830	11.5	9.7	13.5	2268
GUY	Guyana	MICS 2006	LMI	LAC	21.4	15.9	28.3	43.0	35.1	51.3	219	35.6	30.7	40.8	716
GUY	Guyana	DHS 2009	LMI	LAC	33.2	25.8	41.7	28.4	20.4	38.0	230	38.8	33.2	44.7	607
GUY	Guyana	MICS 2014	LMI	LAC	23.3	17.9	29.6	45.3	37.4	53.5	290	24.1	20.6	28.1	1034
HTI	Haiti	DHS 1994	LI	LAC	2.5	1.3	5.0	17.7	13.6	22.7	317	5.5	4.2	7.1	931
HTI	Haiti	DHS 2000	LI	LAC	23.7	17.1	31.8	22.7	17.7	28.7	563	13.5	10.5	17.2	1797
HTI	Haiti	DHS 2005	LI	LAC	40.7	35.2	46.4	14.0	9.7	19.9	598	7.3	5.8	9.1	1691
HTI	Haiti	DHS 2012	LI	LAC	39.7	35.2	44.5	18.5	15.2	22.4	726	15.9	13.7	18.5	1972
HTI	Haiti	DHS 2016	LI	LAC	39.9	35.1	44.9	18.9	15.6	22.6	700	15.6	13.4	18.1	1652
HND	Honduras	RHS 1991	LI	LAC	29.1	25.4	33.1	***	***	***	571	***	***	***	***
HND	Honduras	RHS 1996	LI	LAC	38.5	32.0	45.5	***	***	***	348	***	***	***	***
HND	Honduras	RHS 2001	LMI	LAC	37.5	32.4	42.8	***	***	***	434	***	***	***	***
HND	Honduras	DHS 2005	LMI	LAC	29.7	26.4	33.2	34.5	30.6	38.5	973	19.4	17.4	21.5	3066

ISO	Country	Survey and year	Income group*	Region of the world**	Exclusive breastfeeding under six months			Formula consumption under six months			Number of children	Formula consumption between 6-23 months			Number of children
					Prevalence	95% CI		Prevalence	95% CI			Prevalence	95% CI		
HND	Honduras	DHS 2011	LMI	LAC	31.2	28.3	34.3	26.1	22.8	29.7	1084	4.5	3.6	5.6	3237
IND	India	DHS 1998	LI	SA	46.8	44.7	48.8	***	***	***	5503	***	***	***	***
IND	India	DHS 2005	LI	SA	46.4	44.4	48.4	4.0	3.3	4.7	4616	10.2	9.5	11.0	13904
IND	India	DHS 2015	LMI	SA	54.9	54.0	55.8	3.7	3.3	4.1	22626	10.6	10.2	11.0	71762
IDN	Indonesia	DHS 1994	LMI	EAP	37.4	33.6	41.3	***	***	***	1639	***	***	***	***
IDN	Indonesia	DHS 1997	LMI	EAP	43.0	39.1	47.0	***	***	***	1678	***	***	***	***
IDN	Indonesia	DHS 2002	LI	EAP	39.5	35.5	43.7	21.3	18.3	24.7	1641	32.2	29.8	34.8	4413
IDN	Indonesia	DHS 2007	LMI	EAP	32.4	28.8	36.3	34.8	31.1	38.7	1802	38.3	36.1	40.5	5209
IDN	Indonesia	DHS 2012	LMI	EAP	41.5	38.0	45.0	31.7	28.5	35.1	1686	37.5	35.3	39.7	5193
IDN	Indonesia	DHS 2017	LMI	EAP	51.5	48.4	54.5	28.9	26.1	31.8	1666	36.9	35.2	38.6	5033
IRQ	Iraq	MICS 2006	LMI	MENA	25.5	22.8	28.4	25.9	23.1	28.8	1624	26.9	25.3	28.6	5352
IRQ	Iraq	MICS 2011	LMI	MENA	19.6	17.7	21.6	43.5	40.9	46.1	3882	35.5	34.0	37.1	11168
IRQ	Iraq	MICS 2018	UMI	MENA	26.7	24.0	29.5	44.5	41.3	47.6	1681	45.8	42.7	49.1	4786
JAM	Jamaica	MICS 2005	LMI	LAC	15.2	9.6	23.1	63.1	53.7	71.6	129	62.5	57.3	67.4	412
JAM	Jamaica	MICS 2011	UMI	LAC	23.8	17.5	31.4	52.9	44.0	61.5	167	58.2	52.0	64.1	476
JOR	Jordan	DHS 1997	LMI	MENA	11.0	8.4	14.3	30.9	27.0	35.0	499	9.0	7.6	10.5	1767
JOR	Jordan	DHS 2002	LMI	MENA	26.7	22.4	31.5	31.4	26.9	36.3	507	12.0	10.2	14.2	1728
JOR	Jordan	DHS 2007	LMI	MENA	21.8	18.2	25.9	48.6	43.3	54.0	1117	41.4	38.3	44.6	2739
JOR	Jordan	DHS 2012	UMI	MENA	22.7	18.2	27.9	6.3	3.8	10.2	850	8.7	6.9	10.8	2783
JOR	Jordan	DHS 2017	UMI	MENA	25.5	22.0	29.5	49.5	45.2	53.8	1218	46.8	43.8	49.9	2680
KAZ	Kazakhstan	DHS 1995	LMI	ECA	9.6	5.6	15.9	18.7	10.8	30.4	118	10.6	7.5	14.9	403
KAZ	Kazakhstan	DHS 1999	LMI	ECA	35.8	26.1	46.7	8.2	3.8	17.0	99	6.8	4.3	10.4	349
KAZ	Kazakhstan	MICS 2006	UMI	ECA	16.8	13.1	21.2	22.9	18.3	28.3	387	31.2	28.1	34.4	1437
KAZ	Kazakhstan	MICS 2010	UMI	ECA	31.8	27.5	36.4	25.6	21.9	29.8	543	31.3	28.7	34.0	1582
KAZ	Kazakhstan	MICS 2015	UMI	ECA	37.8	32.0	43.9	20.0	15.6	25.2	508	12.8	10.9	15.0	1632
KEN	Kenya	DHS 1993	LI	ESA	11.3	8.2	15.3	2.2	1.3	3.7	518	3.8	2.8	5.0	1645
KEN	Kenya	DHS 1998	LI	ESA	12.6	9.6	16.4	4.2	2.6	6.7	518	3.4	2.4	4.8	1624
KEN	Kenya	DHS 2003	LI	ESA	12.7	9.8	16.2	4.8	3.1	7.5	599	4.3	3.4	5.6	1610

ISO	Country	Survey and year	Income group[*]	Region of the world[**]	Exclusive breastfeeding under six months			Formula consumption under six months			Number of children	Formula consumption between 6-23 months			Number of children
					Prevalence	95% CI		Prevalence	95% CI			Prevalence	95% CI		
KEN	Kenya	DHS 2008	LI	ESA	31.9	26.7	37.7	2.5	1.3	4.7	587	3.0	2.0	4.4	1656
KEN	Kenya	DHS 2014	LMI	ESA	61.4	57.2	65.4	0.3	0.1	0.7	856	2.7	2.1	3.5	5792
KGZ	Kyrgyzstan	DHS 1997	LI	ECA	24.0	17.0	32.9	14.1	8.7	21.9	177	24.9	20.4	30.0	521
KGZ	Kyrgyzstan	MICS 2005	LI	ECA	35.6	27.3	44.9	22.6	15.9	30.9	286	27.6	22.8	33.1	874
KGZ	Kyrgyzstan	DHS 2012	LI	ECA	56.1	50.6	61.5	6.7	4.5	10.0	447	8.8	7.0	11.0	1317
KGZ	Kyrgyzstan	MICS 2014	LMI	ECA	41.1	35.4	47.1	7.9	5.3	11.6	432	11.2	9.3	13.5	1402
KGZ	Kyrgyzstan	MICS 2018	LMI	ECA	46.3	39.9	52.7	13.6	9.8	18.6	390	17.0	14.1	20.4	992
LAO	Lao	MICS 2006	LI	EAP	26.7	21.5	32.6	6.3	4.0	10.0	445	5.6	3.9	8.1	1201
LAO	Lao	MICS 2011	LMI	EAP	40.4	37.2	43.8	9.7	7.8	12.1	1168	21.4	19.6	23.4	3265
LAO	Lao	MICS 2017	LMI	EAP	44.9	41.3	48.6	15.9	13.2	19.0	1134	19.7	17.8	21.7	3428
LSO	Lesotho	DHS 2004	LI	ESA	36.4	31.3	41.8	7.1	4.9	10.2	399	15.1	13.0	17.5	982
LSO	Lesotho	DHS 2009	LMI	ESA	53.5	48.2	58.7	16.0	12.5	20.2	434	11.3	8.8	14.4	1118
LSO	Lesotho	DHS 2014	LMI	ESA	66.9	60.5	72.6	17.4	12.0	24.4	327	13.3	10.5	16.6	948
LSO	Lesotho	MICS 2018	LMI	ESA	59.0	50.6	67.0	20.0	14.0	27.7	215	16.3	13.7	19.4	1009
LBR	Liberia	DHS 2007	LI	WCA	29.1	24.9	33.8	9.2	5.9	14.2	504	7.8	6.2	9.8	1519
LBR	Liberia	DHS 2013	LI	WCA	55.2	49.6	60.7	5.1	3.1	8.2	718	5.7	3.8	8.6	2155
MDG	Madagascar	DHS 1997	LI	ESA	48.0	43.9	52.1	1.3	0.6	2.5	628	2.6	1.9	3.6	1638
MDG	Madagascar	DHS 2003	LI	ESA	67.2	62.2	71.9	0.2	0.1	0.7	499	4.8	3.1	7.3	1538
MDG	Madagascar	DHS 2008	LI	ESA	50.7	46.6	54.9	5.1	3.7	6.9	1214	10.7	9.3	12.3	3306
MDG	Madagascar	MICS 2018	LI	ESA	50.6	47.4	53.8	2.7	1.9	4.0	1386	1.8	1.2	2.8	3859
MWI	Malawi	DHS 2000	LI	ESA	44.0	40.8	47.3	1.7	1.1	2.5	1242	3.3	2.6	4.3	3312
MWI	Malawi	DHS 2004	LI	ESA	52.8	49.2	56.3	3.0	2.0	4.5	1099	4.7	3.6	6.3	3327
MWI	Malawi	MICS 2006	LI	ESA	56.8	53.5	60.1	0.6	0.3	1.2	2298	1.1	0.8	1.5	7668
MWI	Malawi	DHS 2010	LI	ESA	71.4	68.0	74.6	1.4	0.8	2.4	1663	2.8	2.2	3.6	5688
MWI	Malawi	MICS 2013	LI	ESA	70.2	66.4	73.8	2.0	1.1	3.7	1686	2.1	1.6	2.7	5661
MWI	Malawi	DHS 2015	LI	ESA	61.0	57.9	64.0	1.9	1.1	3.4	1636	2.6	1.9	3.4	4747
MDV	Maldives	DHS 2009	LMI	SA	47.8	41.8	53.9	23.0	18.0	29.0	414	47.4	43.9	50.9	1256
MDV	Maldives	DHS 2016	UMI	SA	63.5	55.4	70.9	17.0	10.2	26.9	288	44.9	39.6	50.3	848

ISO	Country	Survey and year	Income group[*]	Region of the world[**]	Exclusive breastfeeding under six months			Formula consumption under six months			Number of children	Formula consumption between 6-23 months			Number of children
					Prevalence	95% CI		Prevalence	95% CI			Prevalence	95% CI		
MLI	Mali	DHS 1995	LI	WCA	8.4	6.5	10.7	3.4	2.4	4.9	1036	6.6	5.3	8.2	2475
MLI	Mali	DHS 2001	LI	WCA	25.1	22.1	28.3	3.8	2.8	5.1	1418	12.6	11.0	14.3	3372
MLI	Mali	DHS 2006	LI	WCA	37.8	34.4	41.3	3.2	2.4	4.4	1421	11.2	9.8	12.8	3780
MLI	Mali	MICS 2009	LI	WCA	20.5	18.6	22.6	3.0	2.3	3.9	2913	7.6	6.6	8.6	7816
MLI	Mali	DHS 2012	LI	WCA	32.8	29.0	36.8	4.7	3.4	6.5	999	6.0	4.7	7.6	2795
MLI	Mali	MICS 2015	LI	WCA	32.6	29.6	35.6	1.5	1.0	2.2	1663	5.2	4.4	6.2	4865
MLI	Mali	DHS 2018	LI	WCA	40.4	36.6	44.2	5.2	3.7	7.2	997	6.3	5.3	7.4	2713
MRT	Mauritania	MICS 2007	LI	WCA	11.5	9.2	14.2	15.9	13.0	19.3	915	16.4	14.1	18.9	2693
MRT	Mauritania	MICS 2011	LI	WCA	26.9	23.6	30.4	11.3	9.1	13.8	995	16.7	15.1	18.4	2749
MRT	Mauritania	MICS 2015	LMI	WCA	41.4	37.7	45.2	10.7	8.2	13.7	915	10.6	8.7	12.7	3184
MDA	Moldova	DHS 2005	LMI	ECA	45.5	36.7	54.7	23.4	16.6	32.0	158	30.5	26.4	34.9	486
MDA	Moldova	MICS 2012	LMI	ECA	36.4	28.9	44.5	21.2	15.0	29.1	176	24.8	21.3	28.6	591
MNG	Mongolia	MICS 2005	LI	EAP	57.2	51.8	62.4	15.5	11.8	20.2	399	31.6	27.8	35.5	1098
MNG	Mongolia	MICS 2010	LMI	EAP	65.7	59.8	71.1	8.1	5.3	12.1	410	6.5	5.0	8.5	1323
MNG	Mongolia	MICS 2013	LMI	EAP	47.1	42.9	51.5	17.4	14.3	21.2	644	6.8	5.6	8.2	1802
MNG	Mongolia	MICS 2018	LMI	EAP	51.5	45.2	57.8	21.2	16.4	26.9	616	8.2	6.1	10.8	1674
MNE	Montenegro	MICS 2005	UMI	ECA	20.1	12.2	31.3	25.3	16.1	37.4	71	9.1	5.6	14.5	284
MNE	Montenegro	MICS 2013	UMI	ECA	16.8	10.2	26.4	36.0	24.0	50.1	108	6.9	4.7	9.9	388
MNE	Montenegro	MICS 2018	UMI	ECA	20.5	13.3	30.3	38.1	25.6	52.4	101	21.3	15.0	29.2	332
MOZ	Mozambique	DHS 1997	LI	ESA	30.3	23.0	38.9	***	***	***	706	***	***	***	***
MOZ	Mozambique	DHS 2003	LI	ESA	30.0	26.6	33.7	5.2	3.8	7.1	1027	5.4	4.5	6.5	2805
MOZ	Mozambique	MICS 2008	LI	ESA	37.2	33.4	41.1	5.9	4.5	7.7	1229	4.1	3.3	5.1	3652
MOZ	Mozambique	DHS 2011	LI	ESA	41.1	37.1	45.3	3.8	2.7	5.5	1044	1.5	1.1	2.0	3280
MOZ	Mozambique	DHS 2015	LI	ESA	54.6	48.5	60.6	***	***	***	514	***	***	***	***
NAM	Namibia	DHS 2000	LMI	ESA	18.5	12.9	25.8	***	***	***	405	***	***	***	***
NAM	Namibia	DHS 2006	LMI	ESA	23.9	19.6	28.8	18.5	14.8	22.9	495	17.9	15.2	20.9	1396
NAM	Namibia	DHS 2013	UMI	ESA	48.5	43.6	53.4	15.6	12.3	19.6	525	11.7	9.8	13.9	1303
NPL	Nepal	DHS 1996	LI	SA	74.9	71.2	78.2	1.0	0.5	2.2	677	1.1	0.7	1.8	2038

ISO	Country	Survey and year	Income group*	Region of the world**	Exclusive breastfeeding under six months			Formula consumption under six months			Number of children	Formula consumption between 6-23 months			Number of children
					Prevalence	95% CI		Prevalence	95% CI			Prevalence	95% CI		
NPL	Nepal	DHS 2001	LI	SA	68.3	63.9	72.5	***	***	***	634	***	***	***	***
NPL	Nepal	DHS 2006	LI	SA	53.0	46.7	59.3	2.0	0.8	4.7	473	2.0	1.2	3.4	1542
NPL	Nepal	DHS 2011	LI	SA	69.6	63.6	75.1	1.1	0.6	2.3	497	2.3	1.5	3.5	1421
NPL	Nepal	MICS 2014	LI	SA	56.9	50.3	63.4	2.7	1.4	5.1	452	3.1	2.2	4.5	1556
NPL	Nepal	DHS 2016	LI	SA	66.1	60.8	71.0	4.5	2.6	7.9	467	2.5	1.8	3.6	1463
NER	Niger	DHS 1998	LI	WCA	0.8	0.4	1.6	1.7	1.1	2.6	816	1.6	1.2	2.2	2063
NER	Niger	DHS 2006	LI	WCA	13.5	11.3	16.0	9.5	7.5	11.9	945	26.8	24.9	28.8	2479
NER	Niger	DHS 2012	LI	WCA	23.3	19.8	27.1	1.2	0.7	2.0	1303	1.6	1.2	2.1	3260
NGA	Nigeria	DHS 1999	LI	WCA	15.5	12.2	19.4	7.7	5.7	10.4	555	5.2	4.0	6.8	1620
NGA	Nigeria	DHS 2003	LI	WCA	17.2	13.3	22.0	12.7	9.0	17.7	611	10.2	8.4	12.3	1586
NGA	Nigeria	MICS 2007	LI	WCA	11.7	9.8	14.0	14.5	12.2	17.1	1729	12.4	10.9	14.2	4788
NGA	Nigeria	DHS 2008	LMI	WCA	13.1	11.5	14.8	8.6	7.4	10.0	2889	6.4	5.6	7.2	7557
NGA	Nigeria	MICS 2011	LMI	WCA	15.1	13.2	17.2	14.7	12.9	16.8	2714	14.4	12.9	16.1	7528
NGA	Nigeria	DHS 2013	LMI	WCA	17.4	15.6	19.4	5.0	4.1	6.1	2934	5.4	4.8	6.1	8721
NGA	Nigeria	MICS 2016	LMI	WCA	23.7	21.4	26.3	6.4	5.3	7.6	2748	6.6	6.0	7.4	8219
NGA	Nigeria	DHS 2018	LMI	WCA	28.7	26.8	30.6	5.9	4.9	7.0	3193	6.5	5.8	7.3	8883
MKD	North Macedonia	MICS 2005	LMI	ECA	16.2	6.0	36.7	22.8	11.0	41.3	233	16.1	10.7	23.6	1199
MKD	North Macedonia	MICS 2011	UMI	ECA	23.0	14.9	33.7	40.3	28.3	53.6	112	23.6	18.7	29.3	410
PAK	Pakistan	DHS 2006	LI	SA	37.1	33.4	40.9	***	***	***	947	***	***	***	***
PAK	Pakistan	DHS 2012	LMI	SA	37.7	33.7	42.0	9.9	7.6	12.9	1076	5.9	4.8	7.3	2870
PAK	Pakistan	DHS 2017	LMI	SA	47.5	43.3	51.6	12.2	9.7	15.1	1117	6.7	5.5	8.1	2566
PER	Peru	DHS 1996	LMI	LAC	53.0	49.6	56.4	9.4	7.4	11.8	1519	3.0	2.3	3.8	4729
PER	Peru	DHS 2000	LMI	LAC	67.2	63.5	70.7	8.8	6.7	11.5	1162	2.2	1.6	3.0	3699
PER	Peru	DHS 2004	LMI	LAC	60.5	51.9	68.4	13.9	8.4	22.1	233	3.8	2.3	6.3	722
PER	Peru	DHS 2005	LMI	LAC	64.5	57.0	71.4	14.3	9.3	21.3	273	4.6	2.7	7.9	801
PER	Peru	DHS 2006	LMI	LAC	62.7	53.7	70.9	12.3	7.4	19.6	251	1.9	0.9	3.8	842
PER	Peru	DHS 2007	LMI	LAC	68.7	58.7	77.3	13.7	6.2	27.4	223	5.3	3.1	8.9	752
PER	Peru	DHS 2008	UMI	LAC	65.7	58.4	72.3	16.9	11.9	23.5	545	6.1	4.4	8.2	1834

ISO	Country	Survey and year	Income group*	Region of the world**	Exclusive breastfeeding under six months			Formula consumption under six months			Number of children	Formula consumption between 6-23 months			Number of children
					Prevalence	95% CI		Prevalence	95% CI			Prevalence	95% CI		
PER	Peru	DHS 2009	UMI	LAC	68.5	64.4	72.4	13.7	10.7	17.3	901	8.9	7.3	10.7	3027
PER	Peru	DHS 2010	UMI	LAC	68.3	64.0	72.3	15.4	12.1	19.4	766	8.4	6.8	10.2	2706
PER	Peru	DHS 2011	UMI	LAC	70.6	65.7	75.1	14.9	11.6	18.9	780	9.0	7.5	10.8	2557
PER	Peru	DHS 2012	UMI	LAC	67.6	62.9	71.9	20.0	16.3	24.1	827	11.7	9.9	13.9	2729
PER	Peru	DHS 2013	UMI	LAC	72.3	67.2	76.9	17.8	14.1	22.1	750	14.8	12.7	17.1	2580
PER	Peru	DHS 2014	UMI	LAC	68.4	64.2	72.4	22.3	18.6	26.4	878	15.9	14.1	17.9	2767
PER	Peru	DHS 2015	UMI	LAC	64.9	61.7	67.9	23.6	20.8	26.5	1847	17.9	16.6	19.3	7040
PER	Peru	DHS 2016	UMI	LAC	69.8	66.7	72.8	19.0	16.5	21.9	1446	19.1	17.6	20.7	6198
PER	Peru	DHS 2017	UMI	LAC	64.3	61.1	67.3	21.7	19.0	24.5	1792	20.5	19.2	22.0	6580
PER	Peru	DHS 2018	UMI	LAC	66.5	63.5	69.3	20.7	18.3	23.3	1737	19.4	18.1	20.8	6534
PHL	Philippines	DHS 1993	LMI	EAP	26.1	23.0	29.5	20.5	17.7	23.7	821	12.3	10.9	13.7	2481
PHL	Philippines	DHS 1998	LMI	EAP	37.5	34.0	41.2	40.2	36.3	44.2	757	34.9	32.4	37.5	2137
PHL	Philippines	DHS 2003	LMI	EAP	33.5	29.8	37.5	29.8	26.1	33.9	624	49.6	47.1	52.1	1940
PHL	Philippines	DHS 2008	LMI	EAP	34.0	30.1	38.2	36.3	32.0	40.9	593	37.1	34.4	39.8	1818
RWA	Rwanda	DHS 2000	LI	ESA	83.3	80.2	86.1	0.6	0.3	1.4	757	1.5	1.1	2.2	2115
RWA	Rwanda	DHS 2005	LI	ESA	88.4	85.8	90.6	4.1	2.9	5.8	876	59.2	56.9	61.5	2340
RWA	Rwanda	DHS 2010	LI	ESA	84.9	81.9	87.5	0.4	0.2	1.1	709	0.9	0.6	1.3	2333
RWA	Rwanda	DHS 2014	LI	ESA	87.3	84.5	89.6	0.6	0.2	1.4	703	1.2	0.9	1.8	2354
STP	Sao Tome and Principe	DHS 2008	LMI	WCA	51.4	43.3	59.4	7.1	3.8	12.8	192	37.8	32.6	43.3	563
STP	Sao Tome and Principe	MICS 2014	LMI	WCA	73.8	65.8	80.6	4.5	2.0	9.6	169	7.3	4.8	10.9	571
SEN	Senegal	DHS 1997	LI	WCA	10.6	8.3	13.5	***	***	***	817	***	***	***	***
SEN	Senegal	DHS 2005	LI	WCA	34.1	30.3	38.0	8.7	6.3	11.8	1290	18.6	16.3	21.2	3060
SEN	Senegal	DHS 2010	LMI	WCA	39.0	35.7	42.3	3.9	2.6	5.7	1334	4.0	3.1	5.1	3375
SEN	Senegal	DHS 2012	LMI	WCA	37.0	32.9	41.3	2.2	1.1	4.5	672	2.8	1.8	4.4	1935
SEN	Senegal	DHS 2014	LMI	WCA	32.4	27.2	38.0	2.7	1.2	6.0	612	1.2	0.6	2.5	1885
SEN	Senegal	DHS 2015	LI	WCA	33.3	27.9	39.2	3.3	1.7	6.3	626	2.2	1.4	3.5	1984
SEN	Senegal	DHS 2016	LI	WCA	36.4	31.1	42.2	2.0	0.8	4.9	605	3.0	1.9	4.6	1940
SEN	Senegal	DHS 2017	LI	WCA	42.1	38.6	45.7	5.3	3.7	7.6	1142	4.2	3.1	5.7	3489

ISO	Country	Survey and year	Income group*	Region of the world**	Exclusive breastfeeding under six months			Formula consumption under six months			Number of children	Formula consumption between 6-23 months			Number of children
					Prevalence	95% CI		Prevalence	95% CI			Prevalence	95% CI		
SRB	Serbia	MICS 2005	UMI	ECA	15.3	11.2	20.7	21.3	16.1	27.7	334	10.7	8.3	13.7	1129
SRB	Serbia	MICS 2010	UMI	ECA	13.7	8.9	20.4	40.2	30.4	50.8	246	9.4	6.7	13.2	1011
SRB	Serbia	MICS 2014	UMI	ECA	12.8	5.8	26.2	40.9	29.4	53.6	169	8.9	7.0	11.2	795
SLE	Sierra Leone	MICS 2005	LI	WCA	8.6	6.3	11.5	10.4	8.0	13.5	508	11.4	9.6	13.4	1592
SLE	Sierra Leone	DHS 2008	LI	WCA	11.2	8.6	14.5	9.4	7.3	12.1	617	10.7	9.0	12.8	1537
SLE	Sierra Leone	MICS 2010	LI	WCA	31.5	27.9	35.4	10.1	8.1	12.6	831	13.2	11.6	15.1	2442
SLE	Sierra Leone	DHS 2013	LI	WCA	32.0	28.1	36.3	5.9	4.5	7.8	1115	5.5	4.4	6.8	3088
SLE	Sierra Leone	MICS 2017	LI	WCA	52.2	48.9	55.5	8.9	6.8	11.5	1170	10.4	8.9	12.0	3411
ZAF	South Africa	DHS 1998	LMI	ESA	7.0	4.9	9.8	51.5	46.6	56.3	505	39.8	36.7	42.9	1374
ZAF	South Africa	DHS 2016	UMI	ESA	31.6	25.6	38.3	31.4	25.9	37.5	346	40.2	35.8	44.8	877
SUR	Suriname	MICS 2006	LMI	LAC	2.2	0.8	5.7	60.4	52.6	67.7	182	59.4	54.7	63.9	659
SUR	Suriname	MICS 2010	UMI	LAC	2.8	1.4	5.5	74.1	68.3	79.1	304	72.7	69.1	76.0	1062
SUR	Suriname	MICS 2018	UMI	LAC	8.9	5.7	13.7	58.9	51.4	66.1	335	64.9	60.5	69.1	1182
TJK	Tajikistan	MICS 2005	LI	ECA	25.4	19.7	32.1	6.8	4.4	10.4	388	17.7	15.1	20.6	1275
TJK	Tajikistan	DHS 2012	LI	ECA	34.3	29.2	39.8	11.5	8.7	15.1	424	17.7	15.3	20.4	1467
TJK	Tajikistan	DHS 2017	LI	ECA	35.8	30.9	41.1	9.7	7.4	12.5	553	11.7	10.0	13.6	1722
TZA	Tanzania	DHS 1996	LI	ESA	29.4	25.9	33.2	3.6	2.3	5.6	654	10.3	8.9	11.8	1920
TZA	Tanzania	DHS 1999	LI	ESA	31.8	24.2	40.6	***	***	***	322	***	***	***	***
TZA	Tanzania	DHS 2004	LI	ESA	41.3	37.2	45.5	0.8	0.4	1.8	825	4.1	3.2	5.3	2408
TZA	Tanzania	DHS 2010	LI	ESA	49.8	45.6	54.0	1.0	0.4	2.2	803	0.7	0.4	1.5	2235
TZA	Tanzania	DHS 2015	LI	ESA	59.2	55.7	62.7	0.7	0.3	1.7	1015	1.1	0.7	1.7	3020
THA	Thailand	MICS 2005	LMI	EAP	5.4	3.5	8.1	28.2	24.0	32.8	873	28.3	25.7	31.1	2913
THA	Thailand	MICS 2012	UMI	EAP	12.3	9.0	16.5	45.0	38.9	51.3	591	63.0	59.1	66.8	2618
THA	Thailand	MICS 2015	UMI	EAP	23.1	16.9	30.8	44.9	37.1	52.9	661	53.8	49.6	58.0	3222
TLS	Timor-Leste	DHS 2009	LMI	EAP	51.5	47.7	55.2	22.1	18.6	26.0	960	20.0	18.0	22.2	2570
TLS	Timor-Leste	DHS 2016	LMI	EAP	50.7	45.8	55.5	9.5	7.0	12.7	743	11.9	10.2	13.7	1950
TGO	Togo	DHS 1998	LI	WCA	10.5	7.7	14.2	9.7	7.1	13.0	692	10.4	8.8	12.3	1878
TGO	Togo	MICS 2006	LI	WCA	28.8	23.3	35.0	18.8	15.1	23.1	447	58.2	54.4	62.0	1341

ISO	Country	Survey and year	Income group[*]	Region of the world[**]	Exclusive breastfeeding under six months			Formula consumption under six months			Number of children	Formula consumption between 6-23 months			Number of children
					Prevalence	95% CI		Prevalence	95% CI			Prevalence	95% CI		
TGO	Togo	MICS 2010	LI	WCA	62.4	57.3	67.3	4.9	2.8	8.5	563	11.1	9.4	13.2	1392
TGO	Togo	DHS 2013	LI	WCA	57.5	52.7	62.1	2.1	1.1	3.9	603	2.3	1.6	3.3	2070
TGO	Togo	MICS 2017	LI	WCA	65.4	59.5	70.9	2.7	1.5	4.8	504	2.1	1.4	3.0	1461
TUN	Tunisia	MICS 2011	UMI	MENA	8.5	5.5	12.9	9.5	6.1	14.4	306	12.8	10.1	16.0	854
TUN	Tunisia	MICS 2018	LMI	MENA	13.8	10.2	18.5	51.5	45.2	57.8	299	13.1	10.9	15.8	946
TUR	Turkey	DHS 1993	LMI	ECA	10.5	7.5	14.4	18.6	14.8	23.0	362	5.5	4.3	7.1	1034
TUR	Turkey	DHS 1998	UMI	ECA	7.1	4.4	11.5	26.8	22.0	32.2	370	13.4	10.9	16.4	1012
TUR	Turkey	DHS 2003	LMI	ECA	20.8	16.4	26.1	22.0	17.7	27.0	413	16.3	13.8	19.2	1156
TUR	Turkey	DHS 2013	UMI	ECA	30.7	24.2	38.1	31.8	26.4	37.8	332	27.1	24.2	30.3	1053
TKM	Turkmenistan	MICS 2006	LMI	ECA	10.9	7.0	16.4	14.0	9.8	19.6	237	33.6	28.3	39.3	625
TKM	Turkmenistan	MICS 2015	UMI	ECA	58.9	53.2	64.4	12.3	8.9	16.9	342	9.5	7.7	11.5	1169
UGA	Uganda	DHS 1995	LI	ESA	57.4	52.6	62.1	1.9	0.8	4.3	629	2.1	1.6	2.8	2079
UGA	Uganda	DHS 2000	LI	ESA	63.2	58.3	67.9	1.5	0.8	2.7	656	1.9	1.4	2.8	1994
UGA	Uganda	DHS 2006	LI	ESA	60.1	56.3	63.7	1.2	0.6	2.6	800	0.4	0.2	0.7	2236
UGA	Uganda	DHS 2011	LI	ESA	62.1	58.0	66.0	0.4	0.1	1.4	778	0.5	0.3	1.0	2069
UGA	Uganda	DHS 2016	LI	ESA	65.5	62.3	68.6	0.5	0.2	1.2	1482	0.4	0.2	0.6	4160
UKR	Ukraine	MICS 2005	LMI	ECA	6.0	3.3	10.6	34.0	27.0	41.8	229	34.3	28.7	40.4	907
UKR	Ukraine	DHS 2007	LMI	ECA	18.2	11.4	27.8	28.0	19.0	39.2	98	42.3	36.0	48.8	338
UKR	Ukraine	MICS 2012	LMI	ECA	19.7	13.8	27.3	30.5	23.0	39.2	307	38.8	33.6	44.2	1256
UZB	Uzbekistan	DHS 1996	LMI	ECA	2.4	0.9	6.4	14.0	8.9	21.3	164	30.9	25.5	36.7	650
UZB	Uzbekistan	MICS 2006	LI	ECA	26.7	21.9	32.1	11.5	8.5	15.4	446	21.5	18.9	24.3	1663
VNM	Vietnam	DHS 1997	LI	EAP	15.7	11.0	22.0	3.9	1.9	7.6	237	7.9	5.9	10.6	873
VNM	Vietnam	DHS 2002	LI	EAP	15.5	10.1	23.0	2.2	0.7	6.2	191	6.6	4.8	9.2	642
VNM	Vietnam	MICS 2006	LI	EAP	16.9	11.6	24.0	34.1	26.6	42.5	229	34.7	29.8	39.8	808
VNM	Vietnam	MICS 2010	LMI	EAP	17.0	12.5	22.8	42.1	35.6	49.0	319	27.4	24.3	30.8	1110
VNM	Vietnam	MICS 2013	LMI	EAP	24.3	19.4	30.1	37.5	32.0	43.3	358	41.8	38.1	45.6	1118
ZMB	Zambia	DHS 1996	LI	ESA	18.8	15.9	22.1	3.1	2.0	4.9	676	1.8	1.2	2.7	1923
ZMB	Zambia	DHS 2001	LI	ESA	40.1	35.8	44.6	2.7	1.5	4.7	650	4.0	3.1	5.1	1923

ISO	Country	Survey and year	Income group*	Region of the world**	Exclusive breastfeeding under six months			Formula consumption under six months			Number of children	Formula consumption between 6-23 months			Number of children
					Prevalence	95% CI		Prevalence	95% CI			Prevalence	95% CI		
ZMB	Zambia	DHS 2007	LI	ESA	60.9	56.8	64.8	2.5	1.4	4.3	618	2.6	1.8	3.5	1829
ZMB	Zambia	DHS 2013	LMI	ESA	71.9	68.8	74.8	1.8	1.1	2.9	1189	1.5	1.1	2.2	3722
ZMB	Zambia	DHS 2018	LMI	ESA	69.9	66.6	72.9	2.3	1.4	3.6	1019	1.7	1.2	2.4	2785
ZWE	Zimbabwe	DHS 1994	LI	ESA	11.3	8.2	15.5	5.0	2.9	8.4	400	4.5	3.3	6.1	1063
ZWE	Zimbabwe	DHS 1999	LI	ESA	31.9	26.1	38.3	3.2	1.5	6.5	347	1.6	1.0	2.6	1000
ZWE	Zimbabwe	DHS 2005	LI	ESA	22.2	18.6	26.2	40.1	35.5	45.0	534	62.5	59.7	65.3	1445
ZWE	Zimbabwe	MICS 2009	LI	ESA	25.9	22.1	30.2	3.2	2.0	5.2	681	2.4	1.8	3.3	2147
ZWE	Zimbabwe	DHS 2010	LI	ESA	31.4	27.5	35.7	2.2	1.3	3.6	641	2.2	1.6	3.1	1649
ZWE	Zimbabwe	MICS 2014	LI	ESA	41.0	37.2	45.0	3.4	2.3	5.0	867	2.8	2.2	3.5	2965
ZWE	Zimbabwe	DHS 2015	LI	ESA	47.7	43.2	52.2	2.8	1.6	4.6	603	1.6	1.1	2.3	1628
ZWE	Zimbabwe	MICS 2019	LMI	ESA	41.9	37.5	46.4	3.8	2.4	5.9	605	2.6	1.9	3.5	1737

*LI: low income, LMI: lower-middle income, UMI: Upper-middle income.
**EAP: East Asia and Pacific; ESA: Eastern and Southern Africa; ECA: Eastern Europe and Central Asia; LAC: Latin America and Caribbean; MENA: Middle East and North Africa; SA: South Asia; WCA: West and Central Africa.
***Data on formula consumption not available in the survey.

Supplementary table 3. Countries, surveys, prevalence, and number of children included in the trend analysis of animal milk consumption indicators in low- and-middle income countries. Source: Demographic Health Survey (DHS), Multiple Indicator Cluster Survey (MICS), Reproductive and Health Survey (RHS).

ISO	Country	Survey and year	Income group*	Region of the world**	Animal milk consumption under six months			Number of children	Animal milk consumption between 6-23 months			Number of children
					Prevalence	95% CI			Prevalence	95% CI		
AFG	Afghanistan	MICS 2010	LI	SA	15.5	13.1	18.3	1270	29.6	27.1	32.3	3635
AFG	Afghanistan	DHS 2015	LI	SA	32.5	29.1	36.1	3203	37.2	34.9	39.6	8078
ALB	Albania	MICS 2005	LMI	ECA	21.6	14.2	31.5	99	52.4	44.7	59.9	273
ALB	Albania	DHS 2008	LMI	ECA	17.2	10.5	26.7	138	58.5	51.8	65.0	386
ALB	Albania	DHS 2017	UMI	ECA	19.0	12.6	27.4	285	41.6	35.7	47.6	766
ARM	Armenia	DHS 2000	LI	ECA	8.0	4.7	13.2	155	18.2	14.6	22.6	435
ARM	Armenia	DHS 2005	LMI	ECA	22.7	14.0	34.7	157	44.3	37.9	50.9	391
ARM	Armenia	DHS 2010	LMI	ECA	17.0	9.3	29.1	156	43.3	37.9	48.8	464
ARM	Armenia	DHS 2015	LMI	ECA	16.1	10.9	23.1	177	34.4	30.4	38.7	499
BGD	Bangladesh	DHS 1993	LI	SA	17.8	15.1	20.9	616	27.4	25.3	29.7	1688
BGD	Bangladesh	DHS 1996	LI	SA	18.4	15.2	22.2	612	27.2	24.4	30.1	1593
BGD	Bangladesh	DHS 1999	LI	SA	12.3	10.0	15.0	746	27.2	24.7	29.8	1786
BGD	Bangladesh	DHS 2004	LI	SA	14.4	11.3	18.1	679	33.7	30.8	36.7	1791
BGD	Bangladesh	MICS 2006	LI	SA	25.9	23.7	28.3	2300	40.1	38.6	41.7	9453
BGD	Bangladesh	DHS 2007	LI	SA	18.3	14.5	22.8	510	33.7	31.1	36.3	1718
BGD	Bangladesh	DHS 2011	LI	SA	8.5	6.0	11.9	789	24.8	22.7	27.0	2356
BGD	Bangladesh	MICS 2012	LI	SA	10.7	9.1	12.5	1959	27.4	25.5	29.4	5968
BGD	Bangladesh	DHS 2014	LMI	SA	8.4	5.8	12.1	632	29.8	27.2	32.5	2336
BGD	Bangladesh	MICS 2019	LMI	SA	6.4	5.4	7.7	2370	21.3	20.1	22.5	6691
BLR	Belarus	MICS 2005	LMI	ECA	17.1	10.5	26.6	183	72.5	69.2	75.5	986
BLR	Belarus	MICS 2012	UMI	ECA	3.8	1.6	8.5	247	47.4	43.4	51.6	1115
BLZ	Belize	RHS 1991	LMI	LAC	26.8	18.9	36.6	232	26.2	19.4	34.4	612
BLZ	Belize	MICS 2006	UMI	LAC	39.4	29.0	50.9	88	63.4	56.4	69.9	239
BLZ	Belize	MICS 2011	LMI	LAC	10.3	6.0	17.2	145	43.2	38.6	47.9	610
BLZ	Belize	MICS 2015	UMI	LAC	11.1	6.8	17.6	165	45.1	40.0	50.3	730

ISO	Country	Survey and year	Income group*	Region of the world**	Animal milk consumption under six months			Number of children	Animal milk consumption between 6-23 months			Number of children
					Prevalence	95% CI			Prevalence	95% CI		
BEN	Benin	DHS 1996	LI	WCA	4.7	3.1	7.2	518	15.2	11.9	19.2	1370
BEN	Benin	DHS 2001	LI	WCA	1.3	0.6	2.8	527	6.8	5.3	8.7	1436
BEN	Benin	DHS 2006	LI	WCA	3.9	3.0	5.0	1525	10.9	9.6	12.4	4593
BEN	Benin	DHS 2011	LI	WCA	7.0	5.5	9.0	1195	9.1	7.9	10.4	3739
BEN	Benin	MICS 2014	LI	WCA	4.0	2.8	5.6	1284	13.7	11.8	15.8	3639
BEN	Benin	DHS 2017	LI	WCA	3.0	2.1	4.3	1381	13.8	12.1	15.7	3884
BOL	Bolivia	DHS 1994	LMI	LAC	24.7	20.7	29.3	556	51.2	48.1	54.2	1622
BOL	Bolivia	DHS 1998	LMI	LAC	21.6	18.2	25.4	629	53.7	51.0	56.2	1984
BOL	Bolivia	DHS 2003	LMI	LAC	1.2	0.7	2.2	867	11.8	10.2	13.5	2703
BOL	Bolivia	DHS 2008	LMI	LAC	5.8	4.2	8.0	791	31.1	28.8	33.6	2461
BIH	Bosnia and Herzegovina	MICS 2006	LMI	ECA	40.3	30.0	51.5	186	84.6	81.2	87.5	1008
BIH	Bosnia and Herzegovina	MICS 2011	UMI	ECA	17.8	9.6	30.4	117	73.9	67.8	79.2	635
BFA	Burkina Faso	DHS 1998	LI	WCA	1.9	1.0	3.6	618	4.3	3.2	5.8	1491
BFA	Burkina Faso	DHS 2003	LI	WCA	0.4	0.1	1.3	1071	5.7	4.5	7.0	2782
BFA	Burkina Faso	MICS 2006	LI	WCA	16.9	11.8	23.6	543	28.0	20.9	36.4	1708
BFA	Burkina Faso	DHS 2010	LI	WCA	1.3	0.8	2.2	1454	9.2	7.9	10.6	4148
BDI	Burundi	DHS 2010	LI	ESA	0.8	0.5	1.5	685	5.1	4.1	6.3	2196
BDI	Burundi	DHS 2016	LI	ESA	1.4	0.8	2.5	1236	5.4	4.7	6.3	3858
CAF	Central Afr. Republic	DHS 1994	LI	WCA	1.8	0.8	3.9	458	7.0	5.7	8.6	1215
CAF	Central Afr. Republic	MICS 2006	LI	WCA	12.4	9.7	15.7	1266	16.0	13.7	18.6	2759
CAF	Central Afr. Republic	MICS 2010	LI	WCA	1.9	1.1	3.4	1283	6.8	5.6	8.2	3266
KHM	Cambodia	DHS 2000	LI	EAP	0.4	0.1	1.2	873	1.2	0.8	1.7	2099
KHM	Cambodia	DHS 2005	LI	EAP	2.7	1.6	4.5	788	5.1	4.0	6.3	2329
KHM	Cambodia	DHS 2010	LI	EAP	4.0	2.4	6.8	717	10.4	8.9	12.2	2368
KHM	Cambodia	DHS 2014	LI	EAP	3.7	2.3	5.7	688	22.0	19.7	24.5	2127
CMR	Cameroon	DHS 1998	LI	WCA	8.9	6.2	12.7	389	11.2	9.1	13.6	1009
CMR	Cameroon	DHS 2004	LI	WCA	3.2	2.1	4.8	794	7.5	6.3	8.8	2150
CMR	Cameroon	MICS 2006	LMI	WCA	16.3	12.8	20.4	658	10.9	9.3	12.8	2053

ISO	Country	Survey and year	Income group*	Region of the world**	Animal milk consumption under six months			Number of children	Animal milk consumption between 6-23 months			Number of children
					Prevalence	95% CI			Prevalence	95% CI		
CMR	Cameroon	DHS 2011	LMI	WCA	15.0	12.6	17.8	1123	21.6	19.6	23.8	3287
CMR	Cameroon	MICS 2014	LMI	WCA	5.3	3.5	7.8	703	20.8	18.0	23.9	2128
CMR	Cameroon	DHS 2018	LMI	WCA	4.0	2.7	5.8	986	10.9	9.4	12.6	2576
TCD	Chad	DHS 1996	LI	WCA	6.2	4.5	8.5	807	22.0	19.4	24.7	1813
TCD	Chad	DHS 2004	LI	WCA	17.0	13.6	21.1	583	30.9	27.3	34.8	1371
TCD	Chad	MICS 2010	LI	WCA	8.0	6.4	10.0	1867	19.2	17.5	21.0	4579
TCD	Chad	DHS 2014	LI	WCA	12.2	10.3	14.6	1823	21.0	19.1	23.0	4403
COL	Colombia	DHS 1995	LMI	LAC	30.0	25.5	34.9	440	69.8	67.0	72.5	1454
COL	Colombia	DHS 2000	LMI	LAC	2.6	1.5	4.3	430	9.3	7.9	11.0	1335
COL	Colombia	DHS 2005	LMI	LAC	1.7	1.0	3.0	1397	9.9	8.7	11.2	4110
COL	Colombia	DHS 2010	UMI	LAC	6.4	5.0	8.1	1515	45.0	43.2	46.9	5023
COM	Comoros	DHS 1996	LI	ESA	25.8	19.0	34.0	186	29.9	25.7	34.5	522
COM	Comoros	DHS 2012	LI	ESA	15.2	11.1	20.3	327	17.3	14.1	21.1	869
COD	Congo Dem. Republic	DHS 2007	LI	WCA	5.6	3.8	8.0	907	11.9	9.8	14.3	2355
COD	Congo Dem. Republic	MICS 2010	LI	WCA	3.5	2.4	5.1	1271	10.7	9.0	12.7	3617
COD	Congo Dem. Republic	DHS 2013	LI	WCA	2.7	1.7	4.5	1934	6.7	5.6	8.1	4991
COD	Congo Dem. Republic	MICS 2017	LI	WCA	2.1	1.2	3.5	2106	6.9	5.5	8.6	6499
CIV	Cote d'Ivoire	DHS 1994	LI	WCA	4.2	2.8	6.4	658	4.9	3.8	6.2	1710
CIV	Cote d'Ivoire	DHS 1998	LI	WCA	11.5	7.2	17.8	166	13.8	10.4	18.2	583
CIV	Cote d'Ivoire	MICS 2006	LI	WCA	11.5	8.6	15.3	950	18.8	15.7	22.3	2765
CIV	Cote d'Ivoire	DHS 2011	LMI	WCA	3.2	1.9	5.3	775	7.4	5.9	9.2	2145
CIV	Cote d'Ivoire	MICS 2016	LMI	WCA	6.0	3.7	9.5	981	15.8	13.8	17.9	2668
CUB	Cuba	MICS 2006	LMI	LAC	56.9	52.3	61.4	774	89.5	87.5	91.1	2712
CUB	Cuba	MICS 2010	UMI	LAC	32.1	23.4	42.2	1002	82.5	78.8	85.7	3600
CUB	Cuba	MICS 2014	UMI	LAC	50.9	37.9	63.7	381	80.7	75.7	84.8	1775
DOM	Dominican Republic	DHS 1996	LMI	LAC	15.1	11.5	19.6	415	77.9	74.6	80.8	1205
DOM	Dominican Republic	DHS 1999	LMI	LAC	7.6			52	74.6			140
DOM	Dominican Republic	DHS 2002	LMI	LAC	2.9	1.7	5.1	1003	3.4	2.6	4.3	3098

ISO	Country	Survey and year	Income group*	Region of the world**	Animal milk consumption under six months			Number of children	Animal milk consumption between 6-23 months			Number of children
					Prevalence	95% CI			Prevalence	95% CI		
DOM	Dominican Republic	DHS 2007	LMI	LAC	74.8	70.0	79.0	986	84.9	83.2	86.6	2915
DOM	Dominican Republic	DHS 2013	UMI	LAC	51.7	44.3	59.1	307	75.7	72.2	78.9	1048
DOM	Dominican Republic	MICS 2014	UMI	LAC	60.1	56.0	64.0	1656	79.5	77.9	81.1	6235
EGY	Egypt	DHS 1995	LMI	MENA	16.1	13.7	18.8	1138	46.7	44.6	48.9	3182
EGY	Egypt	DHS 2000	LMI	MENA	12.4	10.6	14.5	1201	51.0	48.8	53.1	3127
EGY	Egypt	DHS 2005	LMI	MENA	13.4	10.1	17.6	1250	54.7	51.5	58.0	3891
EGY	Egypt	DHS 2008	LMI	MENA	10.7	8.9	12.8	1130	49.4	47.4	51.4	3324
EGY	Egypt	DHS 2014	LMI	MENA	6.2	4.8	8.0	1487	24.0	22.4	25.7	4834
SWZ	Eswatini	DHS 2006	LMI	ESA	28.9	23.4	35.0	258	33.2	29.5	37.1	767
SWZ	Eswatini	MICS 2010	LMI	ESA	3.2	1.3	7.4	269	19.9	16.9	23.2	773
SWZ	Eswatini	MICS 2014	LMI	ESA	1.5	0.5	4.7	235	24.0	20.2	28.2	789
ETH	Ethiopia	DHS 2000	LI	ESA	4.6	3.0	6.9	966	9.4	7.9	11.3	2714
ETH	Ethiopia	DHS 2005	LI	ESA	21.7	18.8	25.0	1008	39.9	36.4	43.5	2520
ETH	Ethiopia	DHS 2011	LI	ESA	13.7	10.7	17.3	1187	22.3	19.5	25.3	2850
ETH	Ethiopia	DHS 2016	LI	ESA	5.7	4.3	7.7	1092	16.9	14.6	19.6	2822
GAB	Gabon	DHS 2000	UMI	WCA	53.1	47.9	58.2	451	55.1	51.6	58.5	1183
GAB	Gabon	DHS 2012	UMI	WCA	10.8	7.3	15.6	631	37.9	33.6	42.4	1719
GMB	Gambia	MICS 2005	LI	WCA	12.0	9.7	14.7	855	30.9	28.4	33.4	2177
GMB	Gambia	MICS 2010	LI	WCA	4.7	3.3	6.7	1412	14.8	12.9	17.0	3790
GMB	Gambia	DHS 2013	LI	WCA	2.1	1.1	3.9	951	11.1	9.1	13.5	2424
GMB	Gambia	MICS 2018	LI	WCA	2.3	1.4	3.6	910	19.3	17.2	21.5	2724
GEO	Georgia	MICS 2005	LMI	ECA	30.7	23.5	39.0	171	55.2	50.3	60.1	577
GEO	Georgia	MICS 2018	UMI	ECA	2.1	0.7	5.9	234	20.6	17.0	24.8	701
GHA	Ghana	DHS 1993	LI	WCA	4.0	2.3	6.7	379	6.5	5.1	8.3	968
GHA	Ghana	DHS 1998	LI	WCA	5.1	3.1	8.2	302	11.8	9.7	14.3	950
GHA	Ghana	DHS 2003	LI	WCA	3.4	2.0	5.5	338	11.2	9.1	13.8	1102
GHA	Ghana	MICS 2006	LI	WCA	7.3	4.6	11.4	384	17.6	14.6	21.0	1043
GHA	Ghana	DHS 2008	LI	WCA	6.6	4.1	10.4	318	23.0	19.6	26.7	857

ISO	Country	Survey and year	Income group*	Region of the world**	Animal milk consumption under six months			Number of children	Animal milk consumption between 6-23 months			Number of children
					Prevalence	95% CI			Prevalence	95% CI		
GHA	Ghana	MICS 2011	LMI	WCA	6.7	3.9	11.1	792	24.8	21.7	28.2	2171
GHA	Ghana	DHS 2014	LMI	WCA	7.4	5.0	10.9	606	14.7	12.3	17.5	1656
GHA	Ghana	MICS 2017	LMI	WCA	3.5	2.1	5.6	891	11.2	9.1	13.6	2585
GTM	Guatemala	DHS 1995	LMI	LAC	13.3	10.5	16.9	969	29.2	26.1	32.6	2820
GTM	Guatemala	DHS 1998	LMI	LAC	11.8	7.9	17.3	476	33.7	28.2	39.7	1324
GTM	Guatemala	RHS 2002	LMI	LAC	9.4	6.9	12.8	683	29.2	26.5	32.2	2157
GTM	Guatemala	RHS 2008	LMI	LAC	4.0	2.7	5.8	1084	19.8	17.9	21.8	3003
GTM	Guatemala	DHS 2014	LMI	LAC	1.7	1.0	2.8	1175	19.2	17.5	21.0	3509
GIN	Guinea	DHS 1999	LI	WCA	13.3	10.5	16.6	662	21.1	18.4	24.2	1321
GIN	Guinea	DHS 2005	LI	WCA	1.9	1.1	3.2	733	7.3	5.9	9.1	1650
GIN	Guinea	DHS 2012	LI	WCA	2.7	1.7	4.3	718	11.1	9.4	12.9	1953
GIN	Guinea	MICS 2016	LI	WCA	7.0	5.0	9.8	686	14.3	12.4	16.4	2122
GIN	Guinea	DHS 2018	LI	WCA	4.1	2.9	5.7	916	14.9	12.9	17.2	1909
GNB	Guinea Bissau	MICS 2006	LI	WCA	15.3	12.5	18.8	658	17.7	15.2	20.6	1837
GNB	Guinea Bissau	MICS 2014	LI	WCA	7.5	5.5	10.3	830	12.8	11.0	14.8	2268
GUY	Guyana	MICS 2006	LMI	LAC	57.6	50.4	64.5	219	84.7	80.9	87.8	716
GUY	Guyana	DHS 2009	LMI	LAC	40.5	31.2	50.7	230	76.0	71.6	80.0	607
GUY	Guyana	MICS 2014	LMI	LAC	44.4	37.1	51.9	290	73.4	69.5	77.0	1034
HTI	Haiti	DHS 1994	LI	LAC	21.4	17.5	25.8	317	20.8	17.4	24.5	931
HTI	Haiti	DHS 2000	LI	LAC	3.9	2.4	6.4	563	13.6	10.5	17.5	1797
HTI	Haiti	DHS 2005	LI	LAC	20.4	16.4	25.2	598	33.3	30.2	36.6	1691
HTI	Haiti	DHS 2012	LI	LAC	4.1	2.6	6.3	726	13.8	11.8	16.0	1972
HTI	Haiti	DHS 2016	LI	LAC	17.3	14.3	20.9	700	30.7	27.9	33.6	1652
HND	Honduras	RHS 1991	LMI	LAC	91.1	88.5	93.1	571	90.4	88.8	91.8	1633
HND	Honduras	RHS 1996	LI	LAC	37.0	31.0	43.5	348	49.6	45.9	53.3	1168
HND	Honduras	RHS 2001	LMI	LAC	40.3	35.2	45.6	434	54.0	50.7	57.2	1617
HND	Honduras	DHS 2005	LMI	LAC	26.3	22.9	30.1	973	51.0	48.8	53.2	3066
HND	Honduras	DHS 2011	LMI	LAC	19.0	16.1	22.2	1084	51.4	49.2	53.5	3237

ISO	Country	Survey and year	Income group*	Region of the world**	Animal milk consumption under six months			Number of children	Animal milk consumption between 6-23 months			Number of children
					Prevalence	95% CI			Prevalence	95% CI		
IND	India	DHS 1998	LI	SA	22.6	21.2	24.2	5503	52.3	51.2	53.4	14747
IND	India	DHS 2005	LI	SA	19.1	17.6	20.6	4616	48.0	46.6	49.3	13904
IND	India	DHS 2015	LMI	SA	15.5	14.9	16.3	22626	44.6	44.0	45.2	71762
IDN	Indonesia	DHS 1994	LMI	EAP	13.9	11.5	16.6	1639	22.3	20.3	24.5	4799
IDN	Indonesia	DHS 1997	LMI	EAP	14.2	11.7	17.1	1678	24.2	22.3	26.3	4903
IDN	Indonesia	DHS 2002	LI	EAP	0.3	0.1	1.6	1641	3.2	2.1	4.9	4413
IDN	Indonesia	DHS 2007	LMI	EAP	1.7	1.2	2.6	1802	11.1	9.8	12.6	5209
IDN	Indonesia	DHS 2012	LMI	EAP	1.9	1.2	3.1	1686	12.6	11.2	14.1	5193
IDN	Indonesia	DHS 2017	LMI	EAP	0.8	0.4	1.4	1666	12.1	10.9	13.3	5033
IRQ	Iraq	MICS 2006	LMI	MENA	5.6	4.3	7.1	1624	32.5	30.7	34.3	5352
IRQ	Iraq	MICS 2011	LMI	MENA	6.6	5.3	8.2	3882	29.3	27.9	30.8	11168
IRQ	Iraq	MICS 2018	UMI	MENA	3.0	2.0	4.3	1681	20.5	17.4	24.0	4786
JAM	Jamaica	MICS 2005	LMI	LAC	15.6	9.9	23.8	129	47.2	42.1	52.4	412
JAM	Jamaica	MICS 2011	UMI	LAC	6.4	3.5	11.4	167	35.2	29.9	40.8	476
JOR	Jordan	DHS 1997	LMI	MENA	9.0	6.7	11.9	499	57.9	55.3	60.5	1767
JOR	Jordan	DHS 2002	LMI	MENA	1.5	0.7	3.0	507	15.0	13.3	16.9	1728
JOR	Jordan	DHS 2007	LMI	MENA	28.9	24.4	33.8	1117	62.1	58.8	65.3	2739
JOR	Jordan	DHS 2012	UMI	MENA	55.7	50.1	61.2	850	69.2	66.2	72.1	2783
JOR	Jordan	DHS 2017	UMI	MENA	8.9	6.9	11.5	1218	43.3	40.2	46.4	2680
KAZ	Kazakhstan	DHS 1995	LMI	ECA	37.4	27.4	48.7	118	81.4	75.8	85.9	403
KAZ	Kazakhstan	DHS 1999	LMI	ECA	17.4	10.1	28.1	99	65.4	58.0	72.1	349
KAZ	Kazakhstan	MICS 2006	UMI	ECA	27.9	23.3	33.0	387	86.4	84.1	88.4	1437
KAZ	Kazakhstan	MICS 2010	UMI	ECA	8.9	6.7	11.8	543	61.0	58.2	63.8	1582
KAZ	Kazakhstan	MICS 2015	UMI	ECA	4.2	2.5	6.9	508	51.0	47.7	54.4	1632
KEN	Kenya	DHS 1993	LI	ESA	44.5	38.7	50.4	518	55.5	52.4	58.6	1645
KEN	Kenya	DHS 1998	LI	ESA	44.7	39.9	49.7	518	73.3	70.7	75.7	1624
KEN	Kenya	DHS 2003	LI	ESA	7.2	5.1	10.1	599	15.5	13.3	17.9	1610
KEN	Kenya	DHS 2008	LI	ESA	33.2	28.4	38.4	587	57.9	54.0	61.7	1656

ISO	Country	Survey and year	Income group*	Region of the world**	Animal milk consumption under six months			Number of children	Animal milk consumption between 6-23 months			Number of children
					Prevalence	95% CI			Prevalence	95% CI		
KEN	Kenya	DHS 2014	LMI	ESA	7.6	6.1	9.3	1810	24.1	22.7	25.6	5792
KGZ	Kyrgyzstan	DHS 1997	LI	ECA	24.6	17.3	33.8	177	66.6	60.2	72.4	521
KGZ	Kyrgyzstan	MICS 2005	LI	ECA	21.5	15.6	28.8	286	74.3	68.5	79.3	874
KGZ	Kyrgyzstan	DHS 2012	LI	ECA	3.6	2.0	6.6	447	27.6	24.5	30.9	1317
KGZ	Kyrgyzstan	MICS 2014	LMI	ECA	9.5	6.6	13.5	432	39.5	36.6	42.4	1402
KGZ	Kyrgyzstan	MICS 2018	LMI	ECA	2.5	1.3	4.6	390	38.8	34.4	43.3	992
LAO	Lao	MICS 2006	LI	EAP	8.9	6.3	12.5	445	17.2	14.3	20.4	1201
LAO	Lao	MICS 2011	LMI	EAP	2.7	1.8	4.2	1168	11.8	10.6	13.3	3265
LAO	Lao	MICS 2017	LMI	EAP	1.6	0.9	2.7	1134	22.1	20.3	24.0	3428
LSO	Lesotho	DHS 2004	LI	ESA	5.2	3.2	8.4	399	13.1	11.0	15.5	982
LSO	Lesotho	DHS 2009	LMI	ESA	11.4	8.3	15.5	434	19.8	17.0	22.9	1118
LSO	Lesotho	DHS 2014	LMI	ESA	4.4	2.0	9.4	327	22.1	19.0	25.5	948
LSO	Lesotho	MICS 2018	LMI	ESA	1.7	0.3	8.9	215	22.7	19.6	26.1	1009
LBR	Liberia	DHS 2007	LI	WCA	7.1	4.6	10.8	504	10.3	8.4	12.5	1519
LBR	Liberia	DHS 2013	LI	WCA	4.7	2.8	7.8	718	8.1	5.5	11.9	2155
MDG	Madagascar	DHS 1997	LI	ESA	4.1	2.7	6.1	628	11.8	9.8	14.2	1638
MDG	Madagascar	DHS 2003	LI	ESA	1.2	0.5	2.9	499	9.9	7.7	12.6	1538
MDG	Madagascar	DHS 2008	LI	ESA	9.5	7.7	11.7	1214	22.6	20.5	24.9	3306
MDG	Madagascar	MICS 2018	LI	ESA	2.6	1.9	3.7	1386	9.0	7.5	10.8	3859
MWI	Malawi	DHS 2000	LI	ESA	0.6	0.3	1.1	1242	3.8	3.0	4.7	3312
MWI	Malawi	DHS 2004	LI	ESA	1.4	0.8	2.4	1099	3.2	2.6	4.0	3327
MWI	Malawi	MICS 2006	LI	ESA	0.9	0.5	1.6	2298	4.5	3.9	5.2	7668
MWI	Malawi	DHS 2010	LI	ESA	3.5	2.4	5.1	1663	6.7	5.9	7.7	5688
MWI	Malawi	MICS 2013	LI	ESA	1.6	1.0	2.6	1686	7.8	6.7	9.0	5661
MWI	Malawi	DHS 2015	LI	ESA	1.0	0.6	1.7	1636	5.4	4.6	6.4	4747
MDV	Maldives	DHS 2009	LMI	SA	29.1	23.8	35.0	414	67.2	63.6	70.6	1256
MDV	Maldives	DHS 2016	UMI	SA	2.0	1.0	3.9	288	48.8	44.0	53.6	848
MLI	Mali	DHS 1995	LI	WCA	5.6	4.2	7.5	1036	20.0	18.1	22.1	2475

ISO	Country	Survey and year	Income group*	Region of the world**	Animal milk consumption under six months			Number of children	Animal milk consumption between 6-23 months			Number of children
					Prevalence	95% CI			Prevalence	95% CI		
MLI	Mali	DHS 2001	LI	WCA	2.1	1.5	3.1	1418	8.3	7.1	9.6	3372
MLI	Mali	DHS 2006	LI	WCA	3.6	2.6	4.8	1421	17.4	15.6	19.4	3780
MLI	Mali	MICS 2009	LI	WCA	7.0	5.8	8.4	2913	22.7	21.1	24.5	7816
MLI	Mali	DHS 2012	LI	WCA	12.0	9.9	14.5	999	28.4	26.0	30.8	2795
MLI	Mali	MICS 2015	LI	WCA	8.6	7.2	10.3	1663	30.5	28.4	32.6	4865
MLI	Mali	DHS 2018	LI	WCA	4.4	3.1	6.3	997	21.9	19.8	24.1	2713
MRT	Mauritania	MICS 2007	LI	WCA	38.5	34.8	42.3	915	65.4	62.8	67.8	2693
MRT	Mauritania	MICS 2011	LI	WCA	24.2	20.7	27.9	995	53.8	51.2	56.4	2749
MRT	Mauritania	MICS 2015	LMI	WCA	23.5	20.2	27.2	915	61.7	59.0	64.3	3184
MDA	Moldova	DHS 2005	LMI	ECA	16.2	10.4	24.2	158	62.9	58.1	67.5	486
MDA	Moldova	MICS 2012	LMI	ECA	12.8	7.6	20.9	176	45.6	41.0	50.3	591
MNG	Mongolia	MICS 2005	LI	EAP	23.0	18.9	27.8	399	72.2	68.9	75.3	1098
MNG	Mongolia	MICS 2010	LMI	EAP	10.2	7.3	14.1	410	37.6	34.7	40.6	1323
MNG	Mongolia	MICS 2013	LMI	EAP	10.7	8.5	13.3	644	48.3	45.8	50.8	1802
MNG	Mongolia	MICS 2018	LMI	EAP	8.4	5.6	12.3	616	45.5	42.0	49.0	1674
MNE	Montenegro	MICS 2005	UMI	ECA	27.4	17.3	40.6	71	86.2	81.4	89.9	284
MNE	Montenegro	MICS 2013	UMI	ECA	18.8	10.9	30.6	108	74.8	68.3	80.4	388
MNE	Montenegro	MICS 2018	UMI	ECA	4.4	1.8	10.1	101	52.6	41.4	63.5	332
MOZ	Mozambique	DHS 1997	LI	ESA	3.6	2.4	5.3	706	6.6	4.9	8.8	1893
MOZ	Mozambique	DHS 2003	LI	ESA	0.2	0.1	0.7	1027	2.2	1.7	2.9	2805
MOZ	Mozambique	MICS 2008	LI	ESA	0.7	0.4	1.5	1229	3.3	2.6	4.1	3652
MOZ	Mozambique	DHS 2011	LI	ESA	2.8	2.0	3.9	1044	4.2	3.4	5.1	3280
MOZ	Mozambique	DHS 2015	LI	ESA	10.8	7.8	14.7	514	15.0	12.2	18.3	1492
NAM	Namibia	DHS 2000	LMI	ESA	47.2	40.0	54.5	405	49.8	45.4	54.2	1091
NAM	Namibia	DHS 2006	LMI	ESA	25.1	20.6	30.3	495	37.6	34.4	40.8	1396
NAM	Namibia	DHS 2013	UMI	ESA	12.3	9.3	16.2	525	17.3	14.6	20.3	1303
NPL	Nepal	DHS 1996	LI	SA	14.6	12.0	17.6	677	42.5	39.0	46.2	2038
NPL	Nepal	DHS 2001	LI	SA	3.9	2.6	5.8	634	9.2	7.9	10.6	1876

ISO	Country	Survey and year	Income group*	Region of the world**	Animal milk consumption under six months			Number of children	Animal milk consumption between 6-23 months			Number of children
					Prevalence	95% CI			Prevalence	95% CI		
NPL	Nepal	DHS 2006	LI	SA	21.9	17.1	27.5	473	47.3	43.5	51.0	1542
NPL	Nepal	DHS 2011	LI	SA	13.6	9.9	18.4	497	43.9	40.1	47.8	1421
NPL	Nepal	MICS 2014	LI	SA	17.0	12.2	23.1	452	54.1	50.2	57.9	1556
NPL	Nepal	DHS 2016	LI	SA	12.6	9.4	16.7	467	48.3	44.8	51.9	1463
NER	Niger	DHS 1998	LI	WCA	6.5	4.8	8.7	816	12.8	11.0	14.8	2063
NER	Niger	DHS 2006	LI	WCA	5.0	3.7	6.7	945	13.6	11.7	15.7	2479
NER	Niger	DHS 2012	LI	WCA	3.7	2.5	5.5	1303	10.9	9.5	12.5	3260
NGA	Nigeria	DHS 1999	LI	WCA	9.9	7.5	12.9	555	11.6	10.0	13.4	1620
NGA	Nigeria	DHS 2003	LI	WCA	4.3	2.6	6.9	611	9.5	7.8	11.6	1586
NGA	Nigeria	MICS 2007	LI	WCA	15.0	12.8	17.4	1729	30.4	28.0	32.8	4788
NGA	Nigeria	DHS 2008	LMI	WCA	16.9	15.2	18.6	2889	30.9	29.1	32.7	7557
NGA	Nigeria	MICS 2011	LMI	WCA	9.0	7.6	10.5	2714	26.9	25.0	28.9	7528
NGA	Nigeria	DHS 2013	LMI	WCA	4.6	3.7	5.6	2934	12.4	11.3	13.6	8721
NGA	Nigeria	MICS 2016	LMI	WCA	8.5	7.3	9.8	2748	23.1	21.8	24.5	8219
NGA	Nigeria	DHS 2018	LMI	WCA	3.3	2.6	4.0	3193	14.9	13.7	16.2	8883
MKD	North Macedonia	MICS 2005	LMI	ECA	46.9	30.0	64.6	233	77.9	69.9	84.2	1199
MKD	North Macedonia	MICS 2011	UMI	ECA	14.0	8.1	22.9	112	63.8	57.7	69.4	410
PAK	Pakistan	DHS 2006	LI	SA	41.4	37.7	45.2	947	64.0	61.6	66.3	2194
PAK	Pakistan	DHS 2012	LMI	SA	30.2	26.2	34.4	1076	49.3	46.7	51.9	2870
PAK	Pakistan	DHS 2017	LMI	SA	24.2	21.0	27.7	1117	49.1	45.7	52.4	2566
PER	Peru	DHS 1996	LMI	LAC	9.4	7.7	11.4	1519	32.5	30.8	34.4	4729
PER	Peru	DHS 2000	LMI	LAC	2.8	1.7	4.4	1162	15.1	13.6	16.7	3699
PER	Peru	DHS 2004	LMI	LAC	9.7	6.3	14.7	233	68.0	63.3	72.3	722
PER	Peru	DHS 2005	LMI	LAC	10.1	5.8	16.9	273	56.7	52.1	61.2	801
PER	Peru	DHS 2006	LMI	LAC	7.7	4.4	13.1	251	63.0	58.2	67.6	842
PER	Peru	DHS 2007	LMI	LAC	8.5	4.5	15.6	223	56.4	51.3	61.4	752
PER	Peru	DHS 2008	UMI	LAC	9.5	6.4	13.8	545	59.7	55.8	63.4	1834
PER	Peru	DHS 2009	UMI	LAC	7.9	5.9	10.7	901	54.1	51.5	56.6	3027

ISO	Country	Survey and year	Income group[*]	Region of the world[**]	Animal milk consumption under six months			Number of children	Animal milk consumption between 6-23 months			Number of children
					Prevalence	95% CI			Prevalence	95% CI		
PER	Peru	DHS 2010	UMI	LAC	8.4	6.1	11.4	766	52.1	49.6	54.5	2706
PER	Peru	DHS 2011	UMI	LAC	6.3	4.5	8.8	780	54.9	52.3	57.4	2557
PER	Peru	DHS 2012	UMI	LAC	5.4	3.8	7.8	827	48.3	45.5	51.2	2729
PER	Peru	DHS 2013	UMI	LAC	5.3	2.8	9.8	750	50.9	48.4	53.5	2580
PER	Peru	DHS 2014	UMI	LAC	7.2	5.1	10.1	878	47.5	45.1	49.9	2767
PER	Peru	DHS 2015	UMI	LAC	5.6	4.4	7.2	1847	49.0	47.3	50.6	7040
PER	Peru	DHS 2016	UMI	LAC	4.7	3.5	6.3	1446	43.4	41.7	45.1	6198
PER	Peru	DHS 2017	UMI	LAC	5.0	3.7	6.6	1792	40.1	38.5	41.8	6580
PER	Peru	DHS 2018	UMI	LAC	5.4	4.1	7.0	1737	39.9	38.3	41.6	6534
PHL	Philippines	DHS 1993	LMI	EAP	10.5	8.5	13.0	821	13.6	12.2	15.1	2481
PHL	Philippines	DHS 1998	LMI	EAP	7.9	6.0	10.3	757	43.0	40.7	45.2	2137
PHL	Philippines	DHS 2003	LMI	EAP	1.0	0.4	2.3	624	5.1	4.2	6.1	1940
PHL	Philippines	DHS 2008	LMI	EAP	18.4	15.2	22.1	593	54.4	52.1	56.6	1818
RWA	Rwanda	DHS 2000	LI	ESA	1.6	0.9	2.8	757	3.7	2.9	4.7	2115
RWA	Rwanda	DHS 2005	LI	ESA	0.9	0.4	1.8	876	4.9	4.0	6.1	2340
RWA	Rwanda	DHS 2010	LI	ESA	3.5	2.4	4.9	709	18.4	16.6	20.4	2333
RWA	Rwanda	DHS 2014	LI	ESA	3.5	2.3	5.1	703	22.4	20.4	24.6	2354
STP	Sao Tome and Principe	DHS 2008	LMI	WCA	18.2	12.9	24.9	192	27.5	23.1	32.4	563
STP	Sao Tome and Principe	MICS 2014	LMI	WCA	2.1	0.5	7.7	169	22.6	18.6	27.3	571
SEN	Senegal	DHS 1997	LI	WCA	13.8	11.2	17.0	817	19.3	17.2	21.5	1929
SEN	Senegal	DHS 2005	LI	WCA	5.1	3.5	7.4	1290	25.2	22.7	27.8	3060
SEN	Senegal	DHS 2010	LMI	WCA	7.1	5.3	9.4	1334	31.7	29.3	34.2	3375
SEN	Senegal	DHS 2012	LMI	WCA	4.5	2.8	7.1	672	28.1	25.1	31.4	1935
SEN	Senegal	DHS 2014	LMI	WCA	4.3	2.6	7.0	612	25.4	21.5	29.8	1885
SEN	Senegal	DHS 2015	LI	WCA	3.2	1.8	5.7	626	29.7	26.5	33.1	1984
SEN	Senegal	DHS 2016	LI	WCA	2.9	1.4	6.0	605	26.3	22.7	30.2	1940
SEN	Senegal	DHS 2017	LI	WCA	2.9	1.8	4.5	1142	26.4	24.0	28.9	3489
SRB	Serbia	MICS 2005	UMI	ECA	22.8	17.6	29.0	334	78.7	75.5	81.5	1129

ISO	Country	Survey and year	Income group*	Region of the world**	Animal milk consumption under six months			Number of children	Animal milk consumption between 6-23 months			Number of children
					Prevalence	95% CI			Prevalence	95% CI		
SRB	Serbia	MICS 2010	UMI	ECA	10.3	6.5	16.0	246	76.0	71.9	79.6	1011
SRB	Serbia	MICS 2014	UMI	ECA	5.7	3.0	10.8	169	64.1	57.4	70.3	795
SLE	Sierra Leone	MICS 2005	LI	WCA	8.3	6.0	11.2	508	10.9	9.0	13.0	1592
SLE	Sierra Leone	DHS 2008	LI	WCA	9.2	7.0	12.0	617	13.3	11.4	15.4	1537
SLE	Sierra Leone	MICS 2010	LI	WCA	3.5	2.2	5.4	831	7.3	6.0	8.8	2442
SLE	Sierra Leone	DHS 2013	LI	WCA	4.4	2.8	6.7	1115	8.4	6.9	10.3	3088
SLE	Sierra Leone	MICS 2017	LI	WCA	5.3	3.9	7.3	1170	12.5	11.1	14.1	3411
ZAF	South Africa	DHS 1998	LMI	ESA	28.5	23.7	33.8	505	41.6	38.3	45.0	1374
ZAF	South Africa	DHS 2016	UMI	ESA	10.6	7.0	15.7	346	31.4	27.7	35.5	877
SUR	Suriname	MICS 2006	LMI	LAC	48.7	40.3	57.2	182	61.6	56.7	66.2	659
SUR	Suriname	MICS 2010	UMI	LAC	41.3	34.4	48.6	304	51.3	47.5	55.1	1062
SUR	Suriname	MICS 2018	UMI	LAC	14.3	9.6	20.7	335	18.7	15.8	22.0	1182
TJK	Tajikistan	MICS 2005	LI	ECA	20.4	16.0	25.8	388	53.7	49.7	57.6	1275
TJK	Tajikistan	DHS 2012	LI	ECA	9.1	6.4	12.6	424	39.9	36.7	43.3	1467
TJK	Tajikistan	DHS 2017	LI	ECA	10.5	7.8	14.0	553	40.5	37.7	43.4	1722
TZA	Tanzania	DHS 1996	LI	ESA	17.0	14.3	20.2	654	34.1	31.4	37.0	1920
TZA	Tanzania	DHS 2004	LI	ESA	2.8	1.8	4.2	825	9.9	8.4	11.6	2408
TZA	Tanzania	DHS 2010	LI	ESA	13.5	10.2	17.7	803	33.1	30.1	36.4	2235
TZA	Tanzania	DHS 2015	LI	ESA	6.0	4.5	8.0	1015	17.7	16.0	19.5	3020
THA	Thailand	MICS 2005	LMI	EAP	53.4	48.1	58.6	873	82.8	80.2	85.2	2913
THA	Thailand	MICS 2012	UMI	EAP	8.5	5.2	13.5	591	37.0	33.5	40.7	2618
THA	Thailand	MICS 2015	UMI	EAP	12.6	8.0	19.4	661	58.3	53.9	62.5	3222
TLS	Timor-Leste	DHS 2009	LMI	EAP	15.7	12.6	19.4	960	17.4	15.6	19.5	2570
TLS	Timor-Leste	DHS 2016	LMI	EAP	8.0	5.6	11.3	743	11.5	9.7	13.6	1950
TGO	Togo	DHS 1998	LI	WCA	1.6	0.8	3.2	692	3.8	2.9	5.0	1878
TGO	Togo	MICS 2006	LI	WCA	14.0	10.5	18.6	447	12.4	10.2	15.0	1341
TGO	Togo	MICS 2010	LI	WCA	2.0	1.0	4.2	563	6.3	4.9	8.2	1392
TGO	Togo	DHS 2013	LI	WCA	0.9	0.4	2.0	603	5.0	3.9	6.3	2070

ISO	Country	Survey and year	Income group*	Region of the world**	Animal milk consumption under six months			Number of children	Animal milk consumption between 6-23 months			Number of children
					Prevalence	95% CI			Prevalence	95% CI		
TGO	Togo	MICS 2017	LI	WCA	1.6	0.5	5.4	504	7.3	5.9	9.1	1461
TUN	Tunisia	MICS 2011	UMI	MENA	51.1	44.1	58.0	306	73.6	69.9	77.1	854
TUN	Tunisia	MICS 2018	LMI	MENA	5.6	3.4	9.2	299	65.3	61.8	68.5	946
TUR	Turkey	DHS 1993	LMI	ECA	20.9	16.8	25.8	362	18.9	16.6	21.4	1034
TUR	Turkey	DHS 1998	UMI	ECA	24.5	19.5	30.4	370	62.5	58.9	66.1	1012
TUR	Turkey	DHS 2003	LMI	ECA	16.4	12.6	21.1	413	57.9	54.0	61.7	1156
TUR	Turkey	DHS 2013	UMI	ECA	11.4	8.3	15.6	332	48.7	45.3	52.0	1053
TKM	Turkmenistan	MICS 2006	LMI	ECA	28.7	21.5	37.2	237	72.9	67.9	77.3	625
TKM	Turkmenistan	MICS 2015	UMI	ECA	1.6	0.6	4.3	342	47.3	44.1	50.6	1169
UGA	Uganda	DHS 1995	LI	ESA	21.6	17.7	26.0	629	32.6	29.6	35.8	2079
UGA	Uganda	DHS 2000	LI	ESA	4.8	3.3	7.0	656	8.7	7.3	10.2	1994
UGA	Uganda	DHS 2006	LI	ESA	17.4	14.5	20.7	800	29.9	27.0	32.9	2236
UGA	Uganda	DHS 2011	LI	ESA	14.0	11.2	17.3	778	28.5	25.8	31.4	2069
UGA	Uganda	DHS 2016	LI	ESA	11.0	9.1	13.3	1482	28.0	26.0	30.0	4160
UKR	Ukraine	MICS 2005	LMI	ECA	32.2	25.0	40.2	229	77.3	72.4	81.5	907
UKR	Ukraine	DHS 2007	LMI	ECA	40.3	29.4	52.1	98	76.9	71.1	81.9	338
UKR	Ukraine	MICS 2012	LMI	ECA	11.9	6.6	20.6	307	68.4	63.8	72.6	1256
UZB	Uzbekistan	DHS 1996	LMI	ECA	35.1	26.7	44.5	164	75.9	69.9	81.1	650
UZB	Uzbekistan	MICS 2006	LI	ECA	13.7	10.6	17.5	446	57.4	53.7	61.0	1663
VNM	Vietnam	DHS 1997	LI	EAP	25.6	19.9	32.3	237	26.1	23.0	29.5	873
VNM	Vietnam	DHS 2002	LI	EAP	35.5	27.7	44.1	191	44.1	38.7	49.7	642
VNM	Vietnam	MICS 2006	LI	EAP	18.0	12.2	25.6	229	54.6	49.0	60.2	808
VNM	Vietnam	MICS 2010	LMI	EAP	7.5	4.6	11.9	319	55.8	52.3	59.3	1110
VNM	Vietnam	MICS 2013	LMI	EAP	16.2	12.1	21.4	358	56.1	52.3	59.9	1118
ZMB	Zambia	DHS 1996	LI	ESA	4.7	3.3	6.8	676	16.6	14.4	19.0	1923
ZMB	Zambia	DHS 2001	LI	ESA	1.1	0.5	2.3	650	5.9	4.7	7.3	1923
ZMB	Zambia	DHS 2007	LI	ESA	3.5	2.2	5.5	618	12.3	10.6	14.4	1829
ZMB	Zambia	DHS 2013	LMI	ESA	0.9	0.5	1.7	1189	4.8	3.9	5.8	3722

ISO	Country	Survey and year	Income group*	Region of the world**	Animal milk consumption under six months			Number of children	Animal milk consumption between 6-23 months			Number of children
					Prevalence	95% CI			Prevalence	95% CI		
ZMB	Zambia	DHS 2018	LMI	ESA	1.6	0.9	2.8	1019	5.2	4.2	6.5	2785
ZWE	Zimbabwe	DHS 1994	LI	ESA	9.5	6.6	13.6	400	28.0	25.0	31.2	1063
ZWE	Zimbabwe	DHS 1999	LI	ESA	1.3	0.5	3.8	347	11.0	9.1	13.4	1000
ZWE	Zimbabwe	DHS 2005	LI	ESA	11.3	8.3	15.2	534	21.8	18.4	25.6	1445
ZWE	Zimbabwe	MICS 2009	LI	ESA	11.1	8.1	14.9	681	17.9	15.4	20.6	2147
ZWE	Zimbabwe	DHS 2010	LI	ESA	2.7	1.7	4.4	641	8.9	7.4	10.6	1649
ZWE	Zimbabwe	MICS 2014	LI	ESA	4.8	3.5	6.4	867	51.6	49.4	53.9	2965
ZWE	Zimbabwe	DHS 2015	LI	ESA	0.9	0.4	2.2	603	6.6	5.4	8.2	1628
ZWE	Zimbabwe	MICS 2019	LMI	ESA	1.1	0.5	2.4	605	9.4	7.8	11.2	1737

*LI: low income, LMI: lower-middle income, UMI: Upper-middle income.

**EAP: East Asia and Pacific; ESA: Eastern and Southern Africa; ECA: Eastern Europe and Central Asia; LAC: Latin America and Caribbean; MENA: Middle East and North Africa; SA: South Asia; WCA: West and Central Africa.

Supplementary table 4. Annual changes in the prevalence of any breastfeeding indicators at six months and one year, according to the regions of the world.

Indicator	Regions of the world	AAAC*	95% CI		P	Number of countries
		All countries				
Any breastfeeding at six months	West & Central Africa	-0.03	-0.11	0.05	0.408	22
	Eastern & Southern Africa	**-0.12**	**-0.23**	**-0.02**	**0.024**	16
	Middle East & North Africa	-0.47	-0.96	0.03	0.066	6
	Eastern Europe & Central Asia	**0.72**	**0.39**	**1.04**	**0.000**	16
	South Asia	**-0.17**	**-0.27**	**-0.07**	**0.001**	6
	East Asia & Pacific	-0.21	-0.70	0.29	0.412	10
	Latin America & Caribbean	0.31	-0.22	0.83	0.257	19
	North America***	**1.37**	**1.26**	**1.48**	**0.000**	1
	Western Europe	**0.79**	**0.40**	**1.18**	**0.000**	14
Any breastfeeding at one year	West & Central Africa	-0.08	-0.25	0.08	0.308	22
	Eastern & Southern Africa	**-0.18**	**-0.34**	**-0.03**	**0.018**	16
	Middle East & North Africa	-0.29	-0.79	0.21	0.253	7
	Eastern Europe & Central Asia	**0.74**	**0.40**	**1.08**	**0.000**	16
	South Asia	**-0.19**	**-0.30**	**-0.08**	**0.001**	6
	East Asia & Pacific	-0.38	-1.02	0.25	0.241	9
	Latin America & Caribbean	**0.37**	**0.23**	**0.52**	**0.000**	21
	North America***	**1.19**	**1.03**	**1.35**	**0.000**	1
	Western Europe	**0.67**	**0.18**	**1.15**	**0.007**	7

*Absolute average annual changes in percentage points.
**SD: Standard deviation.
***Data available only for the United States.

Supplementary table 5. Annual changes in the prevalence of infant and young child feeding indicators, according to the regions of the world.

Indicator	Regions of the world	AAAC*	95% CI		P	Number of countries
		Low- and middle-income countries				
Exclusive breastfeeding under six months	West & Central Africa	**0.97**	**0.57**	**1.37**	**0.000**	21
	Eastern & Southern Africa	**1.14**	**0.54**	**1.73**	**0.000**	16
	Middle East & North Africa	**-0.67**	**-1.14**	**-0.20**	**0.005**	4
	Eastern Europe & Central Asia	**1.20**	**0.99**	**1.42**	**0.000**	16
	South Asia	**0.51**	**0.34**	**0.68**	**0.000**	6
	East Asia & Pacific	**0.43**	**0.27**	**0.60**	**0.000**	8
	Latin America & Caribbean	**0.76**	**0.16**	**1.35**	**0.013**	12
Formula consumption under six months	West & Central Africa	-0.14	-0.31	0.02	0.079	21
	Eastern & Southern Africa	-0.20	-0.40	0.01	0.056	16
	Middle East & North Africa	**0.65**	**0.10**	**1.19**	**0.019**	4
	Eastern Europe & Central Asia	**0.37**	**0.13**	**0.62**	**0.003**	16
	South Asia	-0.01	-0.08	0.06	0.754	6
	East Asia & Pacific	0.81	-0.05	1.68	0.064	8
	Latin America & Caribbean	0.24	-0.28	0.75	0.371	12
Formula consumption between 6-23 months	West & Central Africa	-0.15	-0.30	0.01	0.073	21
	Eastern & Southern Africa	**-0.21**	**-0.36**	**-0.06**	**0.007**	16
	Middle East & North Africa	0.12	-0.85	1.09	0.810	4
	Eastern Europe & Central Asia	0.57	-0.10	1.23	0.094	16
	South Asia	-0.24	-0.53	0.05	0.104	6
	East Asia & Pacific	**1.03**	**0.18**	**1.87**	**0.017**	8
	Latin America & Caribbean	**0.88**	**0.59**	**1.17**	**0.000**	12
Animal milk consumption under six months	West & Central Africa	**-0.17**	**-0.29**	**-0.05**	**0.004**	21
	Eastern & Southern Africa	-0.46	-0.92	0.00	0.051	16
	Middle East & North Africa	-0.35	-0.71	0.02	0.060	4
	Eastern Europe & Central Asia	**-0.86**	**-1.32**	**-0.41**	**0.000**	16
	South Asia	**-0.42**	**-0.51**	**-0.33**	**0.000**	6
	East Asia & Pacific	**-0.56**	**-0.83**	**-0.29**	**0.000**	8
	Latin America & Caribbean	-0.40	-1.14	0.33	0.283	12
Animal milk consumption between 6-23 months	West & Central Africa	**0.22**	**0.07**	**0.36**	**0.003**	21
	Eastern & Southern Africa	-0.34	-0.85	0.16	0.177	16
	Middle East & North Africa	**-0.95**	**-1.48**	**-0.42**	**0.000**	4
	Eastern Europe & Central Asia	0.11	-1.20	1.43	0.866	16
	South Asia	**-0.32**	**-0.57**	**-0.08**	**0.009**	6
	East Asia & Pacific	0.04	-0.90	0.98	0.935	8
	Latin America & Caribbean	-0.15	-0.77	0.48	0.647	12

*Absolute average annual changes in percentage points.
**SD: Standard deviation.
